# Hip osteoarthritis and occupational mechanical exposures: a systematic review and meta-analysis

**DOI:** 10.5271/sjweh.4152

**Published:** 2024-05-01

**Authors:** Alexander Jahn, Johan Hviid Andersen, Andreas Seidler, David Høyrup Christiansen, Annett Dalbøge

**Affiliations:** 1 Danish Ramazzini Centre, Department of Occupational Medicine, Aarhus University Hospital, Aarhus, Denmark.; 2 Department of Clinical Medicine, Aarhus University, Aarhus, Denmark.; 3 Department of Occupational Medicine - University Research Clinic, Danish Ramazzini Centre, Goedstrup Hospital, Herning, Denmark.; 4 Institute and Policlinic of Occupational and Social Medicine (IPAS), Faculty of Medicine, Technische Universität Dresden, Fetscherstr, 74, 01307 Dresden, Germany.; 5 Elective Surgery Centre, Silkeborg Regional Hospital, Silkeborg, Denmark.; 6 Centre of Health and Nursing Research, Regional Hospital Central Jutland, Viborg, Denmark.

**Keywords:** aetiology, arthritis, musculoskeletal disease, occupational health, physical workload

## Abstract

**Objectives:**

The aim was to conduct a systematic review and meta-analysis investigating the association between occupational mechanical exposures and hip osteoarthritis.

**Methods:**

The study was registered in PROSPERO. A systematic literature search was conducted in six databases to identify relevant articles. Two authors independently excluded articles, extracted data, assessed the risk of bias of each included article, and graded the level of evidence. We conducted a meta-analysis using random-effects model and performed a sensitivity analysis stratifying articles based on the risk of bias assessment, study design, and the outcome measurement.

**Results:**

Twenty-four articles were eligible for inclusion. The highest pooled odds ratio (OR) was found for combined mechanical exposures [OR 1.7, 95% confidence interval (CI) 1.4–2.0], non-neutral postures (OR 1.7, 95% CI 1.4–2.1), lifting/carrying loads (OR 1.6, 95% CI 1.3–1.9), and climbing stairs (OR 1.6, 95% CI 1.1–2.2). The range of pooled OR for the remaining mechanical exposures (eg, standing, walking, kneeling, squatting, and sitting) was 0.6–1.6. Grading the quality of evidence, a moderate level of evidence was found for the combined mechanical exposures and for lifting/carrying loads. The remaining exposure categories were graded as having either low or very low levels of evidence.

**Conclusions:**

Considerable heterogeneity was observed across the included studies, and high-quality literature using objective exposure measurements is warranted. Despite various limitations affecting the comparability, occupational mechanical exposures seem to influence the likelihood of developing hip osteoarthritis.

Osteoarthritis (OA) is a chronic disease causing erosion in the articular cartilage and alterations in the subchondral bone, capsule, and ligaments (1). Almost any joint can be affected by OA, but the condition most often causes problems in the knees, hips, and small joints of the hands. Cardinal symptoms of hip OA consist of pain in or near the hip joint, stiffness, weakness, and audible clicking sounds when moving the hip. Clinical diagnosis of hip OA is made based on cardinal symptoms in combination with imaging modalities (eg, MRI or x-ray) and physical examination (eg, range of motion and tenderness) (2).

Hip OA is considered a global problem having harmful consequences on quality of life, a negative impact on healthcare systems, and an increased risk of years lived with disability (2, 3). European studies have found the prevalence of hip OA to be 2–9% for people <75 years of age (4–8). The global age-standardized incidence proportion of hip OA has increased from 17 per 100 000 persons in 1990 to 18.7 per 100 000 persons in 2019, which corresponds to an estimated annual percentage change of 0.3% (9).

Risk factors associated with hip OA include genetics (10–12), arthritis of other joints (11, 13), age (11, 12), sex (14), body mass index (BMI) (15), waist-to-hip ratio (16), obesity (11, 17), high-impact sports/long-distance running (12, 18, 19), previous trauma (12), and occupational mechanical exposures (20–25). The risk of hip OA has been reported to be higher among workers with high occupational mechanical exposures. In a systematic review from 2022, Unverzagt et al (26) evaluated the influence of occupations with high mechanical exposures on the development of hip OA among men. Based on 11 studies, a higher risk of hip OA was shown for six occupational groups (ie, workers in agriculture, fishery or forestry, food production or sales, construction, metal workers, and men driving vehicles with whole-body vibration). Working in agriculture, including fishery, forestry, and food production, doubled the risk of hip OA. Construction, metalworking, and sales, as well as exposure to whole-body vibration while driving vehicles, increased the risk by roughly 50–60%. Unskilled or basic-level workers, frequently exposed to repetitive heavy manual work, had nearly a doubled risk compared to workers with lower exposure.

Since 2010, six systematic reviews of the association between occupational mechanical exposures and hip OA have been published (20–25). Among the six systematic reviews, lifting loads was the most often studied occupational mechanical exposure, with all six reviews finding an association (20–25). For other occupational mechanical exposures, one or two systematic reviews exist, including very few studies, and meta-analyses were only conducted for lifting loads and exposure to the combination of different mechanical exposures.

In order to provide a comprehensive and exhaustive review of the entire epidemiological evidence without any restrictions to include all possible occupational mechanical exposures, the aim was to conduct a systematic review and meta-analysis of the association between all occupational mechanical exposures and hip OA.

## Methods

### Protocol and study registration

The systematic review with meta-analysis was conducted using guidelines provided by the PRISMA-P 2015 (Preferred Reporting Items for Systematic Reviews and Meta-Analyses) (27, 28). To ensure the methodological quality of our systematic review, we complied with guidelines provided by AMSTAR (Assessing the Methodological Quality of Systematic Reviews) (29). A protocol was registered in the International Prospective Register of Systematic Reviews (PROSPERO) with registration number CRD42022355902.

### Literature search

A systematic literature search was designed, tested, and performed in collaboration with a research librarian. It was optimized for each specific database and its syntax, carried out in the National Library of Medicine (Medline), Excerpta Medica Database (EMBASE), PsycINFO, Cumulative Index to Nursing and Allied Health Literature (CINAHL), Cochrane Library, and Web of Science between 31 May and 23 June 2022. The literature search from MEDLINE is presented in the supplementary material (www.sjweh.fi/article/4152) Appendix 1. Our literature search was supplemented by hand-searching all bibliographies of reviews published after 2010 and the included articles. Finally, by using the Google Scholar search engine, we searched for literature by screening the first 100 hits for potentially relevant articles. Afterward, two review authors selected relevant articles using the Covidence systematic review software and independently screened all articles using a two-step model. At first, articles were screened based on title/abstract followed by full-text reading. A third review author resolved any disagreements between the two review authors.

### Study inclusion criteria

Study inclusion criteria were described based on components of PECOS (population, exposure, comparison, outcome, and study design). We included studies with a population in or above the working age. The exposure included all occupational mechanical exposures assessed using self-report, observations, expert ratings, technical measures, job exposure matrices, or combinations. Exposure assessments based solely on proxy measures without any form of assessment of the mechanical exposure (eg, job titles) were excluded. The comparison was defined as a measure of association between occupational mechanical exposures and hip OA, or one possible to calculate, consisting of, eg, an exposed versus non/low exposed group. Measures of association comprised relative risks (RR), odds ratios (OR), hazard ratios (HR), and prevalence ratios (PR).

We included studies that defined hip OA in accordance with the following criteria: (i) diagnosis according to criteria stated by the American College of Rheumatology, (ii) ICD-codes or diagnosis from registers, (iii) hip replacement caused by OA, (iv) radiographic diagnosis according to, eg, Kellgren and Lawrence, (v) hip pain with physical examination measuring stiffness and physical limitations, and (vi) self-reported hip OA. If the outcome consisted of a composite anatomical site, eg, lower-body OA, was caused by trauma or inherent pain, or the diagnosis was solely based on hip pain, the study was excluded. Furthermore, studies based on admissions or surgery codes with OA secondary to other diseases, such as rheumatoid arthritis, were excluded. The eligible study designs were randomized controlled trials and observational studies. Each study should include ≥30 persons and be written in English or a Scandinavian language.

### Data extraction and risk of bias assessment

Two data extraction tables were predefined, one containing the descriptive information (ie, author, study design, population, outcome definition, outcome assessment, exposure definition, and exposure assessment) and one containing the analytical information [confounders, exposure groups, measure of associations, and confidence intervals (CI)]. One author extracted all relevant data from the included studies. Three other authors quality-checked the extraction, and a third resolved any disagreements in the data extraction.

To critically appraise the methodological quality, we used a risk of bias tool developed for chronic diseases used in several previous systematic reviews (Appendix 2) (30–35). The risk of bias tool consisted of five major risk domains and three minor risk domains. Based on ratings from all domains, the overall risk of bias of each included study was rated as low, moderate, or high risk of bias. A study was considered to have low risk of bias if all major domains and at least one minor domain were rated as low risk of bias. For a study to be considered to have a moderate risk of bias, four out of five major domains and at least one minor domain should be rated as low risk of bias. All other combinations were considered as high risk of bias.

Two authors indepdendently performed the risk of bias assessment. Afterward, all risk of bias assessments were compared and if the individual assessments differed, the risk of bias assessments were discussed with all authors until a consensus was reached.

### Statistical analysis

The meta-analysis, including forest plots, was conducted using OR to visualize whether an association between occupational mechanical exposures and hip OA across studies could be indicated. Before conducting the meta-analysis, studies based on identical source populations were excluded to avoid double-counting data. If an identical source population occurred, we excluded the study with the highest risk of bias, and, if both studies had the same risk of bias assessment, the study based on the smallest sample was excluded. Furthermore, if a study provided a measure of association other than OR, it was considered equivalent to an OR if the incidence proportion of the outcome was <10% (36). In addition, if a study had no measure of association but provided sufficient information on the number of participants in each exposure group, we calculated the OR with its corresponding 95% CI. We included the measure of association for the highest exposure group versus the lowest exposure group. The selection of relevant measures of association was based on a hierarchical approach: (I) high contrast between exposure groups, (II) the most adjusted measure of association, and (III) the measure of association containing most participants.

For each exposure category, pooled estimates were calculated using random-effects model (37). Heterogeneity between studies was calculated using I^2^ statistics, quantified by the restricted maximum likelihood method (38), and was interpreted using Cochrane’s thresholds for interpretation of the I^2^ statistics (39). Publication bias was evaluated by using funnel plots, and the asymmetry of funnel plots was tested using Egger’s test (40). Exposure–response relations were examined by extracting results from statistical tests (eg, trend test) provided in a study. If an exposure–response relation was not statistically examined, we constructed scatter plots including the OR and 95% CI for each level of exposure from studies providing >3 exposure groups that graphically indicated whether an exposure–response relation existed. Finally, sensitivity analyses were conducted by repeating the meta-analyses stratifying according to the risk of bias assessments (low/moderate versus high risk of bias). In order to evaluate the results, we also stratified based on study design (cohort/case–control versus cross-sectional), outcome measurements (total hip replacement versus other outcomes) and sex differences. All analyses were performed using STATA 17.0 (Stata Corp, College Station, TX, USA) using the ‘meta’ command for performing the meta-analyses.

### Evidence of an association

The quality of evidence was assessed separately for each exposure category using the Navigation Guide methodology (41) considering observational epidemiological studies in occupational and environmental health. This approach was based on the Grading of Recommendations Assessment, Development, and Evaluation (GRADE) (42). We downgraded the quality of evidence based on the risk of bias, inconsistency, indirectness, imprecision, and publication bias. We upgraded the quality of evidence based on magnitude of effect, dose-response, and residual confounding. By applying guidelines from the Navigation Guide, the level of evidence from observational studies started at “moderate” evidence. Two authors independently assessed the level of evidence, and a third author was consulted if discrepancies occurred between ratings. The overall level of evidence could be rated as “high”, “moderate”, “low”, or “very low” (Appendix 3).

## Results

### Study selection and characteristics

**Figure 1:**
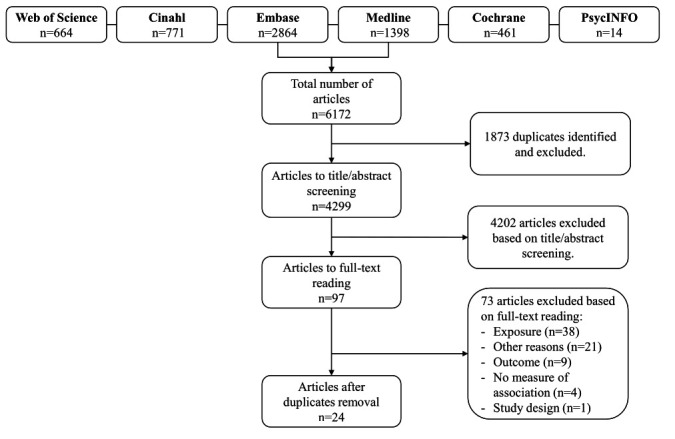
Flowchart of the study inclusion

Figure 1 presents the flow chart of the literature search and exclusion of articles. The literature search yielded 6172 articles identified from the six scientific databases, including 1873 duplicates. A total of 4299 articles were screened based on title/abstract, which led to the exclusion of 4202 articles. After 97 full-text readings, 24 articles were found eligible for inclusion. Reasons for exclusion based on our full-text reading are provided in Appendix 4.

Appendix 5 summarizes the descriptive characteristics of the 24 included articles (43–66). In total, six cohort studies, 13 case–control studies, and five cross-sectional studies were included. The outcome was assessed using imaging modalities in ten studies, data from registers (ICD-codes or hip-replacement-records) in seven studies, a combination of imaging modalities and register data in two studies, clinical examinations in two studies, a questionnaire in one study, a combination of questionnaire and data from general practitioners in one study, and information on total hip replacements gathered directly from orthopedic clinics in one study. Information on occupational mechanical exposures was assessed using questionnaires in 12 studies, interviews in seven studies, and job-exposure matrices (JEM)/expert ratings in five studies. The studies were conducted in Denmark (60, 61), Sweden (49, 55, 63–65), Norway (47), Finland (48, 50–52, 62), England (44, 45), Netherlands (57, 58), Croatia (46), United States (43, 59), Canada (56), Hong Kong (53, 54), and Japan (66) and published between 1987 and 2020.

### Risk of bias assessment

Table 1 presents the risk of bias assessment. In summary, two studies were assessed as having a low risk of bias, six as having a moderate risk of bias, and sixteen as having a high risk of bias. The most frequent major domains receiving a low risk of bias assessment were “outcome” followed by “analysis method”. Conversely, the most frequent major domains receiving high risk of bias assessment were “exposure” followed by “enrolment/participants”.

**Table 1:** Risk of bias assessment of the 24 included studies. [✔=comply with criteria; ×=does not comply with criteria; ?= no information was provided].

References	Quality score (risk)	Domains								
		Major						Minor		
		Study design and selection	Exposure	Outcome	Enrolment or non-participants	Analysis method^a^		Funding	Chronology	Conflict of interest
Allen, 2010 (43)	High	✔	×	✔	×	✔		✔	✔	✔
Coggon, 1988 (44)	High	✔	×	✔	×	✔		✔	✔	**?**
Croft, 1992 (45)	High	✔	×	×	×	×		✔	✔	**?**
Cvijetic, 1999 (46)	High	×	✔	✔	×	×		×	✔	**?**
Flugsrud, 2002 (47)	Moderate	✔	×	✔	✔	✔		✔	✔	✔
Heliovaara, 1993 (48)	High	✔	×	✔	×	✔		×	×	**?**
Jacobsson, 1987 (49)	High	×	×	✔	**?**	×		×	×	**?**
Juhakoski, 2009 (50)	High	×	×	✔	✔	✔		✔	✔	✔
Kaila-Kangas, 2011 (51)	High	✔	×	✔	×	✔		✔	×	✔
Kontio, 2020 (52)	Moderate	✔	×	✔	✔	✔		✔	✔	✔
Lau, 2000 (53)	High	×	×	✔	**?**	✔		✔	✔	**?**
Lau, 2007 (54)	High	×	×	✔	✔	✔		✔	×	**?**
Olsen, 1994 (55)	High	×	×	✔	×	✔		✔	✔	**?**
Ratzlaff, 2011 (56)	Low	✔	✔	✔	✔	✔		✔	✔	✔
Rijs, 2014 (57)	High	×	×	✔	✔	×		✔	✔	✔
Riyazi, 2008 (58)	High	×	×	✔	×	✔		✔	×	✔
Roach, 1994 (59)	High	×	×	✔	✔	✔		✔	✔	**?**
Rubak, 2013 (60)	Low	✔	✔	✔	✔	✔		✔	✔	**?**
Rubak, 2014 (61)	Moderate	✔	✔	✔	×	✔		✔	✔	**?**
Solovieva, 2018 (62)	Moderate	✔	✔	✔	×	✔		✔	✔	**?**
Thelin, 1997 (63)	High	✔	×	✔	✔	×		✔	✔	**?**
Vingård, 1991 (64)	Moderate	✔	✔	✔	×	✔		✔	✔	**?**
Vingård, 1997 (65)	Moderate	✔	×	✔	✔	✔		✔	✔	×
Yoshimura, 2000 (66)	High	✔	×	✔	×	✔		✔	✔	**?**

^a^ Minor domains comprised.

### Association between occupational mechanical exposures and hip osteoarthritis

Measures of association between occupational mechanical exposures and hip OA reported in the twenty-four studies are presented in Appendix 6. Association was measured using HR in threes (52, 56, 62) and RR in an additional three studies (47, 64, 65). Based on the assumption that an incidence proportion of an outcome <10% can approximate an OR, the measure of association from all six studies was treated equally as an OR (36). Furthermore, two (43, 49) did not provide a risk estimate but gave sufficient information to calculate an OR with a 95% CI. The ‘combined mechanical exposure’ category consisted of occupational mechanical exposures that refer to the simultaneous impact of various mechanical exposures workers may encounter, eg, forceful exertions, repetitive hand movements, vibrations, or lifting/carrying loads.

### Lifting/carrying loads

**Figure 2:**
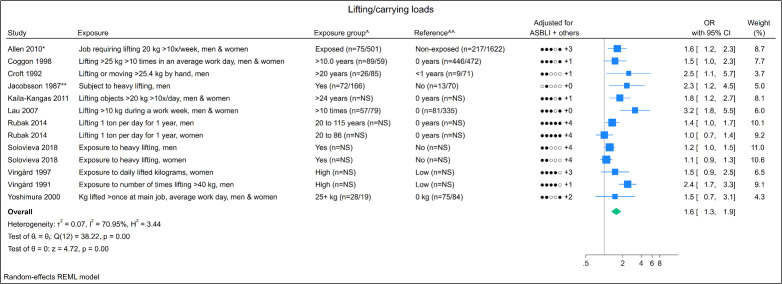
Forest plot of exposure to lifting/carrying loads. **Notes:** adjusted variables (ASBLI) = age, sex, body mass index, leisure time activities, and previous injuries in lower extremities. + others refer to adjusting for other confounding factors besides the ASBLI-factors. [kg=kilograms; NS=not specified; x=times; OR=odds ratio.] * Allen 2010: OR calculated based on prevalence of distribution between groups (table 4 in the study). ** Jacobsson 1987: OR calculated based on numbers of participants (table 1 in the study). ^ Numbers in brackets states numbers of exposed persons with hip OA and numbers of exposed references, respectively. ^^ Numbers in brackets states numbers of unexposed persons with hip OA and numbers of unexposed references, respectively.

Of the thirteen studies, four had a moderate risk of bias and the remaining nine a high risk of bias. We noted that two (53, 54) used the same study population, hence we excluded one (53) from the meta-analysis. Furthermore, one study (55) did not provide a 95% CI pertaining to the measure of association and was also excluded. We found a pooled OR of 1.6 (95% CI 1.3–1.9), showing a substantial degree of heterogeneity (I^2^=70.9%) (Appendix 9, Figure 13). Among eight studies presenting a measure of association containing >3 exposure groups, scatter plots of six indicated an increase in OR with increasing exposure levels (Appendix 8, Figure 2). Grading the quality of evidence, a moderate level of evidence was found for exposure to lifting/carrying loads (Appendix 3, Table 3).

### Standing

**Figure 3:**
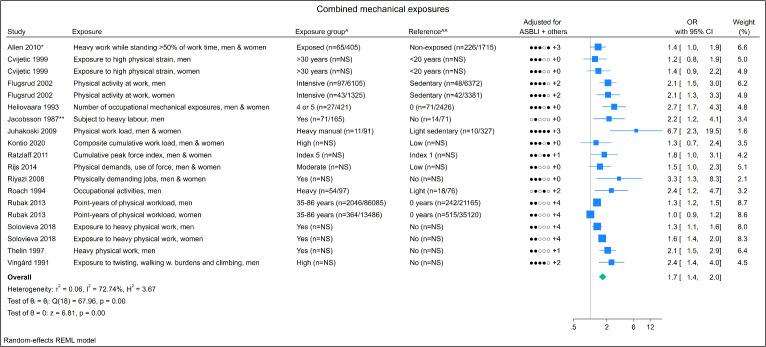
Forest plot of combined mechanical exposures. **Notes:** adjusted variables (ASBLI) = age, sex, body mass index, leisure time activities, and previous injuries in lower extremities. + others refer to adjusting for other confounding factors besides the ASBLI-factors. [kg=kilograms; NS=not specified; x=times; OR=odds ratio.] * Allen 2010: OR calculated based on prevalence of distribution between groups (table 4 in the study). ** Jacobsson 1987: OR calculated based on numbers of participants (table 1 in the study). ^ Numbers in brackets states numbers of exposed persons with hip OA and numbers of exposed references, respectively. ^^ Numbers in brackets states numbers of unexposed persons with hip OA and numbers of unexposed references, respectively.

Of the seven studies, one was rated as having a moderate and six a high risk of bias. We found a pooled OR of 1.3 (95% CI 1.0–1.8) and an I^2^ value of 44.7%, indicating a moderate to minimal degree of heterogeneity (Appendix 9, Figure 14). Among four studies, scatter plots of three indicated an increase in OR with increasing exposure levels (Appendix 8, Figure 3). Grading the quality of evidence, a very low level of evidence was found for exposure to standing (Appendix 3, Table 3).

### Walking

All seven studies were rated as having a high risk of bias and two (53, 54) had identical populations, hence one (53) was excluded. We found a pooled OR of 1.3 (95% CI 1.1–1.5) (Appendix 9, Figure 15) and an I^2^ value of 0% indicating no observed heterogeneity. Among two studies, both scatter plots indicated an increase in OR with increasing exposure levels (Appendix 8, Figure 4). Grading the quality of evidence, a very low level of evidence was found for exposure to walking (Appendix 3, Table 3).

### Climbing stairs

Of the seven studies, one was rated as having a moderate and six a high risk of bias. Of the six eligible studies, two (53, 54) had identical populations, hence one (53) was excluded. We found a pooled OR of 1.6 (95% CI 1.1–2.2) and an I^2^ value of 49.8%, indicating a moderate degree of heterogeneity (Appendix 9, Figure 16). Scatter plots in two studies indicated a positive exposure-response relation (Appendix 8, Figure 5). Grading the quality of evidence, a low level of evidence was found for exposure to climbing stairs (Appendix 3, Table 3).

### Non-neutral postures

Of the five studies, two were rated as having a moderate and three a high risk of bias. We found a pooled OR of 1.7 (95% CI 1.4–2.1) (Appendix 9, Figure 17) and an I^2^ value of 5.6% indicating almost no observed heterogeneity. Among three studies, all scatter plots indicated an increased OR with increasing exposure (Appendix 8, Figure 6). Grading the quality of evidence, a low level of evidence was found for non-neutral postures (Appendix 3, Table 3).

### Sitting

Of the six studies, two were rated as having a moderate and four a high risk of bias. No identical populations were observed, but one study did not provide a 95% CI to the pertaining measure of association and was excluded from the meta-analysis. We found a pooled OR of 0.6 (95% CI 0.5–0.9) and an I^2^ value of 78.2%, indicating substantial degree of heterogeneity (Appendix 9, Figure 18). Scatter plots in two studies did not indicate a positive exposure–response relation (Appendix 8, Figure 7). Grading the quality of evidence, a low level of evidence was found for exposure to sitting (Appendix 3, Table 3).

### Kneeling

All six studies were rated as having a high risk of bias, and two (53, 54) had identical populations, hence one (53) was excluded. We found a pooled OR of 1.2 (95% CI 0.9–1.5) (Appendix 9, Figure 19) and an I^2^ value of 0% indicating no observed heterogeneity. Scatter plots in two studies did not indicate a positive exposure-response relation (Appendix 8, Figure 8). Grading the quality of evidence, a very low level of evidence was found for exposure to kneeling (Appendix 3, Table 3).

### Squatting

All six studies were rated as having a high risk of bias, and two (53, 54) had identical populations, hence one (53) was excluded. We found a pooled OR of 1.1 (95% CI 0.9–1.4) (Appendix 9, Figure 20) and an I^2^ value of 0% indicating no observed heterogeneity. In one study, the scatter plot did not indicate a positive exposure-response relation (Appendix 8, Figure 9). Grading the quality of evidence, a very low level of evidence was found for exposure to squatting (Appendix 3, Table 3).

### Standing/walking

All three studies were assessed as having a high risk of bias, and no identical populations were observed. We found a pooled OR of 1.1 (95% CI 1.0–1.2) (Appendix 9, Figure 21) and an I^2^ value of 0% indicating no observed heterogeneity. Among two studies, the scatter plots did not indicate a positive exposure-response relation (Appendix 8, Figure 10). Grading the quality of evidence, a low level of evidence was found for exposure to standing/walking (Appendix 3, Table 3).

### Kneeling/squatting

Both studies were assessed as having a moderate risk of bias, and no identical populations were observed. We found a pooled OR of 1.3 (95% CI 1.1–1.7) (Appendix 9, Figure 22) and an I^2^ value of 56.58% indicating moderate degree of heterogeneity. In one study, the scatter plot did not indicate an exposure–response relation (Appendix 8, Figure 11). Grading the quality of evidence, a low level of evidence was found for exposure to kneeling/squatting (Appendix 3, Table 3).

### Combined mechanical exposures

Of the fifteen studies, two were rated as having a low, four a moderate, and nine a high risk of bias. No identical populations were observed. The meta-analysis showed a pooled OR of 1.7 (95% CI 1.4–2.0) and an I^2^ value of 72.7%, indicating a substantial degree of heterogeneity (Appendix 9, Figure 23). Results of the sensitivity analysis, GRADE, and publication bias are provided in table 2 and the funnel plots in Appendix 7. Flugsrud et al (47) and Heliovaara et al (48) found significant trend tests while six out of eight scatter plots studies containing >3 exposure groups indicated a positive exposure–response relation (Appendix 8, Figure 12). Grading the quality of evidence, a moderate level of evidence was found for the combined exposures (Appendix 3, Table 3).

**Table 2:** Overview of pooled odds ratios, publication bias, and level of evidence between each occupational mechanical exposure and hip osteoarthritis based on studies included in the meta-analysis. [CI=confidence interval; OR=odds ratio].

Mechanical exposures in meta-analysis	N of studies	Pooled	Sensitivity analysis															Level of evidence of an association^a^
			Risk of bias				**Study design**				**Outcome**				**Sex**			
			Low/ moderate		High		**Cohort/ case–control**		**Cross-sectional**		**Hip replace**		**Other outcomes**		**Men**		**Women**	
		OR (95% CI)	OR (N)		OR (N)		**OR (N)**		**OR (N)**		**OR (N)**		**OR (N)**		**OR (N)**		**OR (N)**	
Lifting/carrying loads	11	1.6 (95% CI 1.3–1.9)	1.3 (4)		1.8 (7)		**1.6 (10)**		**1.6 (1)**		**1.6 (4)**		**1.7 (7)**		**1.9 (8)**		**1.3 (6)**	**Moderate (+++).**
Standing	7	1.3 (95% CI 1.0–1.8)	1.6 (1)		1.3 (6)		**1.2 (5)**		**1.6 (2)**		**1.8 (2)**		**1.2 (5)**		**1.2 (4)**		**1.6 (3)**	**Very low (+).**
Walking	6	1.3 (95% CI 1.1–1.5	(0)		1.3 (6)		**1.3 (5)**		**1.2 (1)**		**1.6 (1)**		**1.3 (5)**		**1.4 (4)**		**1.2 (2)**	**Very low (+).**
Climbing stairs	6	1.6 (95% CI 1.1–2.2)	2.1 (1)		1.5 (5)		**1.8 (5)**		**1.2 (1)**		**1.7 (2)**		**1.6 (4)**		**2.4 (3)**		**2.2 (3)**	**Low (++).**
Non-neutral postures	5	1.7 (95% CI 1.4–2.1)	2.2 (2)		1.6 (3)		**2.1 (3)**		**1.6 (2)**		**2.1 (3)**		**1.6 (2)**		**2.6 (2)**		**1.6 (1)**	**Low (++).**
Sitting	5	0.6 (95% CI 0.5–0.9)	0.5 (1)		0.8 (4)		**0.6 (4)**		**0.8 (1)**		**0.8 (1)**		**0.6 (4)**		**0.7 (3)**		**0.6 (2)**	**Low (++).**
Kneeling	5	1.2 (95% CI 0.9–1.5)	(0)		1.2 (5)		**1.2 (4)**		**1.1 (1)**		**1.0 (1)**		**1.2 (4)**		**1.1 (3)**		**1.4 (2)**	**Very low (+).**
Squatting	5	1.1 (95% CI 0.9–1.4)	(0)		1.1 (5)		**1.2 (4)**		**1.1 (1)**		**1.3 (1)**		**1.1 (4)**		**1.0 (3)**		**1.2 (2)**	**Very low (+).**
Standing/walking	3	1.1 (95% CI 1.0–1.2)	1.1 (3)		(0)		**1.1 (3)**		**(0)**		**1.0 (1)**		**1.2 (3)**		**1.1 (2)**		**1.1 (2)**	**Low (++).**
Kneeling squatting	3	1.3 (95% CI 1.1–1.7)	1.3 (2)		(0)		**1.3 (2)**		**(0)**		**(0)**		**1.3 (2)**		**1.2 (1)**		**1.5 (1)**	**Low (++).**
Combined exposures	15	1.7 (95% CI 1.4–2.0)	1.5 6)		1.9 (9)		**1.8 (11)**		**1.6 (4)**		**1.7 4)**		**1.6 (11)**		**1.7 (8)**		**1.4 (4)**	**High (++++).**

^a^ The combined exposures were the only category containing moderate and low risk of bias studies in the sensitivity analysis.

### Sensitivity analyses and publication bias

Table 2 presents an overview of the results from our sensitivity analyses, publication bias, and level of evidence. In general, higher pooled OR were found in the cohort/case–control design compared to the cross-sectional design, studies with outcomes defined as hip replacement, but no clear trend was observed in low/moderate versus high risk of bias studies. Estimating sex differences, men tended to have higher OR compared to women. Indication of publication bias was observed in three exposure categories, two funnel plots were difficult to interpret due to few studies included, and six exposure categories did not indicate publication bias. Funnel plots for each mechanical exposure are included in Appendix 7.

## Discussion

### Main results

Twenty-four studies were included in this systematic review with meta-analysis. Based on the quality of evidence, we found a moderate level of evidence for the combined occupational mechanical exposures with a pooled OR of 1.7 (95% CI 1.4–2.0) and for exposure to lifting/carrying loads with a pooled OR of 1.6 (95% CI 1.3–1.9). A low or very low level of evidence was found for the remaining exposure categories with pooled OR of 1.1–1.7, while exposure to sitting could indicate a protective effect [OR of 0.6, (95% CI 0.5–0.9)].

### Methodological considerations

Several methodological considerations affecting the meta-analyses should be discussed, eg, exposure, outcome, and study design. First, in relation to the exposure, a meta-analysis requires similarities in exposure definition, metrics, and assessment between studies, which was not observed. In general, exposure definition was highly heterogeneously defined. For instance, lifting/carrying loads was defined as lifting loads >10, 20, 25, 40, or 50 kg, repetitively lifting during a workday or week, exposed to lifting, and exposed to heavy lifting with or without an indication of kilograms or repetitions. The exposure metric ranged from a dichotomous approach (yes/no), exposure duration (years being exposed) to a specification of intensity and frequency during a week or month, reducing the comparability between studies. Despite large diversities, we presented pooled OR to visualize whether an association between occupational mechanical exposures and hip OA could be indicated across all studies. In addition, the meta-analyses for some exposure variables (eg, standing/walking and kneeling/squatting) and several of the sensitivity analyses were conducted with few studies. Therefore, the pooled OR should be interpreted with caution.

No clear direction of effect was observed when comparing low/moderate risk of bias studies with high risk of bias studies. By combining moderate and low risk of bias studies, the combination might have been less beneficial since one major domain is affected by bias in the moderate risk of bias. However, only the combined exposure category contained both low and moderate risk of bias studies. All other exposure categories compared moderate versus high risk of bias studies.

In the meta-analysis, the measure of association comparing the highest versus lowest exposure groups was chosen to ensure exposure contrast. However, the highest exposure groups often contained fewer participants, affecting the standard error of a given estimate, resulting in broader CI with an increased risk of type 2 errors. In addition, grouping into ever/high versus never/low might increase the risk of underestimating any potential association considerably. Even though, among the 11 occupational mechanical exposures included in the meta-analyses, statistically significant pooled OR were found for eight occupational mechanical exposures (ie, lifting/carrying loads, non-neutral postures, standing, walking, standing/walking, kneeling/squatting, climbing stairs, sitting, and combined mechanical exposures).

The exposure assessment was often based on self-reports, ie, a questionnaire or interview. Such assessment methods can be affected by recall bias, especially when information on the exposure is gathered over decades of work, potentially contributing to exposure misclassification (67). Five studies used JEM/expert ratings, typically combining self-reported job titles or register-based International Classification of Occupations (ISCO) codes with the JEM. JEM typically assign exposures at a qualitative or semi-quantitative level based on expert ratings, and any misclassification is expected to be non-differential with respect to the outcome. By design, a JEM allocates the same exposure estimates to all workers with the same job title or ISCO code (group-based). Exposure–response relations have been shown to be essentially unbiased with group-based exposures, while individual-based models, where everyone is assigned to his/her exposure under a classical error structure, lead to attenuated slopes unless everyone is measured extensively (68, 69). This advantage of the group-based strategy comes, however, at the price of an increased uncertainty of the regression coefficient and thus reduced power, ie, reduced ability of a study design to detect a true effect of exposure on outcome (68, 69).

Overall, the heterogeneity in exposure definition, metrics, and assessment, indicated by the generally high I^2^ values in the meta-analyses, reduced the possibility of comparing studies.

Second, criteria for hip OA described in the included studies varied from cases of total hip replacements, radiographic data assessing joint space, registers gathering information on OA based on ICD-codes, to clinical examinations. Based on a few studies, we generally found higher pooled OR in studies with outcomes defined as total hip replacements. This could be due to an increased risk of total hip replacements, an increased risk of surgery given hip OA, or both. For non-surgery-treated hip OA, a combination of radiographic and clinical examination is considered best for discriminating between hip OA and hip pain due to other causes (70). Misclassification of the outcome might, however, occur especially for participants with less severe hip OA. A study from 2015 found that most patients with frequent hip pain did not have radiographic hip OA, and most patients with radiographic-confirmed hip OA did not have frequent hip pain (71).

Third, heterogeneity was also observed according to the number of confounders adjusted for in each of the included studies. Overall, the number of controlling confounders varied from 0 to 9 (mean 4.7), potentially explaining some of the dispersion observed. Most studies controlled for age (94%) and sex (75%), followed by BMI (55%) and previous hip injury (47%), while few studies controlled for other occupational mechanical exposures (62). Occupational mechanical exposures often co-occur, which might confound and over-estimate measure of association when not controlled for. For instance, in the study of Solovieva et al (62), we chose to extract data adjusted for other occupational mechanical exposures with a higher risk of over-adjustment. If we had included the age-adjusted estimates, higher pooled OR would have been found for lifting/carrying loads, standing/walking, kneeling/squatting, and combined mechanical exposures, while lower pooled OR would have been found for sitting (more protective effect). For lifting/carrying loads and combined mechanical exposure, a doubling of risk could have been found. Despite the obvious heterogeneity between the inclusion of confounding variables in the statistical analyses, lifting/carrying loads and combined mechanical exposures were assessed as having a moderate level of evidence since all estimates point towards an increased risk of developing hip OA. It is also important to note that only two studies were assessed as having a low risk of bias.

Finally, heterogeneity also occurred according to study design, study population, number of included participants, and risk of bias assessment. Among the 24 studies, only four studies were cross-sectional studies where temporality between exposure and outcome cannot be ensured. The study population varied and consisted of, eg, farmers, community-dwelling members, veterans, as well as diverse representative populations. Several of the studies included in the review comprised representative populations, while few studies specifically selected highly exposed workers such as scaffolders or carpenters. Studies with highly exposed workers are warranted to evaluate the maximum strength of association.

To evaluate the effect of the heterogeneity, several additional sensitivity analyses were conducted investigating differences in study design, risk of bias, outcome measures, and sex. Based on a few studies, generally higher pooled OR were found in cohort/case–control studies, studies with outcomes defined as total hip replacement, no clear trend for a measure of association was found for low/moderate versus high risk of bias studies, and men tended to have higher OR than women.

### Comparing results

Previously published systematic reviews since 2010 on the association between occupational mechanical exposures and hip osteoarthritis have mostly included few occupational mechanical exposure categories (20–25). A lack of both the inclusion of several occupational mechanical exposures, inclusion of relevant articles, and meta-analyses were observed. However, the results of our systematic review correspond with existing results found in previous systematic reviews. Furthermore, our results indicate a possible association between other occupational mechanical exposures, eg, non-neutral postures and climbing stairs, and a protective association between sitting and hip OA.

In the systematic review of Seidler et al (ref), an external reference population was used to determine the exposure–response relation between lifting/carrying loads and hip OA. With the basis in six studies, the risk of developing hip OA was increased by an OR of 1.98 (95% CI 1.20–3.29) per 10 000 tons of weights ≥20 kg handled, an OR of 2.08 (95% CI 1.22–3.53) per 10 000 tons handled >10 times per day and an OR of 8.64 (95% CI 1.87–39.91) per 106 operations. In women, there was no linear association between manual handling of weights at work and the risk to developing hip OA based on five studies. As previously mentioned, obvious limitations arise in the methodological quality of the epidemiological studies assessing occupational mechanical exposures. However, the derivation of a exposure–response relation is of high importance despite these limitations and underpins the need for preventive strategies to ensure a healthy and safe work environment.

Our aim was to synthesize the existing epidemiological evidence, highlighting the strengths and weaknesses of each included study through a risk of bias assessment. We acknowledge that the meta-analyses, hence the forest plots, comprise huge problems, why they are only used for the visual presentation of results and should be interpreted with caution. However, it is essential that an overview of the entire epidemiological evidence is presented so that new emerging scientific evidence can be planned/carried out to improve our understanding, and more importantly, enhance the scientific quality. The same problem has been underpinned by the author group before (35). We consider it a strength that our systematic review included several studies compared to previous reviews. Despite obvious differences, the results presented in our review do align with the results presented from previous systematic reviews.

### Suggestions for future research and practical implications

Research on chronic diseases developing over time requires studies accounting for the time lag between exposure, possible symptoms, and the onset of disease. We suggest that future research utilizes already large established cohorts with a prolonged longitudinal approach [eg, DOC*X cohort (72)], eventually incorporating register-based information. Registers can, to some extent, provide reliable information on confounding factors; knowledge of a participant’s job rotation/work participation history, and high-quality information on disease status.

There is a strong association between hip OA and age, and therefore, newer statistical methods to study the effect of occupational mechanical exposures on hip OA are recommended. Risk and rate advancement periods (RAP) measure the impact of exposure on the relation of age to disease. Specifically, they quantify the time by which the risk or rate of a disease is advanced among exposed subjects conditional on disease-free survival to a certain baseline age, thereby studying if workers with physically demanding work attract their hip OA at an earlier age than workers with less physically demanding work (73).

### Concluding remarks

Our systematic review revealed considerable heterogeneity across studies and exposures measured subjectively. Given the large amount of literature, more high-quality literature is warranted as well as quantitative, objective measurements of the exposures. Despite various limitations, we found a moderate level of evidence for the combined occupational mechanical exposures and lifting/carrying loads. Low or very low levels of evidence were found for the remaining mechanical exposures, while exposure to sitting could indicate a protective effect.

## Supplementary material

Supplementary material

## Data Availability

The data and material included in this review are available upon reasonable requests.
