# Supplementary material for: Hip osteoarthritis and occupational mechanical exposures: a systematic review and meta-analysis
Source: Scand J Work Environ Health. 2024 Apr 29;50(4):244–56. doi: 10.5271/sjweh.4152 (PMC11129821; doi:10.5271/sjweh.4152)
Supplement: Supplementary material [file SJWEH-50-244-S001.pdf]

Supplemental material

Hip osteoarthritis and occupational mechanical exposures: a systematic review and meta-analysis

Alexander Jahn, MSc, Johan Hviid Andersen, PhD, Andreas Seidler, MD, MPH, David Høyrup Christiansen, PhD,

Annett Dalbøge, PhD

Contents

Appendix 1. Literature search 3

Aggregated search string from Medline 3

Appendix 2. Risk of bias tool........ 7

Table 1......... 7

Appendix 3. Grading the evidence of an association 10

Table 2. Quality level - four levels of evidence. 10

Table 3. Evaluating the quality of evidence - down- and upgrading factors........ 10

Table 4. The GRADE assessment. 12

Appendix 4. Excluded articles from full-text reading........ 14

Table 5. Excluded articles. 14

Appendix 5. Characteristics of the included 24 studies. 18

Table 6. Description of each included article........ 18

Appendix 6. Data extraction regarding the measure of association 26

Table 7. Data extraction of the measure of association........ 26

Appendix 7. Funnel plots 46

Figure 2. Funnel plot of all included exposure categories. 46

Appendix 8. Scatter plots 47

Figure 3. Lifting/carrying loads 47

Figure 4. Standing. 48

Figure 5. Walking........ 49

Figure 6. Climbing stairs 50

Figure 7. Non-neutral postures........ 51

Figure 8. Sitting........ 52

Figure 9. Kneeling 53
Figure 10. Squatting 54
Figure 11. Standing/walking 55
Figure 12. Kneeling/squatting 56
Figure 13. Combined exposures 57
Appendix 9. Forest plots 58
Figure 14. Standing 58
Figure 15. Walking 59
Figure 16. Climbing stairs 60
Figure 17. Non-neutral postures 61
Figure 18. Sitting 62
Figure 19. Kneeling 63
Figure 20. Squatting 64
Figure 21. Standing/walking 65
Figure 22. Kneeling/squatting 66

Appendix 1. Literature search

Aggregated search string from Medline

(((((Hip injuries/ or hip/ or (hip or hips or coxa*).ab,kf,ti.) and (osteoarthritis/ or (osteoarthr* or "degenerative arthr*" or "osteo-arthr*").ab,kf,ti.)) or (Osteoarthritis, hip/ or (coxarthr* or (osteoarthr* adj2 (hip or hips))).ab,kf,ti.)) and (exp Occupational Exposure/ or exp Occupational Disease/ or Lifting/ or Workload/ or Workplace/ or weight-bearing/ or occupational groups/ or farmers/ or Agricultural workers disease/ or Construction industry/ or exp Work/ or exp Employment/ or Industry/ or exp Commerce/ or cold temperature/ or hot temperature/ or ("Axial loading*" or "Heavy lifting" or "Job site*" or weightbearing or loadbearing or "Weight-bearing" or "load-bearing" or strain or carrying or lifting or "work load*" or "work location*" or "work place*" or "work site*" or workload or workplace* or worksite* or workabilit* or "work abilit*" or "repetitive work" or Squatting or bending or Kneeling or Occupation or Vocation* or employee* or "Occupational group*" or Personnel or worker or Workers or employment or "Labor force" or "labour force" or "Labor market" or "Labour market" or "Occupational status" or Underemployment or business* or commerc* or vendor* or (construction adj1 (trade* or industry*)) or (building adj1 (trade* or industry*)) or (agricultural worker* adj2 disease*) or (physical* adj2 (work or job)) or (occupational adj2 (disease* or exposure* or illness*))).ab,kf,ti.)) not (exp animal/ not exp human/)) and (danish or english or swedish or norwegian).lg.

Notes in relation to Figure 1:
The Adjacent operator (ADJ) retrieves records with search terms next to each other in that specific order. You do not need to separate search terms manually by inserting ADJ between them, because when you separate terms with a space on the command line, Ovid automatically searches for the terms adjacent to
one another.

The Keyword Heading Word (KF) index allows you to retrieve every Keyword Heading assigned by authors that include a particular word.

Figure 1. Literature search.

Search History (75) View Saved

Contract

# ▲	Searches	Results	Type	Actions	Annotations
1	exp Occupational Exposure/	68109	Advanced	Display More Results	
2	exp Occupational Diseases/	139813	Advanced	Display More Results	
3	Lifting/	2801	Advanced	Display More Results	
4	Workload/	23365	Advanced	Display More Results	
5	Workplace/	27615	Advanced	Display More Results	
6	"axial loading*".ab,kf,ti.	1467	Advanced	Display More Results	
7	heavy lifting.ab,kf,ti.	478	Advanced	Display More Results	
8	"job site*".ab,kf,ti.	108	Advanced	Display More Results	
9	loadbearing.ab,kf,ti.	71	Advanced	Display More Results	
10	Weight-Bearing/	21598	Advanced	Display More Results	
11	load-bearing.ab,kf,ti.	4798	Advanced	Display More Results	
12	(occupational adj2 (disease* or exposure* or illness*)).ab,kf,ti.	38935	Advanced	Display More Results	
13	(physical* adj2 (work or job)).ab,kf,ti.	5039	Advanced	Display More Results	
14	strain.ab,kf,ti.	480889	Advanced	Display More Results	
15	(weight bearing or weightbearing).ab,kf,ti.	18793	Advanced	Display More Results	
16	carrying.ab,kf,ti.	138769	Advanced	Display More Results	
17	lifting.ab,kf,ti.	12645	Advanced	Display More Results	
18	"work load*".ab,kf,ti.	5017	Advanced	Display More Results	
19	"work location*".ab,kf,ti.	333	Advanced	Display More Results	
20	"work place*".ab,kf,ti.	2931	Advanced	Display More Results	
21	"work site*".ab,kf,ti.	1256	Advanced	Display More Results	
22	"workload*".ab,kf,ti.	33042	Advanced	Display More Results	
23	"workplace*".ab,kf,ti.	49872	Advanced	Display More Results	
24	"Worksite*".ab,kf,ti.	3734	Advanced	Display More Results	
25	"workabilit*".ab,kf,ti.	898	Advanced	Display More Results	
26	"work abilit*".ab,kf,ti.	2200	Advanced	Display More Results	
27	repetitive work.ab,kf,ti.	341	Advanced	Display More Results	
28	lifting.ab,kf,ti.	12645	Advanced	Display More Results	
29	squatting.ab,kf,ti.	1776	Advanced	Display More Results	
30	bending.ab,kf,ti.	39044	Advanced	Display More Results	
31	kneeling.ab,kf,ti.	975	Advanced	Display More Results	
32	exp occupational groups/ or farmers/	685499	Advanced	Display More Results	
33	exp Agricultural Workers' Diseases/	6658	Advanced		

34	Construction Industry/	1931	Advanced		
35	exp Work/	68777	Advanced	Display More Results	
36	exp Employment/	96034	Advanced	Display More Results	
37	Industry/	31934	Advanced	Display More Results	
38	exp Commerce/	67524	Advanced	Display More Results	
39	(agricultural worker* adj2 disease*).ab,kf,ti.	296	Advanced	Display More Results	
40	(building adj1 (trade* or industr*)).ab,kf,ti.	444	Advanced	Display More Results	
41	(construction adj1 (trade* or industr*)).ab,kf,ti.	1948	Advanced	Display More Results	
42	occupation.ab,kf,ti.	35985	Advanced	Display More Results	
43	"vocation*".ab,kf,ti.	13594	Advanced	Display More Results	
44	"employee*".ab,kf,ti.	52643	Advanced	Display More Results	
45	"occupational group*".ab,kf,ti.	3903	Advanced	Display More Results	
46	personnel.ab,kf,ti.	89031	Advanced	Display More Results	
47	(worker or workers).ab,kf,ti.	211429	Advanced	Display More Results	
48	employment.ab,kf,ti.	67058	Advanced	Display More Results	
49	(labor force* or labour force*).ab,kf,ti.	6196	Advanced	Display More Results	
50	(labor market or labour market).ab,kf,ti.	5305	Advanced		
51	occupational status.ab,kf,ti.	2544	Advanced		
52	underemployment.ab,kf,ti.	290	Advanced	Display More Results	
53	"business*".ab,kf,ti.	36607	Advanced	Display More Results	
54	"commerc*".ab,kf,ti.	306441	Advanced	Display More Results	
55	"vendor*".ab,kf,ti.	8404	Advanced	Display More Results	
56	cold temperature/ or hot temperature/	170168	Advanced	Display More Results	
57	Osteoarthritis, Hip/	9429	Advanced	Display More Results	
58	"coxarthr*".ab,kf,ti.	1861	Advanced	Display More Results	
59	(osteoarthr* adj2 (hip or hips)).ab,kf,ti.	3691	Advanced	Display More Results	
60	or/57-59	12006	Advanced	Display More Results	
61	Hip Injuries/ or Hip/	14388	Advanced	Display More Results	
62	(hip or hips or coxa*).ab,kf,ti.	166454	Advanced	Display More Results	
63	or/61-62	168952	Advanced	Display More Results	
64	Osteoarthritis/	41336	Advanced	Display More Results	
65	(osteoarthr* or degenerative arthr* or osteo-arthr*).ab,kf,ti.	88503	Advanced	Display More Results	
66	or/64-65	100427	Advanced	Display More Results	

67	and/63,66	16857	Advanced		
68	or/60,67	21302	Advanced		
69	Animals/	7121048	Advanced	Display More Results	
70	Humans/	20518164	Advanced	Display More Results	
71	69 not 70	4979354	Advanced	Display More Results	
72	or/1-56	2469214	Advanced	Display More Results	
73	and/68,72	1645	Advanced	Display More Results	
74	73 not 71	1594	Advanced	Display More Results	
75	limit 74 to (danish or english or norwegian or swedish)	1398	Advanced	Display More Results	

Appendix 2. Risk of bias tool.

Table 1.

Case-Control Study	Yes	No	Unclear
**Major domain 1 – study design and selection**			
**Were the cases recruited in an acceptable way?** Consider the following:<br>● Are the cases representative of a population, clearly defined and differentiated from controls?<br>● Was there an established reliable system for selecting all the cases?<br>● Were inclusion and exclusion criteria explicit and applied similarly to all eligible cases?			
**Were the controls selected in an acceptable way?** Consider the following:<br>● Are the controls representative of a population and clearly defined?<br>● Are the same inclusion and exclusion criteria for cases used to select controls (equally applied) and matched appropriately?<br>● Is it clearly established that controls are non-cases?			
**Is the participation rate satisfactory?** Consider the following:<br>● Are there large differences between the two groups?<br>● Is the participation rate low?			
**Major domain 2 – Exposure**			
**Was the exposure accurately measured to minimise bias?** Consider the following:<br>● Is the exposure clearly defined?<br>● Do measurements truly reflect what it is supposed to measure (have they been validated?).<br>● Is the method of assessment reliable?			
**Major domain 3 – Outcome**			
**Was the outcome accurately measured to minimise bias?** Consider the following:<br>● Is the outcome clearly defined?<br>● Do measurements truly reflect what it is supposed to measure (have they been validated?).<br>● Is the method of assessment reliable?			
**Major domain 4 – Non-participants**			
**Is comparison made between participants and non-participants?** Consider the following:<br>● Is similarities or differences established?			
**Major domain 5 – Analysis method**			
**Was the analysis method adequate?** Consider the following:<br>● Are the main potential confounders identified and taken into account in the analysis?<br>● Were adequate statistical models used to reduce bias?			
**Minor domain 1 – Funding**			
**Was the source of funding provided?** Consider the following:<br>● Was the study affected by sponsors?<br>● Did sponsoring organization participate in the analysis?			
**Minor domain 2 – Chronology**			
**Could chronology be established?** Consider the following:<br>● Was the timeframe sufficient to see an association between the exposure and outcome?			
**Minor domain 3 – Conflict of interest**			
**Was the study without any conflict of interest?** Consider the following:			

● Was the study affected by the authors affiliations or interests?			
**Cohort Study**	**Yes**	**No**	**Unclear**
**Major domain 1 – study design and selection**			
**Was the cohort recruited in an acceptable way?** Consider the following:<br>● Is it representative of a defined population and clearly specified?<br>● Are groups comparable in all respects other than the factor under investigation?<br>● Was everybody included who should have been?			
**Was the follow-up of subjects acceptable?** Consider the following:<br>● Conventionally, a 20% drop out rate is acceptable, but observational studies conducted over longer periods, a higher drop-out rate is to be expected.<br>● Were losses to follow-up taken into account in the analysis (sensitivity analysis, described etc.)?			
**Major domain 2 – Exposure**			
**Was the exposure accurately measured to minimise bias?** Consider the following:<br>● Is the exposure clearly defined?<br>● Do measurements truly reflect what it is supposed to measure (have they been validated?).<br>● Is the method of assessment reliable?<br>● Were all the subjects classified into exposure groups using the same procedure?			
**Major domain 3 – Outcome**			
**Was the outcome accurately measured to minimise bias?** Consider the following:<br>● Is the outcome clearly defined?<br>● Do measurements truly reflect what it is supposed to measure (have they been validated?).<br>● Is the method of assessment reliable?<br>● Were the measurement methods similar in the different groups?<br>● If blinding is not possible, is there some recognition that knowledge of exposure status could influence the assessment of the outcome?			
**Major domain 4 – Enrolment**			
**Was the outcome taken into account at enrolment?** Consider the following:<br>● Some participants might have the outcome at the time of enrolment. Is it assessed at baseline in the analysis?			
**Major domain 5 – Analysis method**			
**Was the analysis method adequate?** Consider the following:<br>● Are the main potential confounders identified and taken into account in the analysis?<br>● Were adequate statistical models used to reduce bias?			
**Minor domain 1 – Funding**			
**Was the source of funding provided?** Consider the following:<br>● Was the study affected by sponsors?<br>● Did sponsoring organization participate in the analysis?			
**Minor domain 2 – Chronology**			
**Could chronology be established?** Consider the following:<br>● Was the timeframe sufficient to see an association between the exposure and outcome?<br>● Was the follow-up long enough for the outcome to occur?			
**Minor domain 3 – Conflict of interest**			
**Was the study without any conflict of interest?** Consider the following:			

	Yes	No	Unclear
● Was the study affected by the authors affiliations or interests?			

**Cross-sectional studies**	**Yes**	**No**	**Unclear**
**Major domain 1 – study design and selection**			
**Were the subjects recruited in an acceptable way?**<br>Consider the following:<br>● Are subjects representative of a population, clearly defined and differentiated from controls?<br>● Was the method of selection of the subjects clearly described?<br>● Could the way the sample was obtained introduce bias?			
**Is the participation rate satisfactory?**<br>Consider the following:<br>● Was the sample size based on pre-study considerations of statistical power?<br>● Was a satisfactory response rate achieved or was the sample size justified?			
**Major domain 2 – Exposure**			
**Was the exposure accurately measured to minimise bias?**<br>Consider the following:<br>● Is the exposure clearly defined?<br>● Do measurements truly reflect what it is supposed to measure (have they been validated?).<br>● Is the method of assessment reliable?			
**Major domain 3 – Outcome**			
**Was the outcome accurately measured to minimise bias?**<br>Consider the following:<br>● Is the outcome clearly defined?<br>● Do measurements truly reflect what it is supposed to measure (have they been validated?).<br>● Is the method of assessment reliable?			
**Major domain 4 – Non-participants**			
**Is comparison made between participants and non-participants?**<br>Consider the following:<br>● Is similarities or differences established?			
**Major domain 5 – Analysis method**			
**Was the analysis method adequate?**<br>Consider the following:<br>● Are the main potential confounders identified and taken into account in the analysis?<br>● Were adequate statistical models used to reduce bias?			
**Minor domain 1 – Funding**			
**Was the source of funding provided?**<br>Consider the following:<br>● Was the study affected by sponsors?<br>● Did sponsoring organization participate in the analysis?			
**Minor domain 2 – Chronology**			
**Could chronology be established?**<br>Consider the following:<br>● Was the timeframe sufficient to see an association between the exposure and outcome?<br>● Was the follow-up long enough for the outcome to occur?			
**Minor domain 3 – Conflict of interest**			
**Was the study without any conflict of interest?**<br>Consider the following:<br>● Was the study affected by the authors affiliations or interests?			

Appendix 3. Grading the evidence of an association

The quality of evidence was evaluated in accordance with the GRADE guidelines, complying with guidelines stated by the Navigation Guide. Therefore, the evidence starts at moderate quality when evaluating observational studies.

The criteria and definitions stated in our instructions has been adopted from the Navigation Guide (Woodruff and Sutton 2014) (1) and Hulshof 2019 (2).

Table 2. Quality level - four levels of evidence.

**Quality level**	**Definition**
High ++++	We are confident that the true effect lies close to that of the estimate of the effect.
Moderate +++	We are moderately confident in the effect estimate: The true effect is likely to be close to the estimate of the effect, but there is a possibility that it is substantially different.
Low ++	Our confidence in the effect estimate is limited: The true effect may be substantially different from the estimate of the effect.
Very low +	We have very little confidence in the effect estimate: The true effect is likely to be substantially different from the estimate of effect.

Table 3. Evaluating the quality of evidence - down- and upgrading factors.

**Downgrading factors**	**Criteria for downgrading**<br>(0= no change; -1 (serious) or -2 (very serious) = downgrading 1 or 2 levels).
Risk of bias	Criteria:<br>Judicious considerations regarding each study's contribution towards the measure of association is warranted. One should be confident that there is a substantial risk of bias across most studies included and not downgrade based on an average count.
Inconsistency	Criteria:<br>Consider if<br>* Effect estimates of the included studies vary widely<br>* Confidence intervals show minimal or no overlap<br>* Heterogeneity (I-squared) is high<br><br>Inconsistency is important when it reduces the confidence in results. Even if inconsistency is large, estimates that, overall, points towards an increased risk would not be downgraded. For instance, if a protective or harmful effect cannot be determined, GRADE suggests downgrading.
Indirectness	Criteria:<br>* Do differences occur between populations of interest?<br>* Do differences occur between outcome measures of interest?<br>* Do differences occur between exposure measures of interest?<br>* Is differences substantial so that it decreases our confidence in comparisons or make difference in the outcome likely?
Imprecision	Criteria:<br>* Do studies have few participants or few events?<br>* Are the confidence intervals wide?<br>* Are few studies included in the pooled estimate?
Publication bias	Criteria:<br>* Consider Funnel plot and Egger's test<br>* Are there sufficient studies included to interpret the funnel plot accordingly?

	* In environmental research, consider the likelihood of publishing studies who finds no associations (underestimations might occur).
**Upgrading factors**	**Criteria for upgrading**<br>(0= no change; +1 or +2 = upgrading 1 or 2 levels).
Magnitude of effect	Criteria:<br>* Upgrade if a large magnitude of effect exists (GRADE defines it as OR/RR >2.0).<br>* But should be based on the knowledge of occupational health and expert judgement.
Dose response	Criteria:<br>*Does one or more studies show or test an exposure-response relation?
Residual confounding	Criteria:<br>* Upgrade if consideration of all plausible residual confounders, biases, or effect modifications would underestimate the effect when results show no effect.

Table 4. The GRADE assessment.

	Certainty assessment							
**No of studies**	**Study design**	**Risk of Bias**	**Inconsistency**	**Indirectness**	**Imprecision**	**Other considerations**	**Effect estimate (95% CI)**	**Certainty**
**Combined exposures**								
15	Observational studies	Not serious (0)	Not serious (0)	Not serious (0)	Not serious (0)	- Indication of publication bias (-1)<br>- Plausible confounding (0)<br>- Dose-response gradient (+1)<br>- Magnitude of effect (0)	OR 1.7 (1.4 to 2.0)	⊕⊕⊕◯ Moderate
**Lifting/carrying loads**								
11	Observational studies	Serious (-1)	Not serious (0)	Not serious (0)	Not serious (0)	- Indication of publication bias (-1)<br>- Plausible confounding (+1)<br>- Dose-response gradient (+1)<br>- Magnitude of effect (0)	OR 1.6 (1.3 to 1.9)	⊕⊕⊕◯ Moderate
**Standing**								
7	Observational studies	Very serious (-2)	Not serious (0)	Serious (-1)	Serious (-1)	Indication of publication bias (0)<br>- Plausible confounding (0)<br>- Dose-response gradient (+1)<br>- Magnitude of effect (0)	OR 1.3 (1.0 to 1.8)	⊕◯◯◯ Very low
**Walking**								
6	Observational studies	Very serious (-2)	Not serious (0)	Not serious (0)	Serious (-1)	Indication of publication bias (0)<br>- Plausible confounding (0)<br>- Dose-response gradient (+1)<br>- Magnitude of effect (0)	OR 1.3 (1.1 to 1.5)	⊕◯◯◯ Very low
**Climbing stairs**								
6	Observational studies	Serious (-1)	Not serious (0)	Not serious (0)	Serious (-1)	Indication of publication bias (0)<br>- Plausible confounding (0)<br>- Dose-response gradient (+1)<br>- Magnitude of effect (0)	OR 1.6 (1.1 to 2.2)	⊕⊕◯◯ Low
**Non-neutral postures**								
5	Observational studies	Serious (-1)	Not serious (0)	Not serious (0)	Serious (-1)	Indication of publication bias (0)<br>- Plausible confounding (0)<br>- Dose-response gradient (+1)<br>- Magnitude of effect (0)	OR 1.7 (1.4 to 2.1)	⊕⊕◯◯ Low
**Sitting**								
5	Observational studies	Serious (-1)	Not serious (0)	Not serious (0)	Not serious (0)	Indication of publication bias (0)<br>- Plausible confounding (0)<br>- Dose-response gradient (0)<br>- Magnitude of effect (0)	OR 0.6 (0.5 to 0.9)	⊕⊕◯◯ Low
**Kneeling**								
5	Observational studies	Very serious (-2)	Not serious (0)	Not serious (0)	Serious (-1)	Indication of publication bias (0)<br>- Plausible confounding (0)<br>- Dose-response gradient (0)<br>- Magnitude of effect (0)	OR 1.2 (0.9 to 1.5)	⊕◯◯◯ Very low
**Squatting**								
5	Observational studies	Very serious (-2)	Not serious (0)	Not serious (0)	Serious (-1)	Indication of publication bias (0)<br>- Plausible confounding (0)	OR 1.1 (0.9 to 1.4)	⊕◯◯◯ Very low

						- Dose-response gradient (0)<br>- Magnitude of effect (0)		
**Standing/walking**								
3	Observational studies	Not serious (0)	Not serious (0)	Not serious (0)	Serious (-1)	Indication of publication bias (0)<br>- Plausible confounding (0)<br>- Dose-response gradient (0)<br>- Magnitude of effect (0)	OR 1.1 (1.0 to 1.2)	⊕⊕◯◯ Low
**Kneeling/squatting**								
2	Observational studies	Not serious (0)	Not serious (0)	Not serious (0)	Serious (-1)	Indication of publication bias (0)<br>- Plausible confounding (0)<br>- Dose-response gradient (0)<br>- Magnitude of effect (0)	OR 1.3 (1.1 to 1.7)	⊕⊕◯◯ Low

Appendix 4. Excluded articles from full-text reading

Table 5. Excluded articles.

	**References**	**Reason for exclusion**
**1**	Ageberg E, Engstrom G, Gerhardsson De Verdier M, Rollof J, Roos EM, Lohmander LS. Effect of leisure time physical activity on severe knee or hip osteoarthritis leading to total joint replacement: A population-based prospective cohort study. BMC Musculoskelet Disord. 2012;13.	Other reasons (e.g., abstract, books).
**2**	Andersen S, Thygesen LC, Davidsen M, Helweg-Larsen K. Cumulative years in occupation and the risk of hip or knee osteoarthritis in men and women: a register-based follow-up study. Occup Environ Med. 2012;69(5):325-30.	Exposure criteria not fulfilled.
**3**	Andersen S, Thygesen LC, Davidsen M, Helweg-Larsen K. Cumulative years in occupation and the risk of hip or knee osteoarthritis in men and women: a register-based follow-up study. Occupational and Environmental Medicine. 2011;11.	Exposure criteria not fulfilled.
**4**	Andersson S, Nilsson B, Hessel T, Saraste M, Noren A, Stevens-Andersson A, et al. Degenerative joint disease in ballet dancers. Clinical Orthopaedics and Related Research. 1989;238:233-6.	Exposure criteria not fulfilled.
**5**	Axmacher B, Lindberg H. Coxarthrosis in farmers. Clin Orthop. 1993(287):82-6.	Exposure criteria not fulfilled.
**6**	Bullough P, Goodfellow J, O'Conner J. The relationship between degenerative changes and load-bearing in the human hip. J Bone Joint Surg Br. 1973;55(4):746-58.	Exposure criteria not fulfilled.
**7**	Cimmino MA, Parodi M. Risk factors for osteoarthritis. Seminars in Arthritis and Rheumatism. 2004;34(2 SUPPL.):29-34.	Other reasons (e.g., abstract, books).
**8**	Cimmino MA, Sarzi-Puttini P, Scarpa R, Caporali R, Parazzini F, Zaninelli A, et al. Clinical presentation of osteoarthritis in general practice: determinants of pain in Italian patients in the AMICA study. Semin Arthritis Rheum. 2005;35(1 Suppl 1):17-23.	Outcome criteria not fulfilled.
**9**	Cleveland RJ, Schwartz TA, Prizer LP, Randolph R, Schoster B, Renner JB, et al. Associations of educational attainment, occupation, and community poverty with hip osteoarthritis. Arthritis care & research. 2013;65(6):954-61.	Exposure criteria not fulfilled.
**10**	Cooper C, Campbell L, Byng P, Croft P, Coggon D. Occupational activity and the risk of hip osteoarthritis. Ann Rheum Dis. 1996;55(9):680-2.	Other reasons (e.g., abstract, books).
**11**	Cooper DJ, Scammell BE, Batt ME, Palmer D. Factors associated with pain and osteoarthritis at the hip and knee in Great Britain's Olympians: a cross-sectional study. British Journal of Sports Medicine. 2018;52(17):9.	Exposure criteria not fulfilled.
**12**	Croft P, Coggon D, Cruddas M, Cooper C. Osteoarthritis of the hip: An occupational disease in farmers. Br Med J. 1992;304(6837):1269-72.	Exposure criteria not fulfilled.
**13**	Cunningham RJ. Farmer's hip. Bmj. 1992;305(6845):118-9.	Other reasons (e.g., abstract, books).
**14**	Felson DT. Relation of obesity and of vocational and avocational risk factors to osteoarthritis. J Rheumatol. 2005;32(6):1133-5.	Other reasons (e.g., abstract, books).
**15**	Felson DT. Obesity and vocational and avocational overload of the joint as risk factors for osteoarthritis. J Rheumatol Suppl. 2004;70:2-5.	Other reasons (e.g., abstract, books).
**16**	Felson DT. Do occupation-related physical factors contribute to arthritis? Bailliere's Clinical Rheumatology. 1994;8(1):63-77.	Other reasons (e.g., abstract, books).
**17**	Franklin J, Ingvarsson T, Englund M, Lohmander S. Association between occupation and knee and hip replacement due to osteoarthritis: a case-control study. Arthritis Res Ther. 2010;12(3):R102.	Exposure criteria not fulfilled.

**18**	Fransen M, Agaliotis M, Bridgett L, MacKey MG. Hip and knee pain: Role of occupational factors. Best Practice and Research: Clinical Rheumatology. 2011;25(1):81-101.	Other reasons (e.g., abstract, books).
**19**	Goekoop RJ, Kloppenburg M, Kroon HM, Dirkse LEV, Huizinga TWJ, Westendorp RGJ, et al. Determinants of absence of osteoarthritis in old age. Scand J Rheumatol. 2011;40(1):68-73.	No measure of an association.
**20**	Gokhale CN, Simon SS, Hadaye RS, Lavangare SR. A cross-sectional study to screen community health volunteers for hip/knee-osteoarthritis and osteoporosis. J. 2019;8(6):2101-5.	Exposure criteria not fulfilled.
**21**	Grotle M, Hagen KB, Natvig B, Dahl FA, Kvien TK. Obesity and osteoarthritis in knee, hip and/or hand: An epidemiological study in the general population with 10 years follow-up. BMC Musculoskelet Disord. 2008;9	Exposure criteria not fulfilled.
**22**	Harris EC, Coggon D. HIP osteoarthritis and work. Best Practice and Research: Clinical Rheumatology. 2015;29(3):462-82.	Other reasons (e.g., abstract, books).
**23**	Hochberg MC. Risk factors for the development and progression of hip osteoarthritis. J Rheumatol. 2005;32(6):1135-6.	Other reasons (e.g., abstract, books).
**24**	Holmberg S, Stiernstrom EL, Thelin A, Svardsudd K. Musculoskeletal symptoms among farmers and non-farmers: a population-based study. Int J Occup Environ Health. 2002;8(4):339-45.	Exposure criteria not fulfilled.
**25**	Hubertsson J, Turkiewicz A, Petersson I, Englund M. OCCUPATION AND RISK OF SICK LEAVE AND DISABILITY PENSION DUE TO KNEE AND HIP OSTEOARTHRITIS IN MEN AND WOMEN. Ann Rheum Dis. 2015;74:552-3.	Other reasons (e.g., abstract, books).
**26**	Hubertsson J, Turkiewicz A, Petersson IF, Englund M. Understanding Occupation, Sick Leave, and Disability Pension Due to Knee and Hip Osteoarthritis From a Sex Perspective. Arthritis care & research. 2017;69(2):226-33.	Outcome criteria not fulfilled.
**27**	Iosifidis MI, Tsarouhas A, Fylaktou A. Lower limb clinical and radiographic osteoarthritis in former elite male athletes. Knee Surg Sports Traumatol Arthrosc. 2015;23(9):2528-35.	Outcome criteria not fulfilled.
**28**	Jacobsen S, Sonne-Holm S. Hip dysplasia: A significant risk factor for the development of hip osteoarthritis. A cross-sectional survey. Rheumatology (Oxford). 2005;44(2):211-8.	Outcome criteria not fulfilled.
**29**	Jacobsen S, Sonne-Holm S, Soballe K, Gebuhr P, Lund B. Joint space width in dysplasia of the hip. Journal of Bone and Joint Surgery - Series B. 2005;87(4):471-7.	Exposure criteria not fulfilled.
**30**	Jarvholm B, From C, Lewold S, Malchau H, Vingard E. Incidence of surgically treated osteoarthritis in the hip and knee in male construction workers. Occupational and Environmental Medicine. 2008;65(4):275-8.	Exposure criteria not fulfilled.
**31**	Jarvholm B, Lundstrom R, Malchau H, Rehn B, Vingard E. Osteoarthritis in the hip and whole-body vibration in heavy vehicles. Int Arch Occup Environ Health. 2004;77(6):424-6.	Exposure criteria not fulfilled.
**32**	Johansson H, Hongslo Vala C, Oden A, Lorentzon M, McCloskey E, Kanis JA, et al. Low risk for hip fracture and high risk for hip arthroplasty due to osteoarthritis among Swedish farmers. Osteoporos Int. 2018;29(3):741-9.	Exposure criteria not fulfilled.
**33**	Kettunen JA, Kujala UM, Kaprio J, Koskenvuo M, Sarna S. Lower-limb function among former elite male athletes. Am J Sports Med. 2001;29(1):2-8.	Exposure criteria not fulfilled.
**34**	Kirkhorn S, Greenlee RT, Reeser JC. The Epidemiology of Agriculture-related Osteoarthritis and its Impact on Occupational Disability. Wis Med J. 2003;102(7):38-44.	Other reasons (e.g., abstract, books).
**35**	Kujala UM, Kaprio J, Sarna S. Osteoarthritis of weight bearing joints of low limbs in former elite male athletes. Br Med J. 1994;308(6923):231-4.	No measure of an association.
**36**	L'Hermette M, Polle G, Tourny-Chollet C, Dujardin F. Hip passive range of motion and frequency of radiographic hip osteoarthritis in former elite handball players. British Journal of Sports Medicine. 2006;40(1):45-9.	Exposure criteria not fulfilled.
**37**	Lanyon P, Muir K, Doherty S, Doherty M. Assessment of a genetic contribution to osteoarthritis of the hip: sibling study. Br Med J. 2000;321(7270):1179-83.	Exposure criteria not fulfilled.
**38**	Lee CG. Work-related musculoskeletal disorders in Korean farmers. J Korean Med Assoc. 2012;55(11):1054-62.	Other reasons (e.g., abstract, books).
**39**	Lindberg H. [Hereditary and mechanical factors behind arthrosis of the hip and the knee]. Lakartidningen. 1990;87(51-52):4403-4, 6.	Other reasons (e.g., abstract, books).

**40**	Lindberg H, Danielsson LG. The relation between labor and coxarthrosis. Clinical Orthopaedics and Related Research. 1984;191:159-61.	Exposure criteria not fulfilled.
**41**	Lindberg H, Roos H, Gardsell P. Prevalence of coxarthrosis in former soccer players: 286 players compared with matched controls. Acta Orthop Scand. 1993;64(2):165-7.	Exposure criteria not fulfilled.
**42**	Mahomed NN. Does occupational lifting cause hip osteoarthritis? J Rheumatol. 2000;27(2):292-3.	Other reasons (e.g., abstract, books).
**43**	Nicholls RA. Intra-articular disorders of the hip in athletes. Phys Ther Sport. 2004;5(1):17-25.	Other reasons (e.g., abstract, books).
**44**	Plotnikoff R, Karunamuni N, Lytvyak E, Penfold C, Schopflocher D, Imayama I, et al. Osteoarthritis prevalence and modifiable factors: a population study. BMC Public Health. 2015;15:10.	Exposure criteria not fulfilled.
**45**	Pope DP, Hunt IM, Birrell FN, Silman AJ, Macfarlane GJ. Hip pain onset in relation to cumulative workplace and leisure time mechanical load: A population based case-control study. Ann Rheum Dis. 2003;62(4):322-6.	Outcome criteria not fulfilled.
**46**	Roos H. Are there long-term sequelae from soccer? Clin Sports Med. 1998;17(4):819-31.	Other reasons (e.g., abstract, books).
**47**	Rossignol M. Primary osteoarthritis and occupation in the Quebec National Health and Social Survey. Occupational and Environmental Medicine. 2004;61(9):729-35.	Outcome criteria not fulfilled.
**48**	Rossignol M, Leclerc A, Allaert FA, Rozenberg S, Valat JP, Avouac B, et al. Primary osteoarthritis of hip, knee, and hand in relation to occupational exposure. Occupational and Environmental Medicine. 2005;62(11):772-7.	Exposure criteria not fulfilled.
**49**	Rossignol M, Leclerc A, Hilliquin P, Allaert FA, Rozenberg S, Valat J, et al. Primary osteoarthritis and occupations: a national cross sectional survey of 10 412 symptomatic patients. Occup Environ Med. 2003;60(11):882-6.	Exposure criteria not fulfilled.
**50**	Sandmark H. Musculoskeletal dysfunction in physical education teachers. Occupational and Environmental Medicine. 2000;57(10):673-7.	Exposure criteria not fulfilled.
**51**	Schmitt H, Brocai DRC, Lukoschek M. High prevalence of hip arthrosis in former elite javelin throwers and high jumpers: 41 Athletes examined more than 10 years after retirement from competitive sports. Acta Orthop Scand. 2004;75(1):34-9.	Exposure criteria not fulfilled.
**52**	Schneider S, Schmitt G, Mau H, Schmitt H, Sabo D, Richter W. Prevalence and correlates of osteoarthritis in Germany. Representative data from the First National Health Survey. Orthopade. 2005;34(8):782-90.	Other reasons (e.g., abstract, books).
**53**	Seok H, Choi SJ, Yoon JH, Song GG, Won JU, Kim JH, et al. The association between osteoarthritis and occupational clusters in the Korean population: A nationwide study. PLoS ONE. 2017;12(1) (no pagination).	Exposure criteria not fulfilled.
**54**	Spector TD, Harris PA, Hart DJ, Cicuttini FM, Nandra D, Etherington J, et al. Risk of osteoarthritis associated with long-term weight-bearing sports: a radiologic survey of the hips and knees in female ex-athletes and population controls. Arthritis Rheum. 1996;39(6):988-95.	Exposure criteria not fulfilled.
**55**	Steen Rubak T, Wulff Svendsen S, Frost P. 0140Risk of total hip replacement in relationship to cumulative exposures in the work environment. Occup Environ Med. 2014;71:A17-A.	Other reasons (e.g., abstract, books).
**56**	Tateuchi H, Koyama Y, Akiyama H, Goto K, So K, Kuroda Y, et al. Daily cumulative hip moment is associated with radiographic progression of secondary hip osteoarthritis. Osteoarthritis and Cartilage. 2017;25(8):1291-8.	Exposure criteria not fulfilled.
**57**	Taylor-Gjevre RM, Trask C, King N, Koehncke N, Saskatchewan Farm Injury Cohort Study T. Prevalence and occupational impact of arthritis in Saskatchewan farmers. J. 2015;20(2):205-16.	Study design criteria not fulfilled.
**58**	Teichtahl AJ, Smith S, Wang YY, Wluka A, O'Sullivan R, Giles GG, et al. Occupational risk factors for hip osteoarthritis are associated with early hip structural abnormalities: a 3.0 T magnetic resonance imaging study of community-based adults. Arthritis Res Ther. 2015;17:8.	Outcome criteria not fulfilled.
**59**	Thelin A. Hip joint arthrosis: an occupational disorder among farmers. Am J Ind Med. 1990;18(3):339-43.	Exposure criteria not fulfilled.

**60**	Thelin A. Arthritis of the hip joint is a common complaint amongst farmers. [Swedish]. Lakartidningen. 1985;82(46):3994-9.	Exposure criteria not fulfilled.
**61**	Thelin A, Holmberg S. Hip osteoarthritis in a rural male population: A prospective population-based register study. Am J Ind Med. 2007;50(8):604-7.	Exposure criteria not fulfilled.
**62**	Thelin A, Vingard E, Holmberg S. Osteoarthritis of the hip joint and farm work. Am J Ind Med. 2004;45(2):202-9.	Exposure criteria not fulfilled.
**63**	Tuchsen F, Hannerz H, Burr H, Lund T, Krause N. Risk factors predicting hip pain in a 5-year prospective cohort study. Scandinavian Journal of Work, Environment and Health. 2003;29(1):35-9.	Outcome criteria not fulfilled.
**64**	Tuchsen F, Hannerz H, Jensen MV, Krause N. Socioeconomic status, occupation, and risk of hospitalisation due to coxarthrosis in Denmark 1981-99. Ann Rheum Dis. 2003;62(11):1100-5.	Exposure criteria not fulfilled.
**65**	van Dijk CN, Lim LS, Poortman A, Strubbe EH, Marti RK. Degenerative joint disease in female ballet dancers. Am J Sports Med. 1995;23(3):295-300.	No measure of an association.
**66**	Vingard E, Alfredsson L, Goldie I, Hogstedt C. Sports and osteoarthrosis of the hip. An epidemiologic study. Am J Sports Med. 1993;21(2):195-200.	No measure of an association.
**67**	Vingard E, Alfredsson L, Goldie I, Hogstedt C. Occupation and osteoarthrosis of the hip and knee: A register-based cohort study. Int J Epidemiol. 1991;20(4):1025-31.	Exposure criteria not fulfilled.
**68**	Vingard E, Alfredsson L, Hogstedt C, Goldie I. [Increased risk of arthrosis of the knee and hip among workers with heavy weight on the legs]. Lakartidningen. 1990;87(51-52):4413-6.	Other reasons (e.g., abstract, books).
**69**	Vingard E, Alfredsson L, Malchau H. Osteoarthrosis of the hip in women and its relationship to physical load from sports activities. Am J Sports Med. 1998;26(1):78-82.	Exposure criteria not fulfilled.
**70**	Vingard E, Alfredsson L, Malchau H. Lifestyle factors and hip arthrosis. A case referent study of body mass index, smoking and hormone therapy in 503 Swedish women. Acta Orthop Scand. 1997;68(3):216-20.	Exposure criteria not fulfilled.
**71**	Vingard E, Sandmark H, Alfredsson L. Musculoskeletal disorders in former athletes. A cohort study in 114 track and field champions. Acta Orthop Scand. 1995;66(3):289-91.	Outcome criteria not fulfilled.
**72**	Yesil H, Hepguler S, Ozturk C, Yesil M, Capaci K. Risk Factors of Symptomatic Knee, Hand and Hip Osteoarthritis in a Suburban Area of Izmir City. Turk Fiz Tip Rehabil Derg. 2014;60(2):126-33.	Exposure criteria not fulfilled.
**73**	Zetterberg C, Hansson T. [Arthrosis of the hip and knee. Heredity, sports and overweight are usually more hazardous than work]. Lakartidningen. 1995;92(22):2307-10.	Other reasons (e.g., abstract, books).

Appendix 5. Characteristics of the included 24 studies.

Table 6. Description of each included article.

			Outcome		Exposure	
**Author**	**Design**	**Study population**	**Definition**	**Assessment**	**Definition**	**Assessment**
**Allen, 2010 (43)**	Cross-sectional.	The sample consisted of 2506 individuals from the state of North Carolina, USA, presented with symptomatic hip osteoarthritis. Participants eligible for the analysis were enrolled from baseline (1991-1997 and from the first follow-up (1999-2004). The mean age of the total sample was 63.6 (SD=10.5).	Hip osteoarthritis was examined by radiographic criteria, irrespective of symptoms. Supine anteroposterior pelvis radiographs were obtained on all men and women 50 years of age and older (due to reproductive issues among women). All radiographs were read according to the Kellgren-Lawrence score, and interrater and intra-rater reliability was calculated. Symptomatic hip OA was assessed by asking "On most days, do you have pain, aching, or stiffness in right/left hip?". Hip osteoarthritis was divided into a radiographic group (presence of radiographic osteoarthritis and symptoms in the same joint) and a symptomatic group.	Radiographic and symptomatic.	Mechanical exposure on the longest job participants held was assessed as walking, lifting/carrying/moving objects weighing > 10 pounds, sitting, standing, bending/twisting/reaching, squatting, climbing stairs, crawling on knees, crouching or kneeling, and heavy work while standing.	Questionnaire.
**Coggon, 1998 (44)**	Case-control.	868 men and women were identified. 611 were included (210 men and 401 women) as cases. Cases comprised residents of two English cities, Portsmouth and North Staffordshire (1993-1995), on a waiting list for total hip replacement for osteoarthritis over an 18-month period aged 45 years and over. For each case, a control of the same sex and age (within 4 years) was selected from the list of the same general practice. The mean age of all participants was 70 (SD=9).	A register was established in each district whereby the orthopaedic surgeons recorded all men and women who were placed on the waiting list for primary total hip arthroplasty. The pelvic radiographs of each case were evaluated for the presence of osteoarthritis using: measurements of minimal joint space, an overall Kellgren-Lawrence score, and the anatomical pattern of joint involvement (superolateral or medical/concentric).	Radiographic.	Mechanical exposures on jobs held longer than one year since leaving school was assessed as lifting weights of at least 10 kg, at least 25 kg, and at least 50 kg for more than 10 times in an average working week. Furthermore, information was gathered on sitting, standing, kneeling, squatting, driving, walking, and climbing.	Questionnaire.
**Croft, 1992 (45)**	Case-control.	353 cases and 434 referents (all men aged 60-75 years) who had undergone intravenous urography were identified from x-ray registers. Cases and referents were ascertained through the radiology departments of the North Staffordshire and Shrewsbury hospitals, England (1982-1987). A total of 245 cases and 294 referents were included.	Cases were defined as those who had a total hip replacement for osteoarthritis and those in whom the shortest distance between the femoral head and the acetabular roof was $\leq$2.5 mm in at least one hip.<br>Cases were divided into a subset of "severe cases" with a hip replacement or a minimal joint space of $\leq$1.5 mm. Referents had a joint space >3.5 mm in both hips and showed no other radiographic evidence of osteoarthritis.	Radiographic.	Mechanical exposures were defined as how long a participant had been exposed to each of the following activities (years):<br>sitting, standing, bending, kneeling, squatting, walking, running, climbing, lifting or moving weights (>25.4 kg), and driving.	Interview.
**Cvijetic, 1999 (46)**	Cross-sectional.	678 participants were randomly selected from city records in Zagreb, Croatia (1981-1983). 590 participants were enrolled consisting 292	Hip joints were examined for pain, stiffness and range of motion. Radiographs of the right hip were taken from all participants and the degree of	Radiographic.	Subjects were divided in 4 groups according to physical demands pertinent to their occupation.:	Interview.

		women and 298 men. Mean age for women was 62.5 (SD=10.3) and 63.5 (SD=11) for men.	osteoarthritis in the individual joint was graded on a five-point scale according to the standard of Kellgren and co-workers. Grades 2, 3, and 4 were considered definite signs of osteoarthritis.		Category 1 – mostly sedentary jobs, >80% of time in the sitting position<br>Category 2 – >80% of time in the standing position;<br>Category 3 – >80% of time in non-sitting positions (frequent walking and standing, but with low physical strain, lifting and carrying light objects of up to 5 kg).<br>Category 4 – jobs with high physical strain, >80% of time in non-sitting position (frequent walking and standing, lifting and carrying heavy objects of over 5 kg).	
**Flugsrud, 2002 (47)**	Cohort.	The study invited 56,818 persons, and 52,143 responded. For the analysis, 50,034 persons were eligible. All participants were sampled from 3 Norwegian counties and underwent a cardiovascular screening performed by nurses (from 1977-1983). The occurrence of the first total hip replacement for primary osteoarthritis was recorded during follow-up (1989-1998). Median age at the start of follow-up was 54.9 (range 46-67 years).	All total hip replacements and hip implant revisions done in Norwegian hospitals were collected from the Norwegian Arthroplasty Register. For every total hip replacement (and hip implant revision), a form was completed to record previous hip surgery in either hip, the indication for surgery, the implants used, and other parameters related to the operation.	Register data on total hip replacements.	Mechanical exposures were assessed as physical activity at work divided into:<br>- Sedentary (mostly sedentary work).<br>- Moderate (work leading to much walking).<br>- Intermediate (work leading to much walking and lifting).<br>- Intensive (Heavy manual labour).	Questionnaire.
**Heliovaara, 1993 (48)**	Cross-sectional.	The study sample consisted of 3637 men and 4363 women drawn from a population register in Finland, aged ≥30. 7217 persons participated in a screening phase and a diagnostic phase from 1977 to 1980.	Participants with a least one symptom from the screening phase were asked to participate in the diagnostic phase. Coxarthrosis was diagnosed by a physician if there was either a convincingly documented history or definite findings in the physical status of one or both hips from:<br>- Physical examination.<br>- Medical history.<br>- Prescriptions, health records, radiographs, and physician's certificates.	Clinical diagnosis.	Mechanical exposures included the participant's present and/or previous occupations involving exposure to lifting or carrying heavy objects, stooped, twisted or otherwise awkward work posture, vibration of the whole body or use of vibrating equipment, a continuously repeated series of movements, and working speed determined by a machine.<br>The total number of these factors was designated "the sum index of physical stress at work".	Questionnaire.
**Jacobsson, 1987 (49)**	Case-control.	Male participants from Skövde in Sweden were recruited consisting of 85 with coxarthrosis and 262 patients with intravenous urogram for prostatic hyperplasia.	Urography films were examined and graded into three groups:<br>1. Normal joint space.<br>2. Reduced joint space greater than 3 mm<br>3. A joint space of less than 3 mm.<br><br>The fourth group consisted of patients on waiting list for operation.<br><br>Cases consisted of participants from group 3 and 4 whereas controls consisted of participants from group 1 and 2.	Radiographic.	Participants was asked if they had been subject to heavy labour, particularly to farming, forestry, industrial work or heavy lifting, or too much walking, standing, or tractor driving.	Questionnaire.
**Juhakoski, 2009 (50)**	Cohort.	Baseline data was collected in 1978-1980 from a representative sample of 8000 people from Finland. A total of 7217 subjects participated in the screening phase.	Specially trained physicians carried out the clinical examinations and diagnosed hip osteoarthritis according to a standardised written	Clinical examination.	Mechanical exposure was defined as physical workload (6 categories):<br>- Light sedentary work (sitting involving only in light manual work).	Questionnaire.

		Subjects were asked to attend a clinical examination if they had experienced any difficulties in walking due to hip pain or were found to have difficulty in performing the function tests. In 2000-01, 1286 were re-invited and re-examined. A total of 909 participated. At follow-up, the mean age was 63 years (SD=8). The study comprised 840 subjects (371 men and 469 women).	protocol (disease history, symptoms, and clinical findings according to standard criteria).		- Other sedentary work (sedentary work but involves handling fairly heavy objects).<br>- Physically light standing work (mostly standing work without cumbersome movements without carrying heavy burdens).<br>- Fairly light or medium-heavy work (great deal of moving and a fair amount of stooping down or carrying light objects).<br>- Heavy manual work (standing involving much lifting of light objects or lifting and carrying heavy objects).<br>- Very heavy manual work (mostly continuous heavy movements).	
**Kaila-Kangas, 2011 (51)**	Cross-sectional.	A nationally representative sample of 8028 subjects was sampled from different clusters in Finland in 2010. The study comprised 6556 subjects who participated in both a clinical examination and home interview (3110 men and 3446 women). The mean age of the study participants was 51 years (SD=14) for the men and 53 (SD=14) for the women.	The diagnosis of hip OA was made from disease history, symptoms and clinical findings according to predefined diagnostic criteria. Radiographs was taken of a sub-sample to rate the agreement.	Clinical examination.	Only lifting was examined and was assessed with the question: "Did your work involve the manual handling of heavy objects, such as lifting, carrying or pushing loads over 20 kg on average of at least 10 times per working day?"	Interview.
**Kontio, 2020 (52)**	Cohort.	A national representative sample from Finland, 2010, consisting of 5254 participants aged between 30 to 59 years. Participants were followed from baseline until the first hospitalisation of hip osteoarthritis, death, or end of follow-up in December 2015.<br>4642 were eligible for the present study (2247 men and 2395 women).	The diagnoses were classified according to the International Classification of Diseases (ICD). The diagnoses of primary interest were hip OA (ICD-8: 713.00; ICD-9: 175.15; and ICD-10: M16).	Register.	The following mechanical exposures were assessed:<br>- Physically heavy work.<br>- Manual handling of heavy loads (lifting ≥ 20 kg >10 times a day).<br>- Kneeling and squatting for 1 hour per day.<br>- Standing or walking for ≥5 hour per day.<br>- Cumulative exposure for each mechanical exposure.	Interview.
**Lau, 2000 (53)**	Case-control.	Participants with hip osteoarthritis were recruited from the orthopaedic units of seven regional hospitals in Hong Kong, comprising 30 men and 108 women. Controls were recruited in eight government general practice clinics located in the same region as those for the study, matched on sex and age. Controls comprised 90 men and 324 women.	Medical records of all hip osteoarthritis patients were reviewed. Orthopaedic surgeons managing the patients graded the patients according to Kellgren and Lawrence scale using radiographs of the hip. Only patients with grade 3 or 4 OA were included.	Radiographic.	For each job held for a year or more, following activities were obtained on mechanical exposures:<br>- Walking for ≥2 hours/day.<br>- Squatting for ≥1 hour/day.<br>- Kneeling for ≥1 hours/day.<br>- Climbing ≥15 flights of stairs/day.<br>- Driving for ≥4 hours/day.<br>- Lifting of loads weighing ≥10 kg for ≥1–10 times.<br>- Use of vibration tools for ≥1 hours/day.	Interview.
**Lau, 2007 (54)**	Case-control.	Participants with hip osteoarthritis was recruited from the orthopaedic units of seven regional hospitals in Hong Kong, comprising 30 men and 108 women. Controls were recruited in eight government general practice clinics located in the same region as those for	Medical records of all hip osteoarthritis patients were reviewed. Orthopaedic surgeons managing the patients graded the patients according to Kellgren and Lawrence scale using radiographs of the hip. Only patients with grade 3 or 4 OA were included.	Radiographic.	For each job held for a year or more, following activities were obtained on mechanical exposures:<br>- Walking for ≥2 hours/day.<br>- Squatting for ≥1 hour/day.<br>- Kneeling for ≥1 hour/da.,<br>- Climbing ≥15 flights of stairs/day.<br>- Driving for ≥4 hours/day.	Interview.

		the study, matched on sex and age. Controls comprised 90 men and 324 women.			- Lifting of loads (weighing >10 kg and >50 kg) for 1–10 times or >10 times each week.<br>- Use of vibration tools for ≥1. hours/day.	
**Olsen, 1994 (55)**	Case-control.	The study comprised all Swedish men between the age of 50 to 70 years living in areas around four large hospitals in Stockholm. Data was collected from 1984 to 1988. Cases were men (n=239) who received first time prosthesis as a result of idiopathic osteoarthrosis. Controls (n=302) were randomly selected from the study base.	The cases were those men in the study population who received a first-time prosthesis of the hip joint as a result of idiopathic osteoarthrosis. The orthopaedic clinics involved were contacted each week; they delivered the names and addresses of new patients.	Registers.	Mechanical exposures:<br>- Cumulative number of hours exposed to dynamic workloads.<br>- Cumulative number of hours working in a twisted locked position.<br>- Cumulative number of tons lifted.<br>- Total number of jumps.<br>Cumulative exposures were calculated from the start of the occupational carrier up until the age of 49 years.<br>Three exposure groups were defined according to the loads in the reference group.	Interview.
**Ratzlaff, 2011 (56)**	Cohort.	The source population consisted of community-dwelling members of the Canadian Association of Retired Persons. Over 100,000 persons were approached either through e-mail or newsletters, and 4258 completed the baseline survey in 2005. A follow-up survey was sent between 2006 and 2007. In total, 2918 participants were enrolled with a mean age of 61.6 (SD=7.3).	Subjects were asked to report health-professional-diagnosed hip osteoarthritis on at least one of the two follow-up surveys. The questionnaire used pain diagrams and items specific to osteoarthritis and specifically informed subjects that osteoarthritis was distinct from other musculoskeletal diseases.	Self-report.	Mechanical exposures were analysed as cumulative peak force index estimating time spend in specific occupational activities (hours), body weight, and the peak hip joint force for each activity (%body weight), and divided into quintiles.<br>It was based on the following questions:<br>- Duration of participation in each occupation.<br>- Type of employment and length of the average season.<br>- Hours per week working.<br>- Time spend walking, standing, lifting, carrying, using heavy tools, squatting etc.	Questionnaire.
**Rijs, 2014(57)**	Cross-sectional.	Participants were enrolled in a continuing population-based cohort study (Longitudinal Aging Study Amsterdam) and were a random sample of 55- to 85-year-olds drawn from population registries in 11 municipalities in 3 regions of the Netherlands, consisting of 3107 participants. After exclusions, an analytic sample of 1676 participants were eligible.	Hip osteoarthritis was defined by an algorithm using self-report and general practitioner data and categorised as no, possible, or definite osteoarthritis. Finally, osteoarthritis categories were collapsed as no and possible/definite osteoarthritis.	Self-report and general practitioner data.	Information on mechanical exposures was gathered from a general population job-exposure matrix. Occupational classes were classified as having a low, moderate, or high probability of exposure based on self-reported levels of work exposure gathered from the Netherlands Work Condition Survey. Classifications (cut-offs) were chosen depending on the proportion of reporting to physical demands. The following categories were used:<br>- Use of force (lifting, pushing, pulling, carrying, or force with tools).<br>- Perform work in an uncomfortable position.<br>- Repetitive movements.	Job-exposure matrix.
**Riyazi, 2008 (58)**	Case-control.	191 sibling pairs (382 cases) from the Netherlands with familial osteoarthritis at multiple sites were included in the study. 345 controls were recruited by random sampling of the population using random-digit-dialling and	Radiograph of the hips (Posterior-Anterior, weight bearing) were scored by an experienced musculoskeletal radiologist using the Kellgren-Lawrence method. A score of >2 depicts osteoarthritis.	Radiographic.	Job title classification of physically demanding work was based on a revised classification scheme of physical and mental work demands into different categories according to an expert judgment of job titles used in the Netherlands.	Revised classification scheme based on expert assessments.

		returned the questionnaire. The mean age of cases was 60 years, and the mean age of controls was 57 years. Cases consisted of 82 % women whereas controls consisted of 64 % women.			Mechanical exposures were assessed as physically demanding work characterised by lifting of heavy objects, handling of heavy tools, stooping, frequently in combination with standing or walking.	
**Roach, 1994 (59)**	Case-control.	Male patients attending outpatient clinics at a large Department of Veterans Affairs hospital in the metropolitan of Chicago were drawn as both cases and controls. Cases were identified from the computerised radiology database of all patients receiving an outpatient radiograph of hip pain or following a total hip arthroplasty from 1989 to June 1990.<br>Controls were screened for possible hip OA using intravenous pyelogram films and selected from the population of patients receiving this screening.<br>99 cases and 233 controls were used in the analysis, with a mean age of 68.2 (SD=6.4) for cases and 67.7 (SD=7.1) for controls.	Computerised radiology reports, information from the Veteran's Affair patient database, and medical records were used to determine whether subject meet the American College of Rheumatology criteria for hip OA.	Radiographic and register data.	Mechanical exposures were assessed as number of years exposed to:<br>1. Light work standing.<br>2. Work sitting.<br>3. Heavy work standing.<br>4. Work kneeling or crouching.<br>5. Work walking.<br><br>Mechanical exposures were classified as either heavy or light work:<br>- Heavy = work standing, work walking, and work kneeling or crouching.	Questionnaire.
**Rubak, 2013 (60)**	Cohort.	All Danish men and women born between 1925 and 1964 with at least 10 years of full-time employment between 1964 and 2006, was eligible for this cohort study. Information was obtained in 2007 from the Danish Civil Registration System.<br>In total, 899549 women and 1010944 men were included. At the start of the individual follow-up, mean age was 48.2 for men and 49.1 years for women.	Total hip replacement due to osteoarthritis was assessed using ICD-codes (M16.0, M16.1, or M16.9) and surgical procedure codes (Nordic Medico-statistical Committee Classification of Surgical Procedures codes KNFB20, KNFB30, KNFB40, or KNFB99) gathered from Danish National Patient Register.	Register data.	An industry exposure matrix was developed rating the overall physical workload to the hip on a 3-point scale (0= minimal load, 1=moderate load, 2=high load). Exposures that were taken into consideration:<br>- Total load lifted per day<br>- Frequency of lifting burdens weighing ≥20 kg<br>- Whole-body vibration<br>- Standing/walking the majority of the working day.<br><br>For each individual, point-years, a cumulative estimate of physical workload, were calculated as the number of employment years (adjusted to full-time employment) in a specific industry times the corresponding score of physical workloads from the industry exposure matrix and summarised across all registered employments.	Industry-exposure matrix.
**Rubak, 2014 (61)**	Nested case-control in cohort.	All Danish men and women born between 1935 and 1964 with at least 10 years of full-time employment before January 1, 2006, were eligible. Cases with first-time total hip replacement due to primary osteoarthritis were identified in 2005 and 2006. For each case, 2 controls matched on age and sex were sampled. Of 7445 unique persons, 5495 responded. Altogether, 1776 case-control sets (862 sets of women and 915 sets of men) were	Total hip replacement was due to osteoarthritis was assessed using ICD-codes (M16.0, M16.1, or M16.9) and surgical procedure codes (Nordic Medico-statistical Committee Classification of Surgical Procedures codes KNFB20, KNFB30, KNFB40, or KNFB99) gathered from Danish National Patient Register.	Register data.	Mechanical exposures were assessed by combining self-reported job titles with a job-exposure matrix (The lower body matrix). The matrix provides estimates of the total load lifted per day and daily frequency of lifting loads weighing ≥20 kg as well as hours of exposure to standing/walking, sitting, kneeling/squatting, and whole-body vibrations during an 8-hour working day.<br><br>Cumulative mechanical exposure was calculated as:<br>- 1 ton-year standardised as lifting 1 ton per day for	Job-exposure matrix.

		available for the analysis with ages ranging from 41 to 69 years.			1 year, and 1 frequent heavy lifting–year as lifting loads weighing >20 kg >10 times per day for 1 year.	
**Solovieva, 2018 (62)**	Cohort.	Random sample of the Finish population. The cohort consisted of 574,617 men and 561,037 women living in Finland on December 31, 2004, aged 30 to 60 years who had gainful jobs on January 1, 2005. Data were obtained from the National register of the Finnish Centre for Pensions.	Information on osteoarthritis was obtained from the Finnish Centre for Pensions register. It provides information on all disability retirement events classified according to ICD classification. The outcome was all full-time disability retirement (temporary or permanent) as result of hip osteoarthritis (ICD-10 code = M16) from 2005 until 2013.	Register data.	A sex-specific job-exposure matrix was used to gather information on:<br>- Heavy physical work.<br>- Kneeling or squatting.<br>- Manual handling of heavy loads.<br>- Sitting.<br>- Standing or moving at work.	Job-exposure matrix.
**Thelin, 1997 (63)**	Case-control.	All radiological examinations of hip or pelvis joints performed between 1986 to 1988 at three different hospitals in Skaraborg, Sweden were re-evaluated in 1989. Controls were selected using a local population register. A total of 216 male cases answered the questionnaire and 479 male controls matched on age, and place of residence were selected from a local population register. All participants were under 70 years of age.	All hip joints were assessed with radiographic and a joint space of <3 mm was classified as coxarthrosis, unless special information showed that it was a question of a congenital condition.	Radiographic.	Mechanical exposure was assessed as heavy physical work at young age (before 16 years of age). This was defined as full time working with what they themselves regarded as heavy physical work for a period longer than half a year.	Questionnaire.
**Vingård, 1991 (64)**	Case-control.	The study population comprised men aged between 50 and 70 years, living in referral areas of four large hospitals in Stockholm, Sweden. Information was collected from 1984 until 1988. 233 cases were those men who received a first-time prosthesis of the hip joint as a result of idiopathic osteoarthrosis. 302 controls were randomly selected from the study population.	Information on first time hip prosthesis was gathered from contacting the orthopaedic clinics that were involved in the study. They delivered names and addresses of the patients.	First time hip prosthesis.	Information on mechanical exposures was collected from questions on hours per week spent:<br>- Sitting, standing, walking, stair climbing, driving, spent in a twisted position.<br><br>Lifting was assessed as how many kilograms were lifted per week.<br>Mechanical exposures were then categorised into:<br>- Static (working in a twisted locked position).<br>- Dynamic (walking with burdens and stair climbing).<br>- Lifted tons (number of lifted kilograms).<br>- Number of lifts (the number of times the person lifted heavy burdens - >40 kg).<br>- Number of jumps (the number of jumps between different levels).	Questionnaire.
**Vingård¸ 1997 (65)**	Case-control.	The study comprised women aged between 50 and 70 years, living in five counties in western Sweden from 1991 to 1994. 230 cases and 273 controls agreed to participate and were enrolled. Cases and controls were matched on age and county or hospital referral area.	In the National registry, information on total hip replacements were obtained based on primary hip osteoarthrosis. Preoperatively, all patients are clinically and radiographically assessed according to defined protocols.	Register.	The following mechanical exposures were assessed:<br>- How many hours spent sitting.<br>- How many hours spent standing or twisted position.<br>- Any lifting, and if so, the weight of the lifted items.<br>- Jumps or movements between different levels.<br>- How many stairs climbed.	Questionnaire.

**Yoshimura, 2000 (66)**	Case-control.	Out of 126 potential cases, 114 cases agreed to participate and were identified from the registration systems of five hospitals in the city of Wakyama and Arita, Japan (103 women and 11 men). The mean age was 63.8 years (SD=10.9). For each case, 1 control was drawn (n=114) from the local population register matched on age, sex, and district of residence.	All cases were listed for total hip arthroplasty due to osteoarthritis. Radiographs of all cases were assessed using Kellgren-Lawrence grading system for osteoarthritis by a single trained observer.	Radiographic.	For each job, the following mechanical exposures were assessed:<br>- Lifting weights of ≥10 kg more than once during an average working week.<br>- Lifting weights of ≥25 kg more than once during an average working week.<br>- Lifting weights of ≥50 kg more than once during an average working week.<br>- Sitting ≥2 hours/day.<br>- Standing ≥2 hours /day.<br>- Kneeling ≥1 hour /day.<br>- Squatting ≥1 hour /day.<br>- Driving ≥4 hours /day.<br>- Walking 3 hours /day.<br>- Climbing ≥30 flights of stairs.	Questionnaire.

ICD: International Classification of Diseases, kg: kilogram, mm: millimeter, n: numbers, OA: osteoarthritis, SD: Standard deviation.

Appendix 6. Data extraction regarding the measure of association

Table 7. Data extraction of the measure of association.

					Men		Women		All	
**Author**	**Exposure**	**Outcome**	**Confounders**	**Categories of exposure**	**Measure of association**	**95% CI**	**Measure of association**	**95% CI**	**Measure of association**	**95% CI**
**Lifting/carrying loads**										
Allen<br>2010	*Lifting*: Lifting/carrying/moving objects weighing >10 lbs. at the longest job participants held measured on a 5-point scale and dichotomised.<br>Radiographic: no OA, exposed n=716 and OA, exposed n=367.<br>Symptomatic: no OA, exposed n=926 and OA, exposed n=146.	Radiographic and symptomatic OA.	Age, sex, race, BMI, smoking (ever and current vs. never), prior knee injury, and household tasks.	Radiographic OA:<br>- Never, seldom, sometimes<br>- Often or always<br><br>Symptomatic OA:<br>- Never, seldom, sometimes<br>- Often or always	 -<br>-<br><br>-<br>-	 -<br>-<br><br>-<br>-	 -<br>-<br><br>-<br>-	 -<br>-<br><br>-<br>-	 1.00 OR<br>1.20 OR<br><br>1.00 OR<br>1.67 OR	 -<br>0.98 – 1.46<br><br>-<br>1.26 – 2.23
	Lifting: If the participants had ever held a job requiring lifting 10 kg ≥10x/week. This was compared to those who did and did not.<br>Radiographic: no OA, exposed n=670 and OA, exposed n=315.<br>Symptomatic: no OA, exposed n=851 and OA, exposed n=132.	Radiographic and symptomatic OA.	Age, sex, race, BMI, smoking (ever and current vs. never), prior knee injury, and household tasks.	Radiographic OA:<br>- No<br>- Yes<br><br>Symptomatic OA:<br>- No<br>- Yes	 -<br>-<br><br>-<br>-	 -<br>-<br><br>-<br>-	 -<br>-<br><br>-<br>-	 -<br>-<br><br>-<br>-	 1.00 OR<br>1.10 OR<br><br>1.00 OR<br>1.71 OR	 -<br>0.90 – 1.36<br><br>-<br>1.28 – 2.29
	Lifting: If the participants had ever held a job requiring lifting 20 kg ≥10x/week. This was compared to those who did and did not.<br>Radiographic: no OA, exposed n=402 and OA, exposed n=177.<br>Symptomatic: no OA, exposed n=501 and OA, exposed n=75.	Radiographic and symptomatic OA.	Age, sex, race, BMI, smoking (ever and current vs. never), prior knee injury, and household tasks.	Radiographic OA:<br>- No<br>- Yes<br><br>Symptomatic OA:<br>- No<br>- Yes	 -<br>-<br><br>-<br>-	 -<br>-<br><br>-<br>-	 -<br>-<br><br>-<br>-	 -<br>-<br><br>-<br>-	 1.00 OR<br>1.03 OR<br><br>1.00 OR<br>1.63 OR	 -<br>0.80 – 1.32<br><br>-<br>1.15 – 2.30
	Lifting: If the participants had ever held a job requiring lifting 50 kg ≥10x/week. This was compared to those who did not.<br>Radiographic: no OA, exposed n=148 and OA, exposed n=75.<br>Symptomatic: no OA, exposed n=183 and OA, exposed n=38.	Radiographic and symptomatic OA.	Age, sex, race, BMI, smoking (ever and current vs. never), prior knee injury, and household tasks.	Radiographic OA:<br>- No<br>- Yes<br><br>Symptomatic OA:<br>- No<br>- Yes	 -<br>-<br><br>-<br>-	 -<br>-<br><br>-<br>-	 -<br>-<br><br>-<br>-	 -<br>-<br><br>-<br>-	 1.00 OR<br>1.02 OR<br><br>1.00 OR<br>1.88 OR	 -<br>0.73 – 1.43<br><br>-<br>1.20 – 2.92

Coggon<br>1998	*Lifting*: heavy occupational lifting was assessed as duration of lifting ≥25 kg >10 times in an average working day up to the age of 30 years (n=611 cases, and n=611 controls).	Radiographic OA.	BMI, presence of Heberden's nodes, and history of hip injury. Matched on age, sex and general practice.	- 0 years<br>- 0.1-4.9 years<br>- 5.0-9.9 years<br>- ≥10.0 years	1.0 OR<br>0.6 OR<br>1.6 OR<br>2.7 OR	-<br>0.2 – 1.3<br>0.7 – 3.7<br>1.4 – 5.1	1.0 OR<br>1.0 OR<br>0.8 OR<br>1.2 OR	-<br>0.5 – 2.0<br>0.4 – 1.7<br>0.5 – 2.6	1.0 OR<br>0.8 OR<br>1.0 OR<br>1.9 OR	-<br>0.5 – 1.3<br>0.6 – 1.7<br>1.2 – 3.0
	Lifting: heavy occupational lifting was assessed as duration of lifting ≥25 kg >10 times in an average working day up to 10 years before entry into the study, defined as the date when the case member of matched pair was interviewed (n=611 cases, and n=611 controls).	Radiographic OA.	BMI, presence of Heberden's nodes, and history of hip injury. Matched on age, sex and general practice.	- 0 years<br>- 0.1-9.9 years<br>- 10.0-19.9 years<br>- ≥20.0 years	1.0 OR<br>0.8 OR<br>1.5 OR<br>2.3 OR	-<br>0.4 – 1.7<br>0.6 – 3.8<br>1.3 – 4.4	1.0 OR<br>1.1 OR<br>1.4 OR<br>0.8 OR	-<br>0.6 – 1.7<br>0.7 – 2.9<br>0.4 – 1.5	1.0 OR<br>0.9 OR<br>1.2 OR<br>1.5 OR	-<br>0.6 – 1.4<br>0.7 – 2.2<br>1.0 – 2.3
	Lifting: maximum level of lifting for at least 10 years up to the age of 30 years >10 times in an average working day (n=611 cases and n=611 controls).	Radiographic OA.	BMI, presence of Heberden's nodes, and history of hip injury. Matched on age, sex and general practice.	- <10 kg<br>- 10-24 kg<br>- 25-49 kg<br>- ≥50 kg	1.0 OR<br>1.7 OR<br>3.0 OR<br>2.9 OR	-<br>0.9 – 3.4<br>1.5 – 6.3<br>1.3 – 6.4	1.0 OR<br>1.0 OR<br>0.8 OR<br>1.7 OR	-<br>0.6 – 1.8<br>0.3 – 2.5<br>0.5 – 6.1	1.0 OR<br>1.2 OR<br>1.9 OR<br>2.1 OR	-<br>0.8 – 1.9<br>1.1 – 3.4<br>1.1 – 3.9
	Lifting: maximum level of lifting for at least 10 years up to 10 years before entry into the study, defined as the date when the case member of matched pair was interviewed, >10 times in an average working day (n=611 cases and n=611 controls).	Radiographic OA.	BMI, presence of Heberden's nodes, and history of hip injury. Matched on age, sex and general practice.	- <10 kg<br>- 10-24 kg<br>- 25-49 kg<br>- ≥50 kg	1.0 OR<br>1.4 OR<br>1.9 OR<br>3.2 OR	-<br>0.7 – 3.0<br>0.9 – 3.9<br>1.6 – 6.5	1.0 OR<br>0.9 OR<br>0.9 OR<br>1.1 OR	-<br>0.6 – 1.3<br>0.5 – 1.7<br>0.5 – 2.5	1.0 OR<br>1.0 OR<br>1.1 OR<br>1.8 OR	-<br>0.7 – 1.4<br>0.7 – 1.8<br>1.1 – 2.9
Croft<br>1992	*Lifting*: years of exposure to lifting or moving weights >56 lbs (>25.4 kg) by hand (n=262 referents, n=224 all cases, and n=49 severe cases).	Radiographic OA.	Age and hospital group.	All cases:<br>- <1 year<br>- 1-19 years<br>- ≥20 years<br>Severe cases:<br>- <1 year<br>- 1-19 years<br>- ≥20 years	 1.0 OR<br>0.9 OR<br>1.2 OR<br><br>1.0 OR<br>1.2 OR<br>2.5 OR	 -<br>0.6 – 1.4<br>0.7 – 1.9<br><br>-<br>0.5 – 2.9<br>1.1 – 5.7	 -<br>-<br>-<br><br>-<br>-<br>-	 -<br>-<br>-<br><br>-<br>-<br>-	 -<br>-<br>-<br><br>-<br>-<br>-	 -<br>-<br>-<br><br>-<br>-<br>-
Jacobsson<br>1987	*Heavy lifting*: was assessed as if the participants had been subject to heavy lifting (n=342).	Radiographic OA.	None.	- No<br>- Yes	1.00 OR<br>2.37 OR	-<br>1.30 – 4.32	-<br>-	-<br>-	-<br>-	-<br>-
Kaila-Kangas<br>2011	*Lifting*: exposure to lifting was assessed using a question on manual handling of heavy objects such as lifting, carrying, or pushing loads over 20 kg on	Clinical examination.	Age, BMI, smoking, and traumatic fractures.	- No<br>- Yes	1.0 OR<br>2.0 OR	-<br>1.0 – 4.0	1.0 OR<br>1.8 OR	-<br>1.1 – 2.8	1.0 OR<br>1.8 OR	-<br>1.2 – 2.7

	average of at least 10 times per working day (n=6556).									
	Lifting: exposure to lifting was assessed using a question on manual handling of heavy objects such as lifting, carrying, or pushing loads over 20 kg on average of at least 10 times per working day depending on years (n=6556).	Clinical examination.	Age, BMI, smoking, and traumatic fractures.	- 0 years<br>- 1-12 years<br>- 13-24 years<br>- >24 years	1.0 OR<br>1.1 OR<br>2.2 OR<br>2.3 OR	-<br>0.4 – 3.2<br>0.8 – 5.9<br>1.2 – 4.3	1.0 OR<br>1.6 OR<br>3.8 OR<br>1.2 OR	-<br>0.7 – 3.5<br>1.7 – 8.1<br>0.7 – 2.1	1.0 OR<br>1.4 OR<br>2.8 OR<br>1.8 OR	-<br>0.7 – 2.6<br>1.5 – 5.0<br>1.1 – 2.4
Lau<br>2000	*Lifting*: was assessed as lifting ≥10 kg during a working week (n=30 cases and n=90 controls for men and n=108 cases and n=324 controls for women).	Radiographic OA.	Men: adjusted for history of joint injury. Matched on age.<br><br>Women: adjusted for height, weight, history of joint injury, regular sports activities. Matched on age.	- No<br>- 1-10 times<br>- >10 times	1.0 OR<br>1.8 OR<br>3.1 OR	-<br>0.4 – 8.1<br>0.7 – 14.3	1.0 OR<br>0.7 OR<br>2.4 OR	-<br>0.3 – 1.7<br>1.1 – 5.3	-<br>-<br>-	-<br>-<br>-
	Lifting: was assessed as lifting ≥50 kg during a working week (n=30 cases and n= 90 controls).	Radiographic OA.	None. Matched on age.	- No<br>- 1-10 times<br>- >10 times	1.0 OR<br>8.5 OR<br>9.6 OR	-<br>1.6 – 45.3<br>2.2 – 42.2	1.0 OR<br>2.0 OR<br>2.9 OR	-<br>0.9 – 4.6<br>1.5 – 5.6	-<br>-<br>-	-<br>-<br>-
Lau<br>2007	*Lifting*: was assessed as lifting 10 kg or more during a working week (n=30 cases and 90 controls for men and n=108 cases and n=324 controls for women).	Radiographic OA.	BMI and injury. Matched on age	- No<br>- 1-10 times<br>- >10 times	1.00 OR<br>2.13 OR<br>4.15 OR	-<br>0.59 – 7.72<br>1.23 – 14.01	1.00 OR<br>0.74 OR<br>3.24 OR	-<br>0.32 – 1.74<br>1.71 – 6.14	1.00 OR<br>1.01 OR<br>3.17 OR	-<br>0.51 – 2.00<br>1.83 – 5.52
	Lifting: was assessed as lifting 50 kg or more during a working week (n=30 cases and 90 controls).	Radiographic OA.	BMI and injury. Matched on age	- No<br>- 1-10 times<br>- >10 times	1.00 OR<br>14.00 OR<br>9.40 OR	-<br>1.96 – 100.01<br>1.26 – 70.42	1.00 OR<br>3.53 OR<br>2.71 OR	-<br>1.33 – 9.40<br>1.21 – 6.10	1.00 OR<br>4.60 OR<br>3.39 OR	-<br>2.00 – 10.57<br>1.63 – 7.04
Olsen<br>1994	*Tons lifted*: was assessed by asking how many kilograms were lifted per week and was collected from the start of the occupational career to the year of diagnosis, aggregated for men's work life up to 49th year of age. It was then defined into three exposure groups (n=239 cases and n=302 controls).	Register information on first-time prosthesis.	Age, BMI, smoking, and sports activities.	- Low<br>- Medium<br>- High	1.00 OR<br>1.58 OR<br>1.84 OR	-<br>NS<br>NS	-<br>-<br>-	-<br>-<br>-	-<br>-<br>-	-<br>-<br>-
	number of lifts >40 kg: was assessed by asking how many kilograms were lifted per week	Register information on first-time prosthesis.	Age, BMI, smoking, and sports activities.	- Low<br>- Medium<br>- High	1.00 OR<br>1.38 OR<br>2.48 OR	-<br>NS<br>NS	-<br>-<br>-	-<br>-<br>-	-<br>-<br>-	-<br>-<br>-

	and was collected from the start of the occupational career to the year of diagnosis, aggregated for men's work life up to 49th year of age. It was then defined into three exposure groups (n=239 cases and n=302 controls).									
Rubak<br>2014	*Lifting*: was assessed as ton-years calculating lifting 1 ton per day for 1 year (n=1776 case-control sets including at least 1 case and 1 control, divided in 861 sets for women and 915 sets for men).	Register information on total hip replacement due to OA.	One occupational exposure at a time adjusted for body mass index at age 25, change in body mass index, pack-years of smoking, previous fracture of a lower extremity, familial predisposition, endurance and contact sport at age 25, and region of residence. Matched on age.	Men:<br>- 0 years<br>- >0 to <10 years<br>- 10 to <20 years<br>- 20 to 115<br>Women:<br>- 0 years<br>- >0 to >10 years<br>- 10 to <20 years<br>- 20 to 86	 1.00 OR<br>0.99 OR<br>0.89 OR<br>1.35 OR<br><br>-<br>-<br>-<br>-	 -<br>0.75 – 1.30<br>0.67 – 1.17<br>1.05 – 1.74<br><br>-<br>-<br>-<br>-	 -<br>-<br>-<br>-<br><br>1.00 OR<br>1.15 OR<br>0.81 OR<br>1.00 OR	 -<br>-<br>-<br>-<br><br>-<br>0.87 – 1.53<br>0.61 – 1.09<br>0.72 – 1.35	 -<br>-<br>-<br>-<br><br>-<br>-<br>-<br>-	 -<br>-<br>-<br>-<br><br>-<br>-<br>-<br>-
Solovieva<br>2018	*Heavy lifting*: was assessed from a job-exposure matrix dichotomising heavy lifting (n=574,617 men and n=561,037 women).	Register information on disability retirement due to hip OA.	Age, heavy physical work, kneeling or squatting, sitting, and standing or moving.	- No<br>- Yes	1.00 HR<br>1.23 HR	-<br>1.02 – 1.48	1.00 HR<br>1.08 HR	-<br>0.87 – 1.34	-<br>-	-<br>-
Vingård<br>1997	*Lifting*: was assessed based on how many kilos the participant lifted each day and divided into three subclasses on the basis of the exposure distribution (n=230 cases and n= 273 controls).	Total hip replacement due to OA.	Age, BMI, smoking, sports activities, number of children, and hormone therapy.	- Low<br>- Medium<br>- High exposure	-<br>-<br>-	-<br>-<br>-	1.0 RR<br>1.1 RR<br>1.5 RR	-<br>0.7 – 1.7<br>0.9 – 2.5	-<br>-<br>-	-<br>-<br>-
Vingård<br>1991	*Lifted tons*: was assessed as the number of lifted kilograms and divided into three subclasses on the basis of the exposure distribution (n=233 cases and n=302 controls).	Total hip replacement due to OA.	Age, BMI, smoking, and sport activities up to the age of 29 years.	- Low<br>- Medium<br>- High	1.00 RR<br>1.58 RR<br>1.84 RR	-<br>0.93 – 2.66<br>1.12 – 3.03	-<br>-<br>-	-<br>-<br>-	-<br>-<br>-	-<br>-<br>-
	Number of lifts: was assessed as the number of times a person lifted heavy burdens (>40 kg) and divided into three subclasses on the basis of the exposure distribution (n=233 cases and n=302 controls).	Total hip replacement due to OA.	Age, BMI, smoking, and sport activities up to the age of 29 years.	- Low<br>- Medium<br>- High	1.00 RR<br>1.38 RR<br>2.40 RR	-<br>0.81 – 2.36<br>1.50 – 2.83	-<br>-<br>-	-<br>-<br>-	-<br>-<br>-	-<br>-<br>-

Yoshimura<br>2000	*Lifting*: assessed as weights lifted more than once (kg) during an average working week at the first job (n= 103 cases and n=103 controls).	Radiographic OA.	History of knee pain and age left school. Matched on age, sex, and district of residence.	First job:<br>- Lifting 0 kg<br>- Lifting ≥10 kg<br>- Lifting ≥25 kg<br>- Lifting ≥50 kg	 -<br>-<br>-<br>-	 -<br>-<br>-<br>-	 -<br>-<br>-<br>-	 -<br>-<br>-<br>-	 1.0 OR<br>1.2 OR<br>3.5 OR<br>-	 -<br>0.6 – 2.4<br>1.3 – 9.7<br>-
	Lifting: assessed as weights lifted more than once (kg) during an average working week at the main job (n= 103 cases and n=103 controls).	Radiographic OA.	History of knee pain and age left school. Matched on age, sex, and district of residence.	Main job:<br>- Lifting 0 kg<br>- Lifting ≥10 kg<br>- Lifting ≥25 kg<br>- Lifting ≥50 kg	 -<br>-<br>-<br>-	 -<br>-<br>-<br>-	 -<br>-<br>-<br>-	 -<br>-<br>-<br>-	 1.0 OR<br>1.2 OR<br>1.5 OR<br>4.1 OR	 -<br>0.6 – 2.1<br>0.7 – 3.0<br>1.1 – 15.2
**Awkward posture**										
Allen<br>2010	*Posture*: bending/twisting/reaching at the longest job participants held, measured on a 5-point scale and dichotomised.<br>Radiographic: no OA, exposed n=980 and OA, exposed n=480.<br>Symptomatic: no OA, exposed n=1264 and OA, exposed n=271.	Radiographic and symptomatic OA.	Age, sex, race, BMI, smoking (ever and current vs. never), prior knee injury, and household tasks.	Radiographic OA:<br>- Never, seldom, sometimes<br>- Often or always<br><br>Symptomatic OA:<br>- Never, seldom, sometimes<br>- Often or always	 -<br><br>-<br><br><br>-<br><br>-	 -<br><br>-<br><br><br>-<br><br>-	 -<br><br>-<br><br><br>-<br><br>-	 -<br><br>-<br><br><br>-<br><br>-	 1.00 OR<br><br>1.21 OR<br><br><br>1.00 OR<br><br>1.60 OR	 -<br><br>0.98 – 1.48<br><br><br>-<br><br>1.18 – 2.17
Croft<br>1992	*Bending*: years of exposure to bending for >2 hours a day (n=259 referents, n=212 all cases, and n=46 severe cases).	Radiographic OA.	Age and hospital group.	All cases:<br>- <1 year<br>- 1–19 years<br>- ≥20 years<br>Severe cases:<br>- <1 year<br>- 1–19 years<br>- ≥20 years	 1.0 OR<br>0.7 OR<br>1.2 OR<br><br>1.0 OR<br>0.8 OR<br>1.9 OR	 -<br>0.4 – 1.1<br>0.7 – 1.9<br><br>-<br>0.3 – 2.0<br>0.8 – 4.5	 -<br>-<br>-<br><br>-<br>-<br>-	 -<br>-<br>-<br><br>-<br>-<br>-	 -<br>-<br>-<br><br>-<br>-<br>-	 -<br>-<br>-<br><br>-<br>-<br>-
Rijs<br>2014	*Positions*: was assessed with a job-exposure matrix measuring work performed in uncomfortable positions (n=271 for current job and n=971 for longest job held).	Self-report and general practitioner data on hip OA.	Age and sex.	Current job:<br>- Low<br>- Moderate<br>Longest job:<br>- Low<br>- Moderate	 -<br>-<br><br>-<br>-	 -<br>-<br><br>-<br>-	 -<br>-<br><br>-<br>-	 -<br>-<br><br>-<br>-	 1.0 OR<br>2.5 OR<br><br>1.0 OR<br>1.5 OR	 -<br>0.9 – 7.5<br><br>-<br>0.99 – 2.4
Vingård<br>1997	*Twisted position*: was assessed based on hours working in a twisted position and divided into three subclasses on the basis of the exposure distribution (n=230 cases and n= 273 controls).	Total hip replacement due to OA.	Age, BMI, smoking, sports activities, number of children, and hormone therapy.	- Low<br>- Medium<br>- High	-<br>-<br>-	-<br>-<br>-	1.0 RR<br>1.1 RR<br>1.6 RR	-<br>0.7 – 1.8<br>0.9 – 2.6	-<br>-<br>-	-<br>-<br>-
Vingård<br>1991	*Static*: was assessed as working in a twisted locked position and divided into three subclasses based on the exposure	Total hip replacement due to OA.	Age, BMI, smoking, and sport activities up to the age of 29 years.	- Low<br>- Medium<br>- High	1.00 RR<br>1.21 RR<br>2.92 RR	-<br>0.64 – 2.31<br>1.69 – 5.05	-<br>-<br>-	-<br>-<br>-	-<br>-<br>-	-<br>-<br>-

	distribution (n=233 cases and n=302 controls).									
**Standing**										
Allen<br>2010	*Stand*: standing at the longest job participants held, measured on a 5-point scale.<br>Radiographic: no OA, exposed n=1051 and OA, exposed n=524.<br>Symptomatic: no OA, exposed n=1379 and OA, exposed n=187.	Radiographic and symptomatic OA.	Age, sex, race, BMI, smoking (ever and current vs. never), prior knee injury, and household tasks.	Radiographic OA:<br>- Never, seldom, sometimes<br>- Often or always<br><br>Symptomatic OA:<br>- Never, seldom, sometimes<br>- Often or always	-<br>-<br>-<br>-	-<br>-<br>-<br>-	-<br>-<br>-<br>-	-<br>-<br>-<br>-	1.00 OR<br>1.15 OR<br>1.00 OR<br>1.30 OR	-<br>0.94 – 1.42<br>-<br>0.97 – 1.75
Coggon<br>1998	*Standing*: standing for >2 hours in an average working day for up to 10 years before entry into the study (n=611 cases and n=611 controls).	Radiographic OA.	BMI, Heberden's nodes, and history of hip injury. Matched on age, sex and general practice.	- 0 years<br>- 0.1-9.9 years<br>- 10.0-19.9 years<br>- ≥20.0 years	1.0 OR<br>0.2 OR<br>0.4 OR<br>0.5 OR	-<br>0.0 – 1.4<br>0.1 – 2.4<br>0.1 – 2.3	1.0 OR<br>1.1 OR<br>1.1 OR<br>1.3 OR	-<br>0.6 – 2.0<br>0.6 – 1.9<br>0.7 – 2.1	1.0 OR<br>1.0 OR<br>1.0 OR<br>1.2 OR	-<br>0.6 – 1.7<br>0.6 – 1.7<br>0.7 – 1.9
Croft<br>1992	*Standing*: years of exposure to standing for >2 hours a day (n=284, n= 232 all cases, and n=51 severe cases).	Radiographic OA.	Age and hospital group.	All cases:<br>- <20 year<br>- 20–39 years<br>- ≥40 years<br>Severe cases:<br>- <20 year<br>- 20–39 years<br>- ≥40 years	1.0 OR<br>1.8 OR<br>1.7 OR<br>1.0 OR<br>1.5 OR<br>2.7 OR	-<br>1.0 – 3.1<br>1.0 – 2.8<br>-<br>0.5 – 4.8<br>1.0 – 7.3	-<br>-<br>-<br>-<br>-<br>-	-<br>-<br>-<br>-<br>-<br>-	-<br>-<br>-<br>-<br>-<br>-	-<br>-<br>-<br>-<br>-<br>-
Cvijetic<br>1999	*Standing:* Years of work in a standing position (>80 % of time in a standing position) - (34 men and 38 women).	Radiographic OA.	Age and BMI.	<20 years<br>20-29.9 years<br>>30 years	1.0 OR<br>2.09 OR<br>1.83 OR	-<br>0.98 – 3.17<br>0.68 – 3.56	1.0 OR<br>1.94 OR<br>3.24 OR	-<br>0.64 – 2.14<br>0.51 – 4.02	-<br>-<br>-	-<br>-<br>-
Jacobsson<br>1987	*Standing*: was assessed as if the participants had been subject to too much standing (n=342).	Radiographic OA.	None.	- No<br>- Yes	1.00 OR<br>0.78 OR	-<br>0.49 – 1.24	-<br>-	-<br>-	-<br>-	-<br>-
Vingård<br>1997	*Standing*: was assessed based on hours working in standing position and divided into three subclasses based on the exposure distribution (n=230 cases and n= 273 controls).	Total hip replacement due to OA.	Age, BMI, smoking, sports activities, number of children, and hormone therapy.	- Low<br>- Medium<br>- High	-<br>-<br>-	-<br>-<br>-	1.0 RR<br>1.4 RR<br>1.6 RR	-<br>0.8 – 2.2<br>0.9 – 2.8	-<br>-<br>-	-<br>-<br>-
Yoshimura<br>2000	*Standing*: was assessed as standing ≥2 hours (n=103 cases and n=103 controls).	Radiographic OA.	History of knee pain and age left school. Matched on age, sex, and district of residence.	First job:<br>- No<br>- Yes<br>Main job:<br>- No	-<br>-<br>-	-<br>-<br>-	-<br>-<br>-	-<br>-<br>-	1.0 OR<br>1.4 OR<br>1.0 OR	-<br>0.7 – 2.8<br>-

				- Yes	-	-	-	-	1.1 OR	0.6 – 2.3
**Walking**										
Allen<br>2010	*Walking*: ever held a job requiring walking >50 % of their time.<br>Radiographic: no OA, exposed n=703 and OA, exposed n=344.<br>Symptomatic: no OA, exposed n=914 and OA, exposed n=131.	Radiographic and symptomatic OA.	Age, sex, race, BMI, smoking (ever and current vs. never), prior knee injury, and household tasks.	Radiographic OA:<br>- <50 %<br>- >50 %<br><br>Symptomatic OA:<br>- <50 %<br>- >50 %	 -<br>-<br><br><br>-<br>-	 -<br>-<br><br><br>-<br>-	 -<br>-<br><br><br>-<br>-	 -<br>-<br><br><br>-<br>-	 1.00 OR<br>1.15 OR<br><br><br>1.00 OR<br>1.19 OR	 -<br>0.95 – 1.40<br><br><br>-<br>0.90 – 1.56
	Walk: walking at the longest job the participants held measured on a 5-point scale and dichotomised.<br>Radiographic: no OA, exposed n=1174 and OA, exposed n=568.<br>Symptomatic: no OA, exposed n=1525 and OA, exposed n=203.	Radiographic and symptomatic OA.	Age, sex, race, BMI, smoking (ever and current vs. never), prior knee injury, and household tasks.	Radiographic OA:<br>- Never, seldom, sometimes<br>- Often or always<br><br>Symptomatic OA:<br>- Never, seldom, sometimes<br>- Often or always	 -<br><br>-<br><br><br>-<br><br>-	 -<br><br>-<br><br><br>-<br><br>-	 -<br><br>-<br><br><br>-<br><br>-	 -<br><br>-<br><br><br>-<br><br>-	 1.00 OR<br><br>1.15 OR<br><br><br>1.00 OR<br><br>1.38 OR	 -<br><br>0.92 – 1.43<br><br><br>-<br><br>1.00 – 1.91
Coggon<br>1998	*Walking*: walking for >2 miles (3.2 km) in an average working day for up to 10 years before entry into the study (n=611 cases and n=611 controls).	Radiographic OA.	BMI, Heberden's nodes, and history of hip injury. Matched on age, sex and general practice.	- 0 years<br>- 0.1-9.9 years<br>- 10.0-19.9 years<br>- ≥20.0 years	1.0 OR<br>0.8 OR<br>1.1 OR<br>1.2 OR	-<br>0.4 – 1.9<br>0.4 – 2.5<br>0.6 – 2.5	1.0 OR<br>1.5 OR<br>1.5 OR<br>1.3 OR	-<br>1.0 – 2.3<br>1.0 – 2.3<br>0.8 – 2.0	1.0 OR<br>1.3 OR<br>1.4 OR<br>1.3 OR	-<br>0.9 – 1.9<br>0.9 – 2.0<br>0.9 – 1.8
Croft<br>1992	*Walking*: years of exposure to walking >2 miles (3.2 km) a day (n=275 referents, n=229 all cases, and n=51 severe cases).	Radiographic OA.	Age and hospital group.	All cases:<br>- <1 year<br>- 1–19 years<br>- ≥20 years<br>Severe cases:<br>- <1 year<br>- 1–19 years<br>- ≥20 years	 1.0 OR<br>0.8 OR<br>0.8 OR<br><br>1.0 OR<br>1.4 OR<br>1.6 OR	 -<br>0.5 – 1.5<br>0.5 – 1.5<br><br>-<br>0.4 – 4.6<br>0.5 – 5.1	 -<br>-<br>-<br><br>-<br>-<br>-	 -<br>-<br>-<br><br>-<br>-<br>-	 -<br>-<br>-<br><br>-<br>-<br>-	 -<br>-<br>-<br><br>-<br>-<br>-
	Walking on rough ground: years of exposure to walking >2 miles (3.2 km) over rough ground (n=264 referents, n=218 all cases, and n=49 severe cases).	Radiographic OA.	Age and hospital group.	All cases:<br>- <1 year<br>- 1–19 years<br>- ≥20 years<br>Severe cases:<br>- <1 year<br>- 1–19 years<br>- ≥20 years	 1.0 OR<br>1.2 OR<br>1.0 OR<br><br>1.0 OR<br>2.0 OR<br>1.9 OR	 -<br>0.7 – 1.8<br>0.6 – 1.6<br><br>-<br>0.9 – 4.3<br>0.9 – 4.1	 -<br>-<br>-<br><br>-<br>-<br>-	 -<br>-<br>-<br><br>-<br>-<br>-	 -<br>-<br>-<br><br>-<br>-<br>-	 -<br>-<br>-<br><br>-<br>-<br>-
Jacobsson<br>1987	*Walking*: was assessed as if the participants had been subject to too much walking (n=342).	Radiographic OA.	None.	- No<br>- Yes	1.00 OR<br>1.56 OR	-<br>0.86 – 2.80	-<br>-	-<br>-	-<br>-	-<br>-

Lau<br>2000	*Walking*: was assessed as walking for ≥2 hours on an average working day (n=30 cases and n=90 controls).	Radiographic OA.	History of joint injury. Matched on age.	- No<br>- Yes	1.0 OR<br>1.3 OR	-<br>0.3 – 6.7	-<br>-	-<br>-	-<br>-	-<br>-
	Walking: was assesses as walking for ≥2 hours on an average working day (n=108 cases and n=324 controls).	Radiographic OA.	None. Matched on age.	- No<br>-Yes	-<br>-	-<br>-	1.0 OR<br>1.4 OR	-<br>0.9 – 2.3	-<br>-	-<br>-
Lau<br>2007	*Walking*: was assesses as walking for ≥2 hours on an average working day (n=30 cases and n=90 controls for men and n=108 cases and n=324 controls for women).	Radiographic OA.	BMI and injury. Matched on age.	- No<br>- Yes	1.00 OR<br>4.02 OR	-<br>1.04 – 15.56	1.00 OR<br>1.16 OR	-<br>0.69 – 1.96	1.00 OR<br>1.41 OR	-<br>0.88 – 2.25
Yoshimura<br>2000	*Walking*: was assessed as walking ≥3 km (n=103 cases and n=103 controls).	Radiographic OA.	History of knee pain and age left school. Matched on age, sex, and district of residence.	First job:<br>- No<br>- Yes<br>Main job:<br>- No<br>- Yes	 -<br>-<br><br>-<br>-	 -<br>-<br><br>-<br>-	 -<br>-<br><br>-<br>-	 -<br>-<br><br>-<br>-	 1.0 OR<br>1.0 OR<br><br>1.0 OR<br>1.2 OR	 -<br>0.4 – 2.2<br><br>-<br>0.6 – 2.4
**Standing or walking**										
Kontio<br>2020	*Standing or walking*: was assessed as cumulative exposure to standing or walking for ≥5 hours per day (n=4405 with no OA and n=87 for hip OA).	Hospitalisation due to hip OA.	Age and sex.	- <1 year<br>- 1-10 years<br>- 11-20 years<br>- >20 years	-<br>-<br>-<br>-	-<br>-<br>-<br>-	-<br>-<br>-<br>-	-<br>-<br>-<br>-	1.00 HR<br>0.90 HR<br>1.39 HR<br>1.06 HR	-<br>0.48 – 1.72<br>0.75 – 2.58<br>0.61 – 1.84
Solovieva<br>2018	*Standing or moving*: was assessed from a job-exposure matrix dichotomising standing or moving (n=574,617 men and n=561,037 women).	Disability retirement due to OA.	Age, heavy physical work, kneeling or squatting, heavy lifting, standing or moving.	- No<br>- Yes	1.00 HR<br>1.24 HR	-<br>1.04 – 1.48	1.00 HR<br>1.13 HR	-<br>0.98 – 1.29	-<br>-	-<br>-
Rubak<br>2014	*Standing*: was assessed as exposure to standing/walking 6 hours per working day for 1 year (n=1776 case-control sets including at least 1 case and 1 control, divided in 861 sets for women and 915 sets for men).	Total hip replacement due to OA.	One occupational exposure at a time adjusted for body mass index at age 25, change in body mass index, pack-years of smoking, previous fracture of a lower extremity, familial predisposition, endurance and contact sport at age 25, and region of residence. Matched on age.	- 0 years<br>- >0 to <10 years<br>- 10 to <20 years<br>- 20 to 29 years	1.00 OR<br>1.13 OR<br>1.14 OR<br>0.99 OR	-<br>0.85 – 1.50<br>0.87 – 1.48<br>0.77 – 1.28	1.00 OR<br>0.91 OR<br>0.99 OR<br>1.03 OR	-<br>0.56 – 1.48<br>0.74 – 1.25<br>0.78 – 1.35	-<br>-<br>-<br>-	-<br>-<br>-<br>-

**Kneeling**										
Allen<br>2010	*Kneeling*: ever held a job requiring kneeling >50 % of their time.<br>Radiographic: no OA, exposed n=209 and OA, exposed n=85.<br>Symptomatic: no OA, exposed n=256 and OA, exposed n=36.	Radiographic and symptomatic OA.	Age, sex, race, BMI, smoking (ever and current vs. never), prior knee injury, and household tasks.	Radiographic OA:<br>- <50 %<br>- >50 %<br><br>Symptomatic OA:<br>- <50 %<br>- >50 %	 -<br>-<br><br><br>-<br>-	 -<br>-<br><br><br>-<br>-	 -<br>-<br><br><br>-<br>-	 -<br>-<br><br><br>-<br>-	 1.00 OR<br>0.84 OR<br><br><br>1.00 OR<br>1.15 OR	 -<br>0.62 – 1.14<br><br><br>-<br>0.76 – 1.73
	Crouch or kneel: crouching or kneeling at the longest job participants held, measured on a 5-point scale and dichotomised.<br>Radiographic: no OA, exposed n=193 and OA, exposed n=88.<br>Symptomatic: no OA, exposed n=243 and OA, exposed n=37.	Radiographic and symptomatic OA.	Age, sex, race, BMI, smoking (ever and current vs. never), prior knee injury, and household tasks.	Radiographic OA:<br>- Never, seldom, sometimes<br>- Often or always<br><br>Symptomatic OA:<br>- Never, seldom, sometimes<br>- Often or always	 -<br><br>-<br><br><br>-<br><br>-	 -<br><br>-<br><br><br>-<br><br>-	 -<br><br>-<br><br><br>-<br><br>-	 -<br><br>-<br><br><br>-<br><br>-	 1.00 OR<br><br>1.15 OR<br><br><br>1.00 OR<br><br>1.45 OR	 -<br><br>0.84 – 1.56<br><br><br>-<br><br>0.95 – 2.21
Coggon<br>1998	*Kneeling*: kneeling for >1 hour in an average working day for up to 10 years before entry into the study (n=611 cases and n=611 controls).	Radiographic OA.	BMI, Heberden's nodes, and history of hip injury. Matched on age, sex and general practice.	- 0 years<br>- 0.1-9.9 years<br>- 10.0-19.9 years<br>- ≥20.0 years	1.0 OR<br>0.8 OR<br>2.0 OR<br>1.0 OR	-<br>0.4 – 1.4<br>0.6 – 4.7<br>0.6 – 1.7	1.0 OR<br>0.9 OR<br>0.7 OR<br>1.2 OR	-<br>0.6 – 1.4<br>0.4 – 1.3<br>0.5 – 3.0	1.0 OR<br>0.9 OR<br>1.0 OR<br>1.1 OR	-<br>0.6 – 1.2<br>0.6 – 1.7<br>0.7 – 1.7
Croft<br>1992	*Kneeling*: years of exposure to kneeling for >30 min a day (n=244 referents, n=197 all cases, and n=41).	Radiographic OA.	Age and hospital group.	All cases:<br>- <1 year<br>- 1–19 years<br>- ≥20 years<br>Severe cases:<br>- <1 year<br>- 1–19 years<br>- ≥20 years	 1.0 OR<br>0.6 OR<br>0.7 OR<br><br>1.0 OR<br>0.5 OR<br>1.0 OR	 -<br>0.4 – 1.0<br>0.4 – 1.3<br><br>-<br>0.2 – 1.4<br>0.3 – 3.2	 -<br>-<br>-<br><br>-<br>-<br>-	 -<br>-<br>-<br><br>-<br>-<br>-	 -<br>-<br>-<br><br>-<br>-<br>-	 -<br>-<br>-<br><br>-<br>-<br>-
Lau<br>2000	*Kneeling*: was assessed as kneeling for ≥ 1 hour on an average working day (n=30 cases and n=90 controls).	Radiographic OA.	History of joint injury. Matched on age.	- No<br>- Yes	1.0 OR<br>7.4 OR	-<br>0.7 – 76.9	-<br>-	-<br>-	-<br>-	-<br>-
	Kneeling: was assessed as kneeling for ≥ 1 hour on an average working day (n=108 cases and n=324 controls).	Radiographic OA.	None. Matched on age.	- No<br>- Yes	-<br>-	-<br>-	1.0 OR<br>1.3 OR	-<br>0.7 – 2.5	-<br>-	-<br>-
Lau<br>2007	*Kneeling*: was assessed as kneeling for ≥ 1 hour on an average working day (n=30 cases and n=90 controls for men and	Radiographic OA.	BMI and injury. Matched on age.	- No<br>- Yes	1.00 OR<br>5.22 OR	-<br>0.82 – 33.30	1.00 OR<br>1.52 OR	-<br>0.76 – 3.05	1.00 OR<br>1.70 OR	-<br>0.90 – 3.21

	n=108 cases and n=324 controls for women).									
Yoshimura<br>2000	*Kneeling*: was assessed as kneeling ≥1 hour (n=103 cases and n=103 controls).	Radiographic OA.	History of knee pain and age left school. Matched on age, sex, and district of residence.	First job:<br>- No<br>- Yes<br>Main job:<br>- No<br>- Yes	 -<br>-<br><br>-<br>-	 -<br>-<br><br>-<br>-	 -<br>-<br><br>-<br>-	 -<br>-<br><br>-<br>-	 1.0 OR<br>0.7 OR<br><br>1.0 OR<br>1.0 OR	 -<br>0.4 – 1.4<br><br>-<br>0.5 – 2.1
**Squatting**										
Allen<br>2010	*Squat*: squatting at the longest job participants held, measured on a 5-point scale and dichotomised.<br>Radiographic: no OA, exposed n=350 and OA, exposed n=164.<br>Symptomatic: no OA, exposed n=452 and OA, exposed n=48.	Radiographic and symptomatic OA.	Age, sex, race, BMI, smoking (ever and current vs. never), prior knee injury, and household tasks.	Radiographic OA:<br>- Never, seldom, sometimes<br>- Often or always<br><br>Symptomatic OA:<br>- Never, seldom, sometimes<br>- Often or always	 -<br>-<br><br>-<br>-	 -<br>-<br><br>-<br>-	 -<br>-<br><br>-<br>-	 -<br>-<br><br>-<br>-	 1.00 OR<br>1.03 OR<br><br>1.00 OR<br>1.11 OR	 -<br>0.81 – 1.30<br><br>-<br>0.79 – 1.57
Coggon<br>1998	*Squatting*: squatting for >1 hour in an average working day for up to 10 years before entry into the study (n=611 cases and n=611 controls).	Radiographic OA.	BMI, Heberden's nodes, and history of hip injury. Matched on age, sex and general practice.	- 0 years<br>- 0.1-9.9 years<br>- 10.0-19.9 years<br>- ≥20.0 years	1.0 OR<br>0.9 OR<br>1.4 OR<br>0.9 OR	-<br>0.5 – 1.6<br>0.5 – 3.6<br>0.5 – 1.6	1.0 OR<br>1.1 OR<br>1.5 OR<br>0.7 OR	-<br>0.6 – 1.9<br>0.6 – 3.4<br>0.3 – 1.8	1.0 OR<br>1.0 OR<br>1.5 OR<br>0.9 OR	-<br>0.7 – 1.5<br>0.8 – 2.7<br>0.6 – 1.4
Croft<br>1992	*Squatting*: years of exposure to squatting for >30 min a day (n=238 referents, n=190 all cases, and n=40 severe cases).	Radiographic OA.	Age and hospital group.	All cases:<br>- <1 year<br>- ≥1 year<br>Severe cases:<br>- <1 year<br>- ≥1 year	 1.0 OR<br>0.7 OR<br><br>1.0 OR<br>1.3 OR	 -<br>0.4 – 1.4<br><br>-<br>0.4 – 3.6	 -<br>-<br><br><br>-<br>-	 -<br>-<br><br><br>-<br>-	 -<br>-<br><br><br>-<br>-	 -<br>-<br><br><br>-<br>-
Lau<br>2000	*Squatting*: was assessed as squatting for ≥1 hour on an average working day (n=30 cases and 90 controls).	Radiographic OA.	None. Matched on age.	- No<br>- Yes	1.0 OR<br>1.3 OR	-<br>0.5 – 3.2	-<br>-	-<br>-	-<br>-	-<br>-
	Squatting: was assessed as squatting for ≥1 hour on an average working day (n=108 cases and 324 controls).	Radiographic OA.	Height, weight, history of joint injury, and regular sports activity. Matched on age.	- No<br>- Yes	-<br>-	-<br>-	1.0 OR<br>1.2 OR	-<br>0.5 – 3.0	-<br>-	-<br>-
Lau<br>2007	*Squatting*: was assessed as squatting for ≥1 hour on an average working day (n=30 cases and 90 controls for men and n=108 cases and 324 controls for women).	Radiographic OA.	BMI and injury. Matched on age.	- No<br>- Yes	1.00 OR<br>0.87 OR	-<br>0.29 – 2.65	1.00 OR<br>1.99 OR	-<br>1.07 – 3.71	1.00 OR<br>1.62 OR	-<br>0.95 – 2.78

Yoshimura<br>2000	*Squatting*: was assessed as squatting ≥1 hour (n=103 cases and n=103 controls).	Radiographic OA.	History of knee pain and age left school. Matched on age, sex, and district of residence.	First job:<br>- No<br>- Yes<br>Main job:<br>- No<br>- Yes	 -<br>-<br><br>-<br>-	 -<br>-<br><br>-<br>-	 -<br>-<br><br>-<br>-	 -<br>-<br><br>-<br>-	 1.0 OR<br>1.0 OR<br><br>1.0 OR<br>1.3 OR	 -<br>0.5 – 2.2<br><br>-<br>0.6 – 2.8
**Kneeling or squatting**										
Kontio<br>2020	*Kneeling or squatting*: was assessed as cumulative exposure to kneeling or squatting for ≥1 hour per day (n=4405 with no OA and n=87 for hip OA).	Hospitalisation due to hip OA.	Age and sex.	- <1 year<br>- 1-10 years<br>- 11-20 years<br>- >20 years	-<br>-<br>-<br>-	-<br>-<br>-<br>-	-<br>-<br>-<br>-	-<br>-<br>-<br>-	1.00 HR<br>1.68 HR<br>1.50 HR<br>1.41 HR	-<br>0.96 – 2.95<br>0.75 – 2.98<br>0.79 – 2.50
Solovieva<br>2018	*Kneeling or squatting*: was assessed from a job-exposure matrix dichotomising kneeling or squatting (n=574,617 men and n=561,037 women).	Disability retirement due to OA.	Age, heavy physical work, heavy lifting, sitting, standing or moving.	- No<br>- Yes	1.00 HR<br>1.17 HR	-<br>0.99 – 1.39	1.00 HR<br>1.53 HR	-<br>1.27 – 1.84	-<br>-	-<br>-
**Climbing stairs**										
Allen<br>2010	*Climb stairs*: climbing stairs at the longest job participants held, measured on a 5-point scale and dichotomised.<br>Radiographic: no OA, exposed n=351 and OA, exposed n=167.<br>Symptomatic: no OA, exposed n=456 and OA, exposed n=59.	Radiographic and symptomatic OA.	Age, sex, race, BMI, smoking (ever and current vs. never), prior knee injury, and household tasks.	Radiographic OA:<br>- Never, seldom, sometimes<br>- Often or always<br><br>Symptomatic OA:<br>- Never, seldom, sometimes<br>- Often or always	 -<br>-<br><br>-<br>-	 -<br>-<br><br>-<br>-	 -<br>-<br><br>-<br>-	 -<br>-<br><br>-<br>-	 1.00 OR<br>1.01 OR<br><br>1.00 OR<br>1.17 OR	 -<br>0.80 – 1.28<br><br>-<br>0.84 – 1.62
Coggon<br>1998	*Climbing*: climbing >30 flights of stairs in an average working day for up to 10 years before entry into the study (n=611 cases and n=611 controls).	Radiographic OA.	BMI, Heberden's nodes, and history of hip injury. Matched on age, sex and general practice.	- 0 years<br>- 0.1-9.9 years<br>- 10.0-19.9 years<br>- ≥20.0 years	1.0 OR<br>1.3 OR<br>2.3 OR<br>1.8 OR	-<br>0.7 – 2.5<br>1.1 – 4.9<br>0.9 – 3.4	1.0 OR<br>1.4 OR<br>1.3 OR<br>2.3 OR	-<br>0.8 – 2.2<br>0.4 – 4.0<br>0.8 – 6.3	1.0 OR<br>1.3 OR<br>1.7 OR<br>1.7 OR	-<br>0.9 – 1.9<br>1.0 – 3.1<br>1.0 – 2.8
Croft<br>1992	*Climbing ladders*: years of exposure to climbing ladders (n=264 referents, n=226 all cases, and n=47 severe cases).	Radiographic OA.	Age and hospital group.	All cases:<br>- <1 year<br>- 1–19 years<br>- ≥20 years<br>Severe cases:<br>- <1 year<br>- 1–19 years<br>- ≥20 years	 1.0 OR<br>0.9 OR<br>0.8 OR<br><br>1.0 OR<br>0.8 OR<br>1.6 OR	 -<br>0.6 – 1.4<br>0.5 – 1.5<br><br>-<br>0.3 – 1.8<br>0.7 – 3.8	 -<br>-<br>-<br><br>-<br>-<br>-	 -<br>-<br>-<br><br>-<br>-<br>-	 -<br>-<br>-<br><br>-<br>-<br>-	 -<br>-<br>-<br><br>-<br>-<br>-
	Climbing: years of exposure to climbing >30 flights of stairs a day (n=260 referents, n=225 all cases, and n=50 severe cases).	Radiographic OA.	Age and hospital group.	All cases:<br>- <1 year<br>- ≥1 year<br>Severe cases:	 1.0 OR<br>1.0 OR	 -<br>0.6 – 1.5	 -<br>-	 -<br>-	 -<br>-	 -<br>-

				- <1 year<br>- ≥1 year	1.0 OR<br>1.2 OR	-<br>0.6 – 2.5	-<br>-	-<br>-	-<br>-	-<br>-
Lau<br>2000	*Climbing stairs*: was assessed as climbing ≥15 flights of stairs on an average working day (n=30 cases and n=90 controls for men and n=108 cases and n=324 controls for women).	Radiographic OA.	Men: adjusted for history of joint injury. Matched on age.<br><br>Women: adjusted for height, weight, history of joint injury, and regular sports activities. Matched on age.	- No<br>- Yes	1.0 OR<br>12.5 OR	-<br>1.5 – 104.3	1.0 OR<br>2.3 OR	-<br>0.6 – 8.1	-<br>-	-<br>-
Lau<br>2007	*Climbing*: was assessed as climbing ≥15 flights of stairs on an average working day (n=30 cases and n=90 controls for men and n=108 cases and n=324 controls for women).	Radiographic OA.	BMI and injury. Matched on age.	- Yes	15.90 OR	2.30 – 109.9	2.60 OR	0.97 – 7.08	4.13 OR	1.78 – 9.60
Vingård<br>1997	*Climbing*: was assessed based on how many stairs climbed every day on work and divided into three subclasses on the basis of the exposure distribution (n=230 cases and n= 273 controls).	Total hip replacement due to OA.	Age, BMI, smoking, sports activities, number of children, and hormone therapy.	- Low<br>- Medium<br>- High	-<br>-<br>-	-<br>-<br>-	1.0 RR<br>1.3 RR<br>2.1 RR	-<br>0.8 – 2.2<br>1.2 – 3.6	-<br>-<br>-	-<br>-<br>-
Yoshimura<br>2000	*Climbing*: was assessed as climbing ≥30 flights of stairs (n=103 cases and n=103 controls).	Radiographic OA.	History of knee pain and age left school. Matched on age, sex, and district of residence.	First job:<br>- No<br>- Yes<br>Main job:<br>- No<br>- Yes	 -<br>-<br><br>-<br>-	 -<br>-<br><br>-<br>-	 -<br>-<br><br>-<br>-	 -<br>-<br><br>-<br>-	 1.0 OR<br>0.9 OR<br><br>1.0 OR<br>1.1 OR	 -<br>0.4 – 2.0<br><br>-<br>0.5 – 2.1
**Sitting**										
Allen<br>2010	*Sitting*: ever held a job requiring sitting >50 % of their time. Radiographic: no OA, exposed n=776 and OA, exposed n=356. Symptomatic: no OA, exposed n=1007 and OA, exposed n=125.	Radiographic and symptomatic OA.	Age, sex, race, BMI, smoking (ever and current vs. never), prior knee injury, and household tasks.	Radiographic OA:<br>- <50 %<br>- >50 %<br><br>Symptomatic OA:<br>- <50 %<br>- >50 %	 -<br>-<br><br><br>-<br>-	 -<br>-<br><br><br>-<br>-	 -<br>-<br><br><br>-<br>-	 -<br>-<br><br><br>-<br>-	 1.00 OR<br>0.96 OR<br><br><br>1.00 OR<br>0.80 OR	 -<br>0.79 – 1.16<br><br><br>-<br>0.61 – 1.04
	Sitting: sitting at the longest job participants held, measured on a 5-point scale and dichotomised. Radiographic: no OA, exposed n=669 and OA, exposed n=314. Symptomatic: no OA, exposed n=875 and OA, exposed n=103.	Radiographic and symptomatic OA.	Age, sex, race, BMI, smoking (ever and current vs. never), prior knee injury, and household tasks.	Radiographic OA:<br>- Never, seldom, sometimes<br>- Often or always<br><br>Symptomatic OA:	 -<br><br>-<br><br><br>-	 -<br><br>-<br><br><br>-	 -<br><br>-<br><br><br>-	 -<br><br>-<br><br><br>-	 1.00 OR<br><br>0.94 OR<br><br><br>1.00 OR	 -<br><br>0.77 – 1.14<br><br><br>-

				- Never, seldom, sometimes<br>- Often or always	-	-	-	-	0.81 OR	0.61 – 1.07
Coggon<br>1998	*Sitting*: sitting for >2 hours an average working day for up to 10 years before entry into the study (n=611 cases and n=611 controls).	Radiographic OA.	BMI, Heberden's nodes, and history of hip injury. Matched on age, sex and general practice.	- 0 years<br>- 0.1-9.9 years<br>- 10.0-19.9 years<br>- ≥20.0 years	1.0 OR<br>1.8 OR<br>1.7 OR<br>1.0 OR	-<br>0.9 – 3.7<br>0.7 – 3.9<br>0.6 – 1.7	1.0 OR<br>0.9 OR<br>1.2 OR<br>0.9 OR	-<br>0.6 – 1.3<br>0.8 – 1.8<br>0.6 – 1.3	1.0 OR<br>1.0 OR<br>1.2 OR<br>0.9 OR	-<br>0.7 – 1.4<br>0.9 – 1.8<br>0.6 – 1.2
Croft<br>1992	*Sitting*: years of exposure to sitting for >2 hours a day (n=283 referents, n=229 all cases, and n=48 severe cases).	Radiographic OA.	Age and hospital group.	All cases:<br>- <1 year<br>- 1 – 19 years<br>- ≥20 years<br>Severe cases:<br>- <1 year<br>- 1 – 19 years<br>- ≥20 years	 1.0 OR<br>1.1 OR<br>1.2 OR<br><br>1.0 OR<br>1.0 OR<br>0.8 OR	 -<br>0.7 – 1.7<br>0.8 – 1.8<br><br>-<br>0.4 – 2.2<br>0.3 – 1.7	 -<br>-<br>-<br><br>-<br>-<br>-	 -<br>-<br>-<br><br>-<br>-<br>-	 -<br>-<br>-<br><br>-<br>-<br>-	 -<br>-<br>-<br><br>-<br>-<br>-
Solovieva<br>2018	*Sitting*: was assessed from a job-exposure matrix dichotomising sitting (n=574,617 men and n=561,037 women).	Disability retirement due to OA.	Age, heavy physical work, kneeling or squatting, heavy lifting, and standing or moving.	- No<br>- Yes	1.00 HR<br>0.43 HR	-<br>0.34 – 0.54	1.00 HR<br>0.48 HR	-<br>0.39 – 0.58	-<br>-	-<br>-
Vingård<br>1997	*Sitting*: was assessed based on hours working in sitting position and divided into three subclasses based on the exposure distribution (n=230 cases and n= 273 controls).	Total hip replacement due to OA.	Age, BMI, smoking, sports activities, number of children, and hormone therapy.	- Low<br>- Medium<br>- High	-<br>-<br>-	-<br>-<br>-	1.0 RR<br>0.8 RR<br>0.8 RR	-<br>0.5 – 1.2<br>0.4 – 1.3	-<br>-<br>-	-<br>-<br>-
Yoshimura<br>2000	*Sitting*: was assessed as sitting for ≥2 hours (n=103 cases and n=103 controls).	Radiographic OA.	History of knee pain and age left school. Matched on age, sex, and district of residence.	First job:<br>- No<br>- Yes<br>Main job:<br>- No<br>- Yes	 -<br>-<br><br>-<br>-	 -<br>-<br><br>-<br>-	 -<br>-<br><br>-<br>-	 -<br>-<br><br>-<br>-	 1.0 OR<br>0.6 OR<br><br>1.0 OR<br>0.8 OR	 -<br>0.3 – 1.0<br><br>-<br>0.4 – 1.4
**Combined mechanical exposures**										
Allen<br>2010	*Light work*: ever held a job requiring light work while standing >50% of their time Radiographic: no OA, exposed n=422 and OA, exposed n=202. Symptomatic: no OA, exposed n=560 and OA, exposed n=63.	Radiographic and symptomatic OA.	Age, sex, race, BMI, smoking (ever and current vs. never), prior knee injury, and household tasks.	Radiographic OA:<br>- <50 %<br>- >50 %<br><br>Symptomatic OA:<br>- <50 %<br>- >50 %	 -<br>-<br><br><br>-<br>-	 -<br>-<br><br><br>-<br>-	 -<br>-<br><br><br>-<br>-	 -<br>-<br><br><br>-<br>-	 1.00 OR<br>1.05 OR<br><br><br>1.00 OR<br>0.74 OR	 -<br>0.85 – 1.29<br><br><br>-<br>0.54 – 1.01
	Heavy work: ever held a job requiring heavy work while standing >50% of their time.	Radiographic and symptomatic OA.	Age, sex, race, BMI, smoking (ever and current vs. never), prior	Radiographic OA:<br>- <50 %<br>- >50 %	 -<br>-	 -<br>-	 -<br>-	 -<br>-	 1.00 OR<br>1.04 OR	 -<br>0.82 – 1.32

	Radiographic: no OA, exposed n=328 and OA, exposed n=144.<br>Symptomatic: no OA, exposed n=405 and OA, exposed n=65.		knee injury, and household tasks.	Symptomatic OA:<br>- <50 %<br>- >50 %	 -<br>-	 -<br>-	 -<br>-	 -<br>-	 1.00 OR<br>1.39 OR	 -<br>1.01 – 1.91
	Heavy work: doing heavy work while standing at the longest job participants held, measured on a 5-point scale and dichotomised.<br>Radiographic: no OA, exposed n=180 and OA, exposed n=84.<br>Symptomatic: no OA, exposed n=222 and OA, exposed n=41.	Radiographic and symptomatic OA.	Age, sex, race, BMI, smoking (ever and current vs. never), prior knee injury, and household tasks.	Radiographic OA:<br>- Never, seldom, sometimes<br>- Often or always<br><br>Symptomatic OA:<br>- Never, seldom, sometimes<br>- Often or always	 -<br><br>-<br><br><br>-<br><br>-	 -<br><br>-<br><br><br>-<br><br>-	 -<br><br>-<br><br><br>-<br><br>-	 -<br><br>-<br><br><br>-<br><br>-	 1.00 OR<br><br>1.20 OR<br><br><br>1.00 OR<br><br>1.75 OR	 -<br><br>0.88 – 1.63<br><br><br>-<br><br>1.17 – 2.61
Cvijetic<br>1999	*Physical demands at work*: was divided into 4 categories depending of the physical demands pertinent to participants' occupation.<br>Category 1, n=119 men and 96 women<br>Category 2, n=34 men and 38 women<br>Category 3, n=92 men and 115 women<br>Category 4, n=50 men and 49 women.	Radiographic OA.	Age and BMI.	Radiological signs:<br>- Category 1<br>- Category 2<br>- Category 3<br>- Category 4<br>Clinical signs:<br>- Category 1<br>- Category 2<br>- Category 3<br>- Category 4	 1.00 OR<br>1.50 OR<br>1.16 OR<br>1.15 OR<br><br>1.00 OR<br>2.20 OR<br>1.40 OR<br>2.19 OR	 -<br>0.60 – 3.21<br>0.58 – 2.30<br>0.52 – 2.52<br><br>-<br>0.30 – 13.1<br>0.50 – 4.00<br>0.44 – 10.8	 1.00 OR<br>1.45 OR<br>1.19 OR<br>1.34 OR<br><br>1.00 OR<br>3.00 OR<br>1.37 OR<br>1.77 OR	 -<br>0.49 – 3.58<br>0.65 – 2.32<br>0.52 – 3.04<br><br>-<br>1.00 – 8.92<br>0.50 – 3.79<br>0.36 – 8.63	 -<br>-<br>-<br>-<br><br>-<br>-<br>-<br>-	 -<br>-<br>-<br>-<br><br>-<br>-<br>-<br>-
	Physical demands at work: years exposed to physical demands at work within occupation groups (n=NS in exposure groups).	Radiographic OA.	Age and BMI.	Category 1:<br>- <20 years<br>- 20-29.9 years<br>- >30 years<br>Category 2:<br>- <20 years<br>- 20-29.9 years<br>- >30 years<br>Category 3:<br>- <20 years<br>- 20-29.9 years<br>- >30 years<br>Category 4:<br>- <20 years<br>- 20-29.9 years<br>- >30 years	 1.00 OR<br>1.38 OR<br>1.49 OR<br><br>1.00 OR<br>2.09 OR<br>1.83 OR<br><br>1.00 OR<br>2.45 OR<br>2.46 OR<br><br>1.00 OR<br>1.57 OR<br>1.22 OR	 -<br>0.94 – 2.79<br>0.79 – 3.20<br><br>-<br>0.98 – 3.17<br>0.68 – 3.56<br><br>-<br>0.53 – 3.32<br>0.51 – 4.54<br><br>-<br>0.64 – 2.85<br>0.98 – 2.46	 1.00 OR<br>1.07 OR<br>3.89 OR<br><br>1.00 OR<br>1.94 OR<br>3.24 OR<br><br>1.00 OR<br>1.51 OR<br>2.34 OR<br><br>1.00 OR<br>1.23 OR<br>1.41 OR	 -<br>1.03 – 1.29<br>0.42 – 4.64<br><br>-<br>0.64 – 2.14<br>0.51 – 4.02<br><br>-<br>0.68 – 1.92<br>0.66 – 2.93<br><br>-<br>0.97 – 1.54<br>0.78 – 1.97	 -<br>-<br>-<br><br>-<br>-<br>-<br><br>-<br>-<br>-<br><br>-<br>-<br>-	 -<br>-<br>-<br><br>-<br>-<br>-<br><br>-<br>-<br>-<br><br>-<br>-<br>-
Flugsrud<br>2002	*Physical activity at work*: was divided into categories of from sedentary work to heavy physical labour (n= 24884 men and n=24874 women).	Register data on total hip replacements.	Age at screening, height, BMI, physical activity in leisure, marital status, and smoking habits.	- Sedentary<br>- Moderate<br>- Intermediate<br>- Intensive	1.0 RR<br>1.5 RR<br>1.7 RR<br>2.1 RR	-<br>1.0 – 2.2<br>1.1 – 2.4<br>1.5 – 3.0	1.0 RR<br>1.1 RR<br>1.4 RR<br>2.1 RR	-<br>0.8 – 1.6<br>0.9 – 2.0<br>1.3 – 3.3	-<br>-<br>-<br>-	-<br>-<br>-<br>-

Heliovaara<br>1993	*Physical stress at work*: a sum index of 5 occupational mechanical exposures (lifting/carrying, twisted/awkward posture, whole body vibration, repeated movement, and working speed) (n=7217)	Clinical diagnosis.	BMI, injury to lower limb, sex, and age.	Unilateral OA:<br>- 0<br>- 1<br>- 2<br>- 3<br>- 4–5<br>Bilateral OA:<br>- 0<br>- 1<br>- 2<br>- 3<br>- 4–5<br>Uni or bilateral OA:<br>- 0<br>- 1<br>- 2<br>- 3<br>- 4–5	 -<br>-<br>-<br>-<br>-<br><br>-<br>-<br>-<br>-<br>-<br><br><br>-<br>-<br>-<br>-<br>-	 -<br>-<br>-<br>-<br>-<br><br>-<br>-<br>-<br>-<br>-<br><br><br>-<br>-<br>-<br>-<br>-	 -<br>-<br>-<br>-<br>-<br><br>-<br>-<br>-<br>-<br>-<br><br><br>-<br>-<br>-<br>-<br>-	 -<br>-<br>-<br>-<br>-<br><br>-<br>-<br>-<br>-<br>-<br><br><br>-<br>-<br>-<br>-<br>-	 1.0 OR<br>1.1 OR<br>1.5 OR<br>2.4 OR<br>2.3 OR<br><br>1.0 OR<br>1.4 OR<br>2.2 OR<br>2.8 OR<br>2.9 OR<br><br><br>1.0 OR<br>1.2 OR<br>1.9 OR<br>2.7 OR<br>2.7 OR	 -<br>0.7 – 1.8<br>1.0 – 2.3<br>1.4 – 3.8<br>1.2 – 4.3<br><br>-<br>0.8 – 2.3<br>1.5 – 3.4<br>1.6 – 4.7<br>1.5 – 5.8<br><br><br>-<br>0.9 – 1.8<br>1.4 – 2.6<br>1.8 – 3.9<br>1.7 – 4.4
Jacobsson<br>1987	*Heavy labour*: was assessed as subject to heavy labour, particularly to farming, forestry, industrial work or heavy lifting, or to much walking, standing or tractor driving (n=342)	Radiographic OA.	None.	- No<br>- Yes	1.00 OR<br>2.42 OR	-<br>1.33 – 4.41	-<br>-	-<br>-	-<br>-	-<br>-
Juhakoski<br>2009	*Physical work load*: were assessed as categories of physical workload ranking the exposure from least to heaviest (n=840).	Clinical examination.	Age, sex, education, BMI, smoking, alcohol intake, leisure time physical activity, and injury.	- Light sedentary<br>- Other sedentary<br>- Light standing<br>- Medium heavy<br>- Heavy manual	-<br>-<br>-<br>-<br>-	-<br>-<br>-<br>-<br>-	-<br>-<br>-<br>-<br>-	-<br>-<br>-<br>-<br>-	1.0 OR<br>1.1 OR<br>1.2 OR<br>3.1 OR<br>6.7 OR	-<br>0.1 – 10.0<br>0.4 – 3.4<br>1.2 – 8.0<br>2.3 – 19.5
Kontio<br>2020	*Manual handling of heavy loads*: was assessed as cumulative exposure to lifting, carrying or pushing ≥20 kg for ≥10 times a day (n=4405 with no OA and n=87 for hip OA).	Register information on hip OA.	Age and sex.	- <1 year<br>- 1-10 years<br>- 11-20 years<br>- >20 years	-<br>-<br>-<br>-	-<br>-<br>-<br>-	-<br>-<br>-<br>-	-<br>-<br>-<br>-	1.00 HR<br>1.34 HR<br>0.41 HR<br>0.64 HR	-<br>0.77 – 2.34<br>0.13 – 1.30<br>0.31 – 1.30
	Composite cumulative work load: was calculated using k-means cluster analysis to capture duration and pattern of co-occurrence of physically heavy work, manual handling, kneeling or squatting, and standing or walking (n=4405 with no OA and n=87 for hip OA).	Register information on hip OA.	Age, sex, prior injury, and BMI.	- Low<br>- Intermediate<br>- High	-<br>-<br>-	-<br>-<br>-	-<br>-<br>-	-<br>-<br>-	1.00 HR<br>1.19 HR<br>1.28 HR	-<br>0.74 – 1.93<br>0.68 – 2.39

	Physically heavy work: was assessed as cumulative exposure to work involving lifting and carrying heavy loads, excavating, shovelling or hammering (n=4405 with no OA and n=87 for hip OA).	Register information on hip OA.	Age and sex.	- <1 year<br>- 1-10 years<br>- 11-20 years<br>- >20 years	-<br>-<br>-<br>-	-<br>-<br>-<br>-	-<br>-<br>-<br>-	-<br>-<br>-<br>-	1.00 HR<br>1.01 HR<br>1.40 HR<br>1.06 HR	-<br>0.53 – 1.92<br>0.75 – 2.61<br>0.61 – 1.84
	Cumulative exposure to physical workload: was calculated as the total number of years having been exposed during the entire work career for each workload factor (n=4405 with no OA and n=87 for hip OA).	Register information on hip OA.	Age and sex.	- Low<br>- Intermediate<br>- High	-<br>-<br>-	-<br>-<br>-	-<br>-<br>-	-<br>-<br>-	1.00 HR<br>1.18 HR<br>1.34 HR	-<br>0.74 – 1.88<br>0.72 – 2.48
Ratzlaff<br>2011	*Cumulative peak force index*: was measured using time spend in specific occupational activities, bodyweight, and the peak hip joint force for each activity (%bodyweight), and divided into quintiles (n= 2918)	Self-reporting hip OA.	Sex, previous injury, age, sport/recreation, and household.	- Index 1<br>- Index 2<br>- Index 3<br>- Index 4<br>- Index 5	-<br>-<br>-<br>-<br>-	-<br>-<br>-<br>-<br>-	-<br>-<br>-<br>-<br>-	-<br>-<br>-<br>-<br>-	1.00 HR<br>1.11 HR<br>1.30 HR<br>1.58 HR<br>1.80 HR	-<br>0.63 – 1.83<br>0.72 – 2.11<br>0.86 – 2.52<br>0.95 – 2.82
Rijs<br>2014	*Use of force*: was assessed using a job-exposure matrix measuring use of force from lifting, pushing, pulling, carrying or use of tools (n=272 for current job and n= 972 for longest job held).	Self-reporting and general practitioner data.	Age and sex.	Current job:<br>- Low<br>- Moderate<br>Longest job:<br>- Low<br>- Moderate	 -<br>-<br><br>-<br>-	 -<br>-<br><br>-<br>-	 -<br>-<br><br>-<br>-	 -<br>-<br><br>-<br>-	 1.0 OR<br>2.5 OR<br><br>1.0 OR<br>1.5 OR	 -<br>0.9 – 7.5<br><br>-<br>0.99 – 2.4
Riyazi<br>2008	*Physically demanding jobs*: was based on expert judgments of job titles containing demanding work characterised by lifting of heavy objects, handling of heavy tools, stooping, frequently in combination with standing or walking (n=382 cases and n=345 controls).	Radiographic OA.	Age, sex, and BMI.	- No<br>- Yes	-<br>-	-<br>-	-<br>-	-<br>-	1.0 OR<br>3.3 OR	-<br>1.3 - 8.2
Roach<br>1994	*Workload*: was assessed as number of years exposed to mechanical exposures classified into three categories (n=99 cases and n=233 controls).	Radiographic and register data.	None.	- Light<br>- Intermediate<br>- Heavy	1.0 OR<br>1.9 OR<br>2.4 OR	-<br>1.0 – 3.8<br>1.3 – 4.3	-<br>-<br>-	-<br>-<br>-	-<br>-<br>-	-<br>-<br>-
	Workload: was assessed as number of years exposed to mechanical exposures classified	Radiographic and register data.	Cancer, obesity at age 40, and football.	- Light<br>- Heavy	1.0 OR<br>2.4 OR	-<br>1.2 – 4.7	-<br>-	-<br>-	-<br>-	-<br>-

	into three categories (n=99 cases and n=233 controls).									
Rubak<br>2013**	*Physical workloads*: was assessed as exposure to overall physical workload to the hip, calculated as number of employment years, based on total lifts per working day, frequency of lifting ≥20 kg, whole-body vibration, and standing/walking the majority of the day (n= 1010944 men and 899549 women).	Register information on total hip replacement.	Age, cumulative physical workload, calendar year, county of residence, and socioeconomic status.	- 0<br>- >0 - <5 point-years<br>- 5 - <15 point-years<br>- 15-<25 point-years<br>- 25-<35 point-years<br>- 35 - 86 point-years	1.00 OR<br>1.13 OR<br>1.14 OR<br>1.19 OR<br>1.27 OR<br>1.33 OR	-<br>0.98 – 1.31<br>1.00 – 1.31<br>1.04 – 1.36<br>1.11 – 1.48<br>1.17 – 1.53	1.00 OR<br>0.96 OR<br>0.96 OR<br>0.94 OR<br>0.99 OR<br>1.01 OR	-<br>0.8 – 1.06<br>0.87 – 1.05<br>0.85 – 1.04<br>0.88 – 1.10<br>0.88 – 1.16	-<br>-<br>-<br>-<br>-<br>-	-<br>-<br>-<br>-<br>-<br>-
Solovieva<br>2018	*Heavy physical work*: was assessed from a job-exposure matrix dichotomising heavy physical work (n=574,617 men and n=561,037 women).	Register information on disability retirement due to OA.	Age, kneeling or squatting, heavy lifting, sitting, and standing or moving.	- No<br>- Yes	1.00 HR<br>1.34 HR	-<br>1.10 – 1.64	1.00 HR<br>1.65 HR	-<br>1.39 – 1.95	-<br>-	-<br>-
Thelin<br>1997	*Heavy physical work*: was assessed as subjective heavy physical work for more than half a year before participants were 16 years of age (n=216 cases and n=479 controls).	Radiographic OA.	None. Matched on age and place of residence.	- No<br>- Yes	1.00 OR<br>2.06 OR	-<br>1.48 – 2.86	-<br>-	-<br>-	-<br>-	-<br>-
Vingård<br>1991	*Dynamic*: was assessed as exposure to walking with burdens and stair climbing and divided into three subclasses on the basis of the exposure distribution (n=233 cases and n=302 controls).	Total hip replacement due to OA.	Age, BMI, smoking, and sport activities up to the age of 29 years.	- Low<br>- Medium<br>- High	1.00 RR<br>1.92 RR<br>2.17 RR	-<br>1.11 – 3.32<br>1.27 – 3.73	-<br>-<br>-	-<br>-<br>-	-<br>-<br>-	-<br>-<br>-
	Static + dynamic: was assessed as exposure to working in a twisted locked position, walking with burdens, and stair climbing and divided into three subclasses based on the exposure distribution (n=233 cases and n=302 controls).	Total hip replacement due to OA.	Age, BMI, smoking, and sport activities up to the age of 29 years.	- Low<br>- Medium<br>- High	1.00 RR<br>1.82 RR<br>2.42 RR	-<br>1.02 – 3.24<br>1.45 – 4.04	-<br>-<br>-	-<br>-<br>-	-<br>-<br>-	-<br>-<br>-
**Other occupational mechanical exposures**										
Allen<br>2010	*Crawl*: crawling on knees at the longest job participants held, measured on a 5-point scale.	Hip OA.	Age, sex, race, BMI, smoking (ever and current vs. never), prior	Radiographic OA:<br>- Never, seldom, sometimes<br>- Often or always	-<br>-	-<br>-	-<br>-	-<br>-	1.00 OR<br>1.35 OR	-<br>0.93 – 2.00

	Radiographic: no OA, exposed n=107 and OA, exposed n=60. Symptomatic: no OA, exposed n=137 and OA, exposed n=30.		knee injury, and household tasks.	Symptomatic OA:<br>- Never, seldom, sometimes<br>- Often or always	-<br>-	-<br>-	-<br>-	-<br>-	1.00 OR<br>2.28 OR	-<br>1.43 – 3.65
Coggon<br>1998	*Driving*: driving for >4 hours in an average working day for up to 10 years before entry into the study (n=611 cases and n=611 controls).	Radiographic OA.	BMI, Heberden's nodes, and history of hip injury. Matched on age, sex, and general practice.	- 0 years<br>- 0.1-9.9 years<br>- 10.0-19.9 years<br>- ≥20.0 years	1.0 OR<br>1.3 OR<br>0.5 OR<br>0.9 OR	-<br>0.7 – 2.6<br>0.2 – 1.3<br>0.4 – 1.8	1.0 OR<br>4.0 OR<br>2.7 OR<br>-	-<br>1.2 – 13.7<br>0.3 – 28.5	1.0 OR<br>1.8 OR<br>0.7 OR<br>1.0 OR	-<br>1.0 – 3.1<br>0.3 – 1.5<br>0.5 – 1.9
Croft<br>1992	*Driving*: years of exposure to driving for >4 hours a day (n=274 referents, n=228 all cases, and n=47 severe cases).	Radiographic OA.	Age and hospital group.	All cases:<br>- <1 year<br>- ≥1 year<br>Severe cases:<br>- <1 year<br>- ≥1 year	1.0 OR<br>0.8 OR<br>1.0 OR<br>0.9 OR	-<br>0.5 – 1.2<br>-<br>0.4 – 1.8	-<br>-<br>-<br>-	-<br>-<br>-<br>-	-<br>-<br>-<br>-	-<br>-<br>-<br>-
	Running: years of running for >1 hour a day (n=269 referents, n=228 all cases, and n=47 severe cases).	Radiographic OA.	Age and hospital group.	All cases:<br>- <1 year<br>- ≥1 year<br>Severe cases:<br>- <1 year<br>- ≥1 year	1.0 OR<br>1.3 OR<br>1.0 OR<br>0.5 OR	-<br>0.5 – 3.3<br>-<br>0.1 – 4.2	-<br>-<br>-<br>-	-<br>-<br>-<br>-	-<br>-<br>-<br>-	-<br>-<br>-<br>-
Lau<br>2000	*Digging*: was assessed as digging for ≥1 hour on an average working day (n=30 cases and n=90 controls for men and n=108 cases and n=324 controls for women).	Radiographic OA.	Men: none. Matched on age.<br><br>Women: adjusted for height, weight, history of joint injury, and regular sports activities. Matched on age.	- No<br>- Yes	1.0 OR<br>2.0 OR	-<br>0.3 – 12.0	1.0 OR<br>2.2 OR	-<br>0.8 – 6.5	-<br>-	-<br>-
	Driving: was assessed as driving for ≥4 hour on an average working day (n=30 cases and n=90 controls for men and n=108 cases and n=324 controls for women).	Radiographic OA.	Men and women: None. Matched on age.	- No<br>- Yes	1.0 OR<br>0.4 OR	-<br>0.04 – 3.0	-<br>-	-<br>-	-<br>-	-<br>-
	Vibrations: was assessed as use of vibration tools for ≥1 hour each day (n=108 cases and n=324 controls).	Radiographic OA.	Height, weight, history of joint injury, and regular sports activity. Matched on age.	- No<br>- Yes	-<br>-	-<br>-	1.0 OR<br>7.9 OR	-<br>0.8 – 77.8	-<br>-	-<br>-
	Vibrations: was assessed as use of vibration tools for ≥1 hour each day (n=30 cases and n=90 controls)	Radiographic OA.	None. Matched on age.	- No<br>- Yes	1.0 OR<br>0.7 OR	-<br>0.2 – 2.3	-<br>-	-<br>-	-<br>-	-<br>-

Lau<br>2007	*Digging*: was assessed as digging for ≥1 hour on an average working day (n=30 cases and n=90 controls for men and n=108 cases and n=324 controls for women).	Radiographic OA.	BMI and injury. Matched on age.	- No<br>- Yes	1.00 OR<br>1.98 OR	-<br>0.21 -19.07	1.00 OR<br>3.25 OR	-<br>1.47 – 7.20	1.00 OR<br>2.92 OR	-<br>1.40 – 6.11
	Driving: was assessed as driving for ≥4 hour on an average working day (n=30 cases and n=90 controls for men and n=108 cases and n=324 controls for women).	Radiographic OA.	BMI and injury. Matched on age.	- No<br>- Yes	1.00 OR<br>0.30 OR	-<br>0.03 – 3.09	-<br>-	-<br>-	-<br>-	-<br>-
	Vibrations: was assessed as use of vibration tools for an hour each day (n=30 cases and n=90 controls for men and n=108 cases and n=324 controls for women)	Radiographic OA.	BMI and injury. Matched on age.	- No<br>- Yes	1.00 OR<br>2.15 OR	-<br>0.36 –<br>12.73	1.00 OR<br>7.68 OR	-<br>1.32 –<br>44.80	1.00 OR<br>3.94 OR	-<br>1.18 – 13.12
Olsen<br>1994	*Jumping*: No information on the collection was provided, but the exposure was aggregated for men's work life up to 49th year of age. It was then defined into three exposure groups (n=239 cases and n=302 controls).	First-time prosthesis.	Age, BMI, smoking and sports activities.	- Low<br>- Medium<br>- High	1.00 OR<br>1.83 OR<br>1.52 OR		-<br>-<br>-	-<br>-<br>-	-<br>-<br>-	-<br>-<br>-
Rijs<br>2014	*Repetitive movements*: was assessed with a job-exposure matrix measuring repetitive movements at work (n=268 for current job and n=820 for longest job held).	Hip OA.	Age and sex.	Current job:<br>- Low<br>- Moderate<br>- High<br>Longest job:<br>- Low<br>- Moderate<br>- High	 -<br>-<br>-<br><br>-<br>-<br>-	 -<br>-<br>-<br><br>-<br>-<br>-	 -<br>-<br>-<br><br>-<br>-<br>-	 -<br>-<br>-<br><br>-<br>-<br>-	 1.0 OR<br>5.7 OR<br>6.2 OR<br><br>1.0 OR<br>2.1 OR<br>2.5 OR	 -<br>0.7 – 45.6<br>0.7 – 56.4<br><br>-<br>0.99 – 4.6<br>1.2 – 5.6
Rubak<br>2014	*Whole-body vibrations*: was assessed as ever or never exposure to whole-body vibrations (n=1776 case-control sets including at least 1 case and 1 control, divided in 861 sets for women and 915 sets for men).	Total hip replacement due to OA.	One occupational exposure at a time adjusted for body mass index at age 25, change in body mass index, pack-years of smoking, previous fracture of a lower extremity, familial predisposition, endurance and contact sport at age 25, and region of residence. Matched on age.	- Never<br>- Ever	1.00 OR<br>1.26 OR	-<br>0.97 – 1.64	1.00 OR<br>0.64 OR	-<br>0.35 – 1.15	-<br>-	-<br>-

Vingård<br>1997	*Jumps*: was assessed based on numbers of jumps or movements between different levels on work and divided into three subclasses based on the exposure distribution (n=503).	Total hip replacement due to OA.	Age, BMI, smoking, sports activities, number of children, and hormone therapy.	- Low<br>- Medium<br>- High	-<br>-<br>-	-<br>-<br>-	1.0 RR<br>1.0 RR<br>2.1 RR	-<br>0.5 – 2.0<br>1.1 – 4.2	-<br>-<br>-	-<br>-<br>-
Yoshimura<br>2000	*Driving*: was assessed as driving for ≥4 hours (n=103 cases and n=103 controls).	Radiographic OA.	History of knee pain and age left school. Matched on age, sex, and district of residence.	First job:<br>- No<br>- Yes<br>Main job:<br>- No<br>- Yes	 -<br>-<br><br>-<br>-	 -<br>-<br><br>-<br>-	 -<br>-<br><br>-<br>-	 -<br>-<br><br>-<br>-	 1.0 OR<br>1.1 OR<br><br>1.0 OR<br>1.4 OR	 -<br>0.1 – 7.6<br><br>-<br>0.4 – 5.4

Abbreviations: BMI = body mass index; HR = hazard ratio; n = numbers; OA = osteoarthritis; OR = odds ratio; RR = relative risk;
** In Rubak 2013, numbers in table 2 are not consistent with numbers provided in flow chart (a total of 9 women). We have extracted numbers of participants from table 2 used in the forest plot.

Appendix 7. Funnel plots

Figure 2. Funnel plot of all included exposure categories.

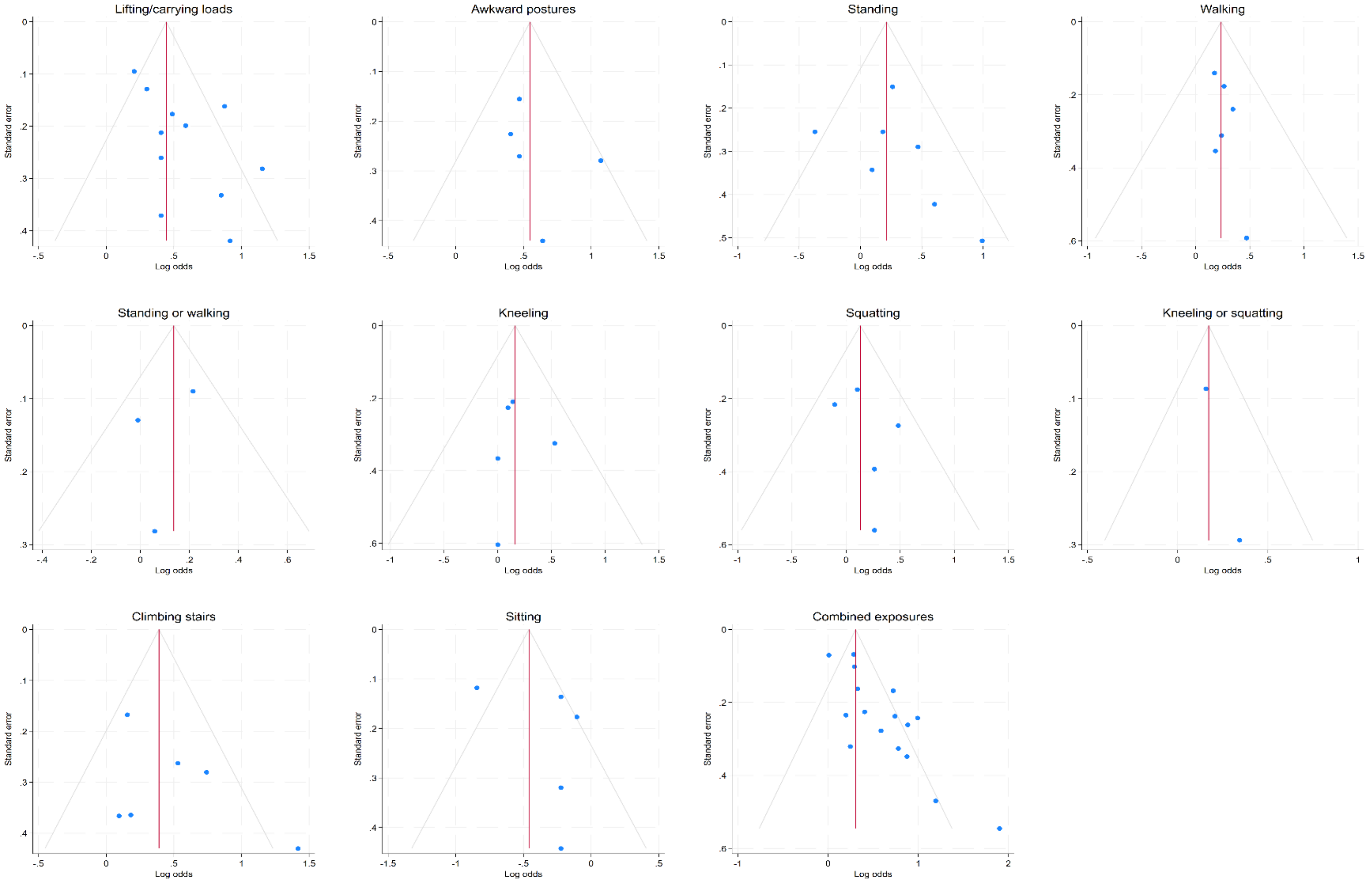

Appendix 8. Scatter plots

Figure 3. Lifting/carrying loads

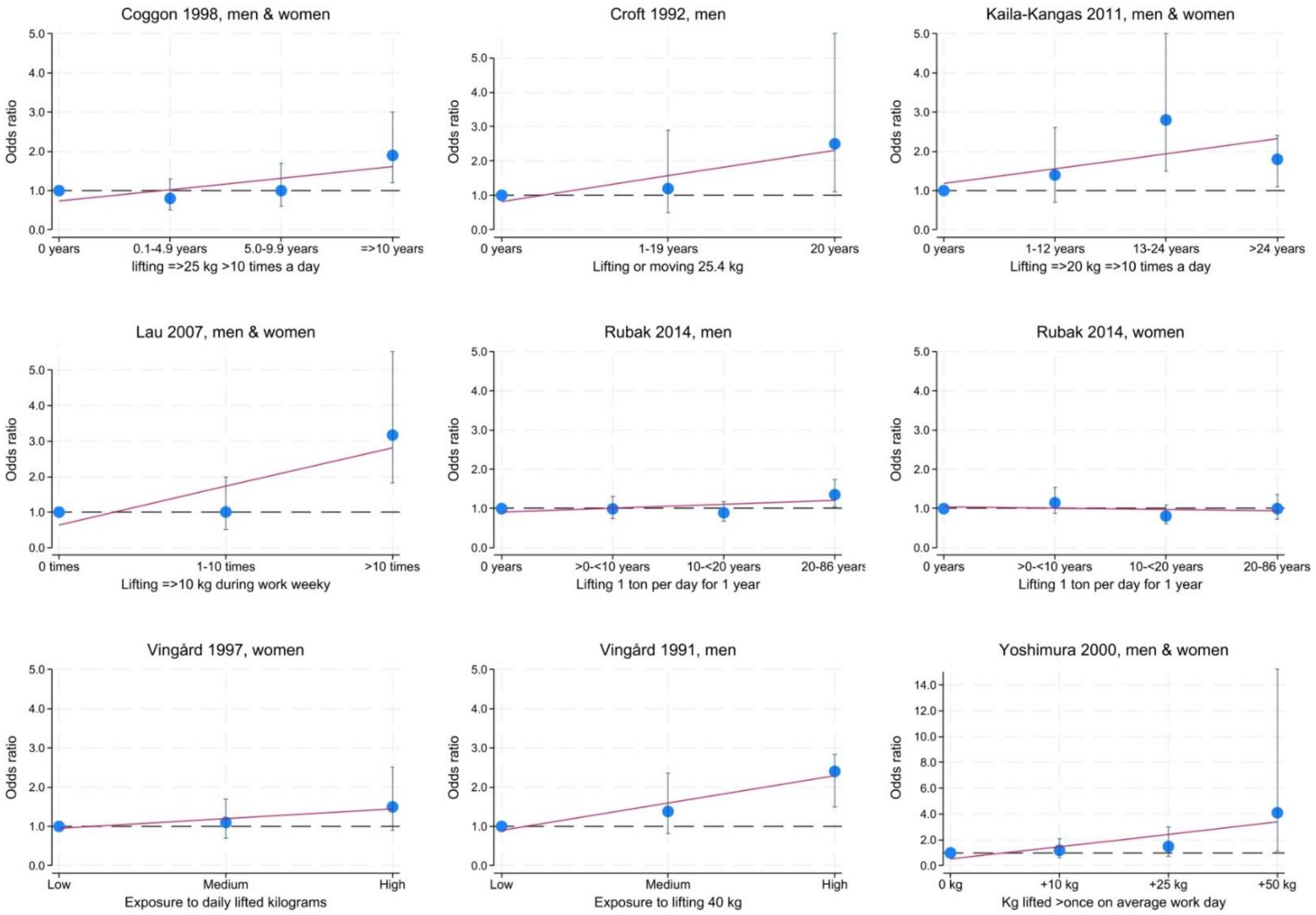

Figure 4. Standing.

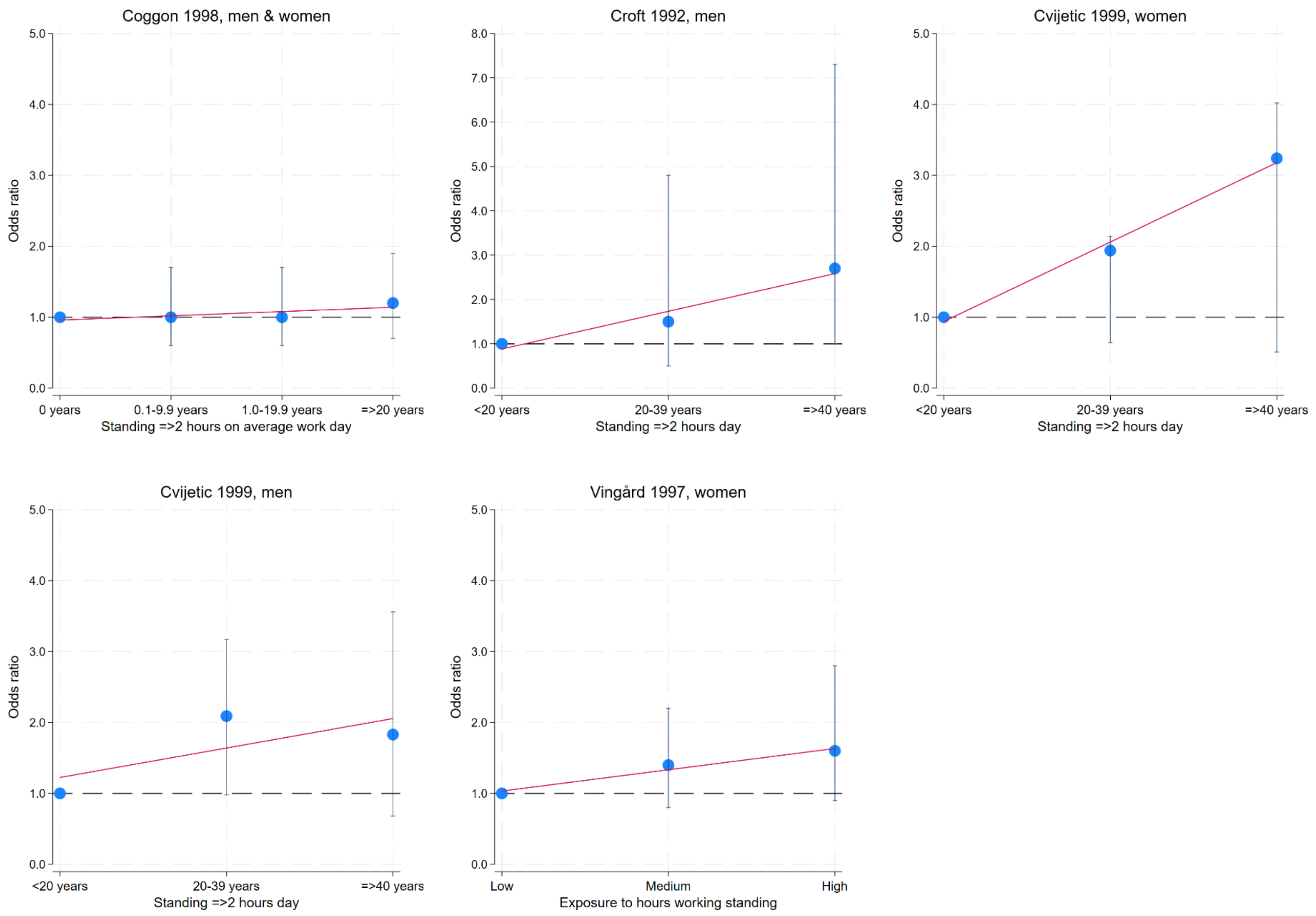

Figure 5. Walking.

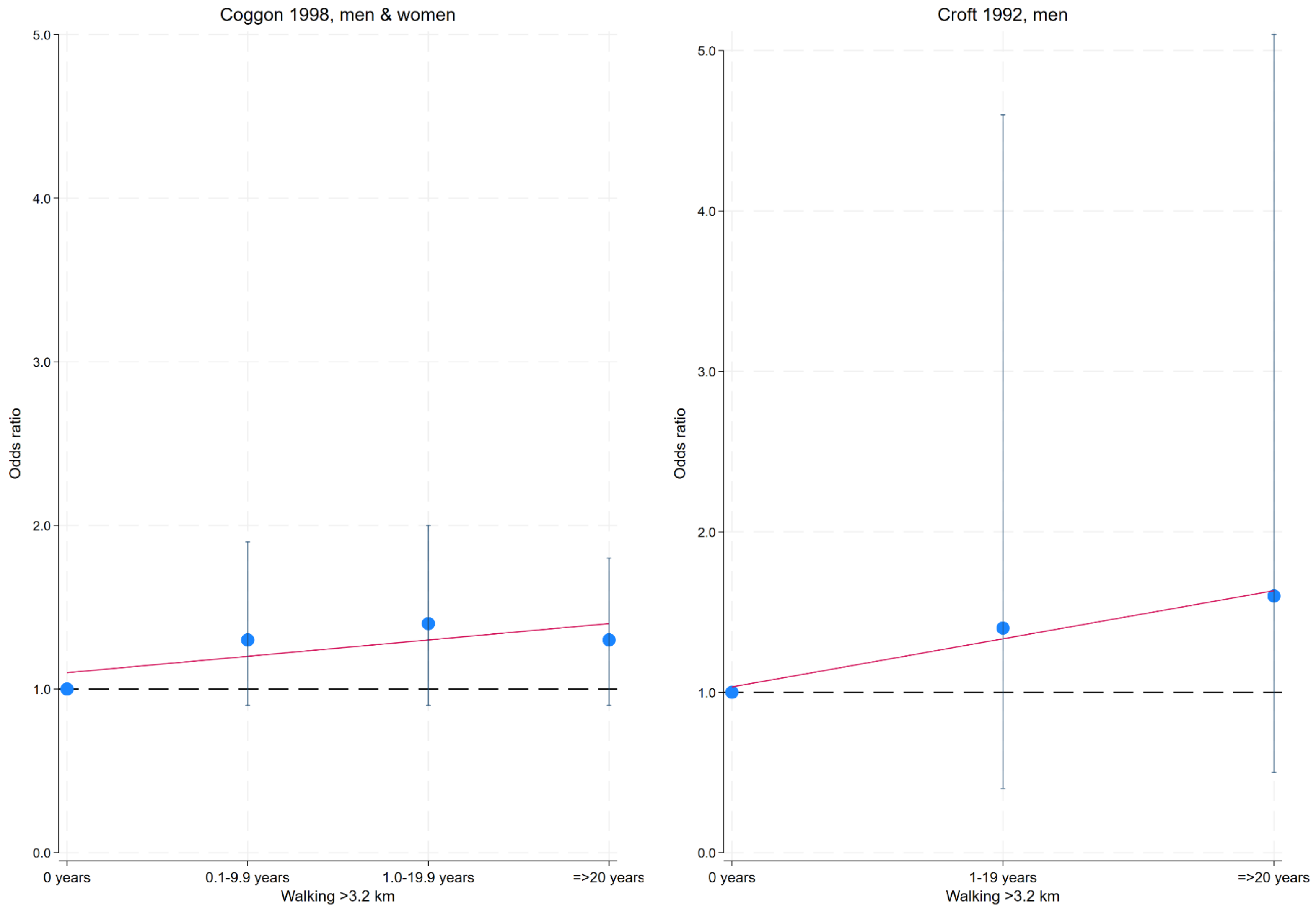

Figure 6. Climbing stairs

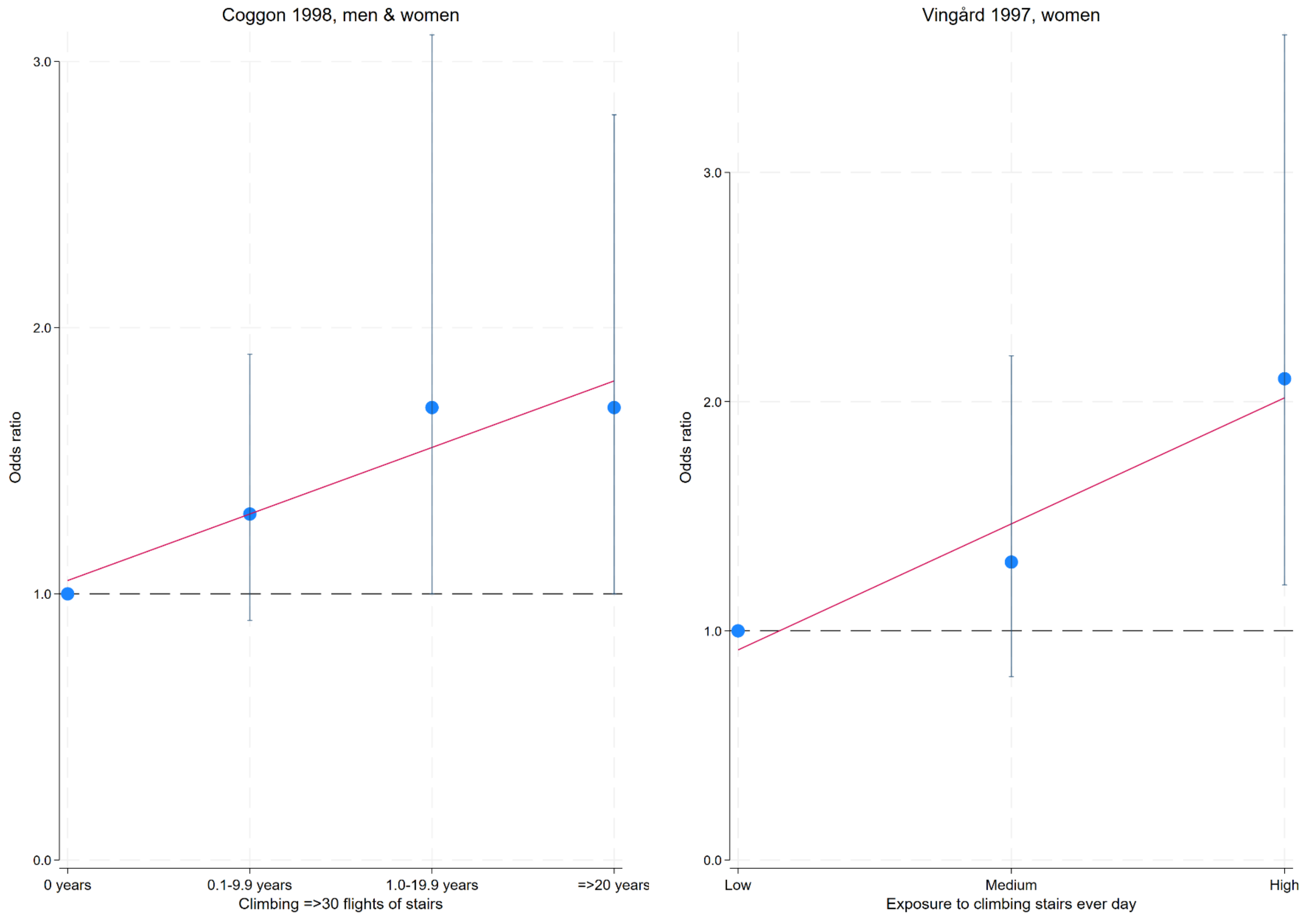

Figure 7. Non-neutral postures

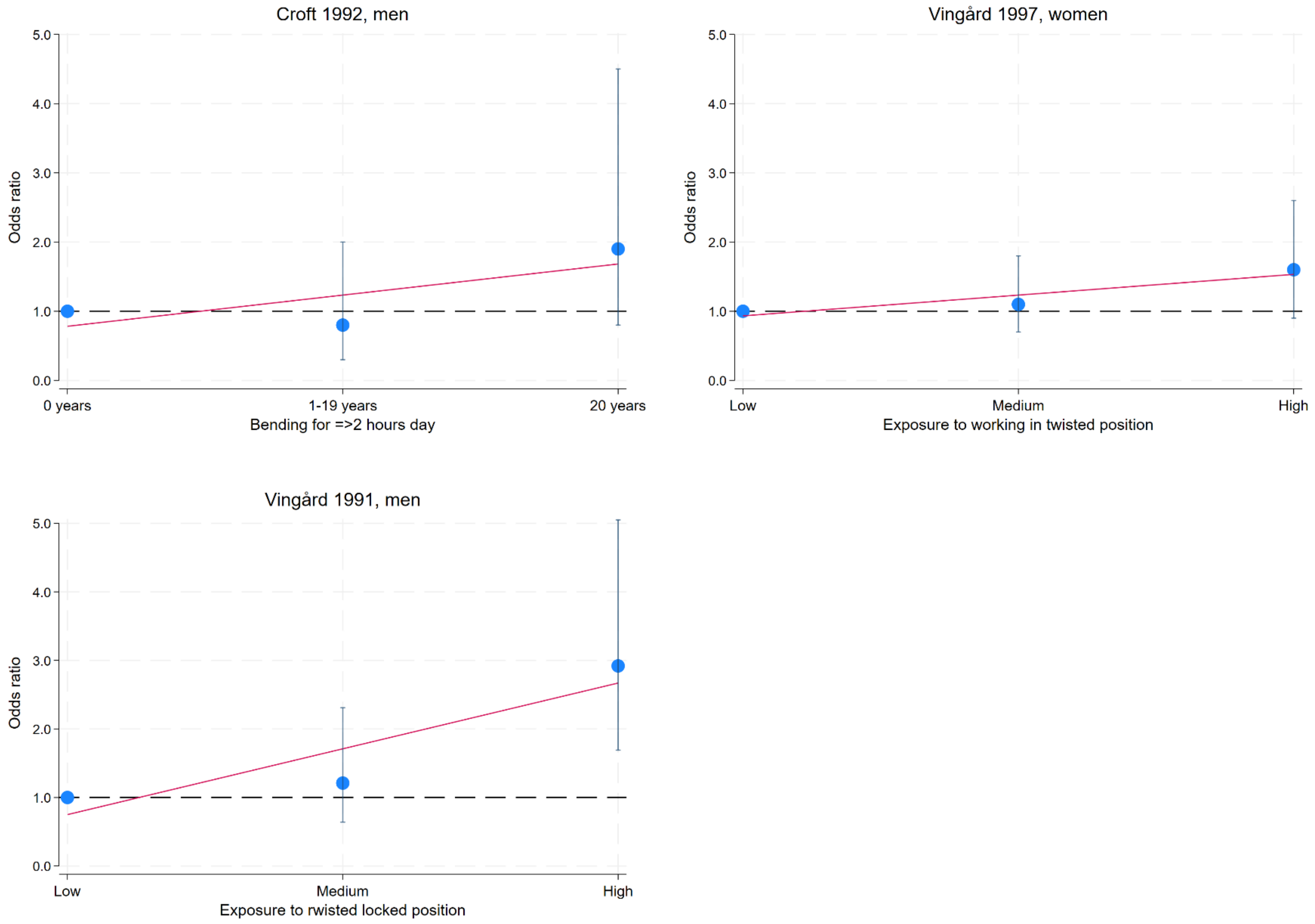

Figure 8. Sitting

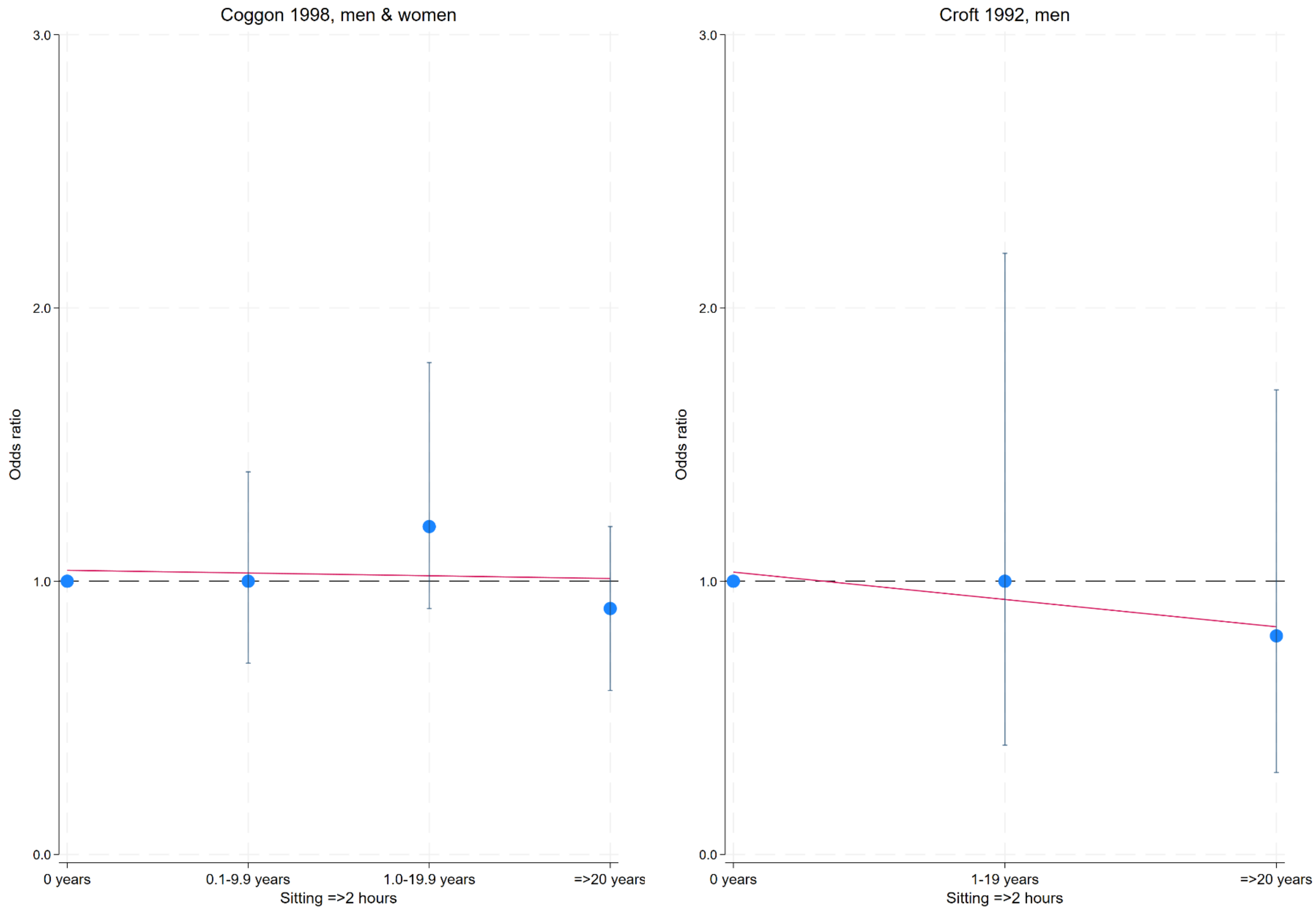

Figure 9. Kneeling

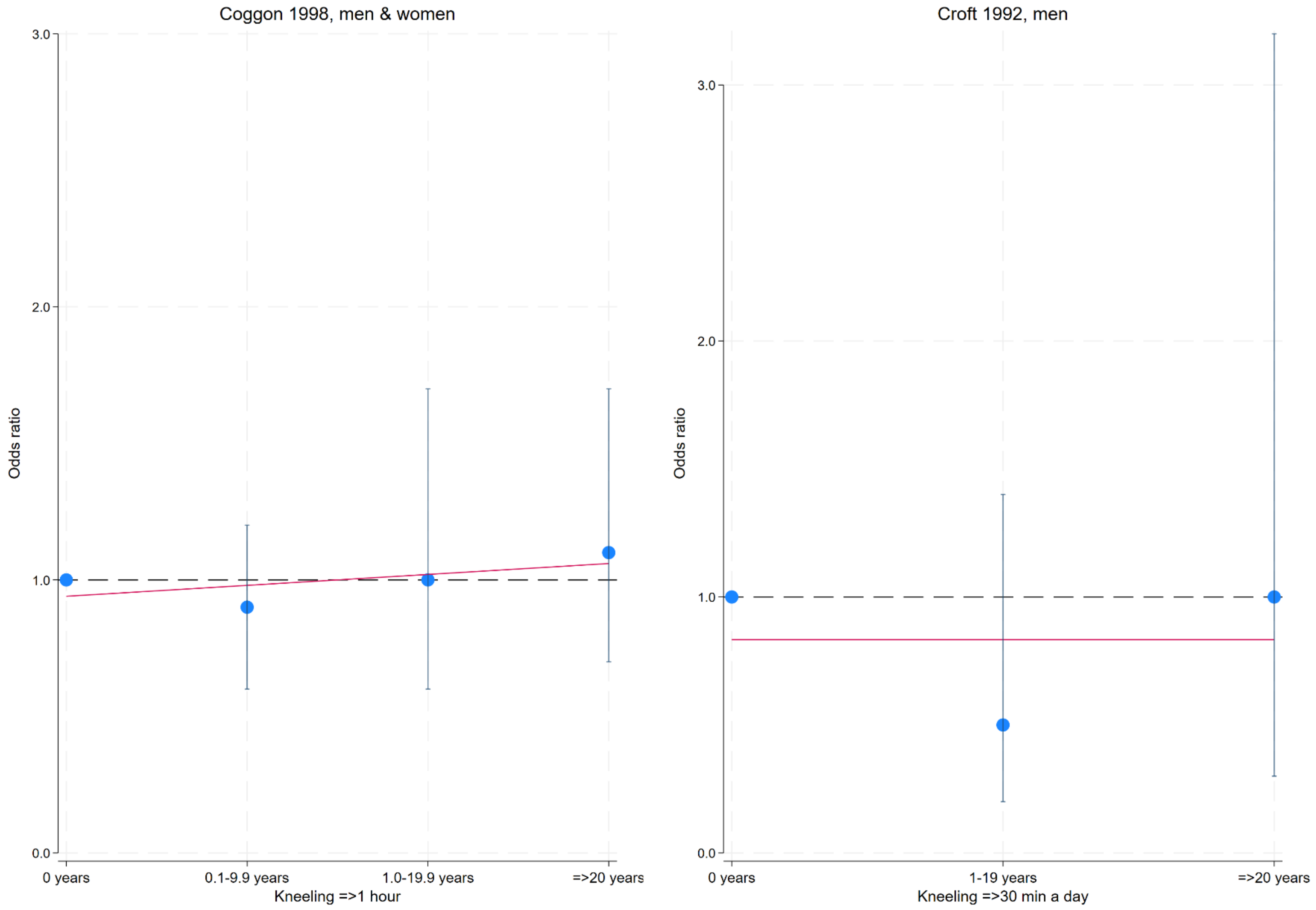

Figure 10. Squatting

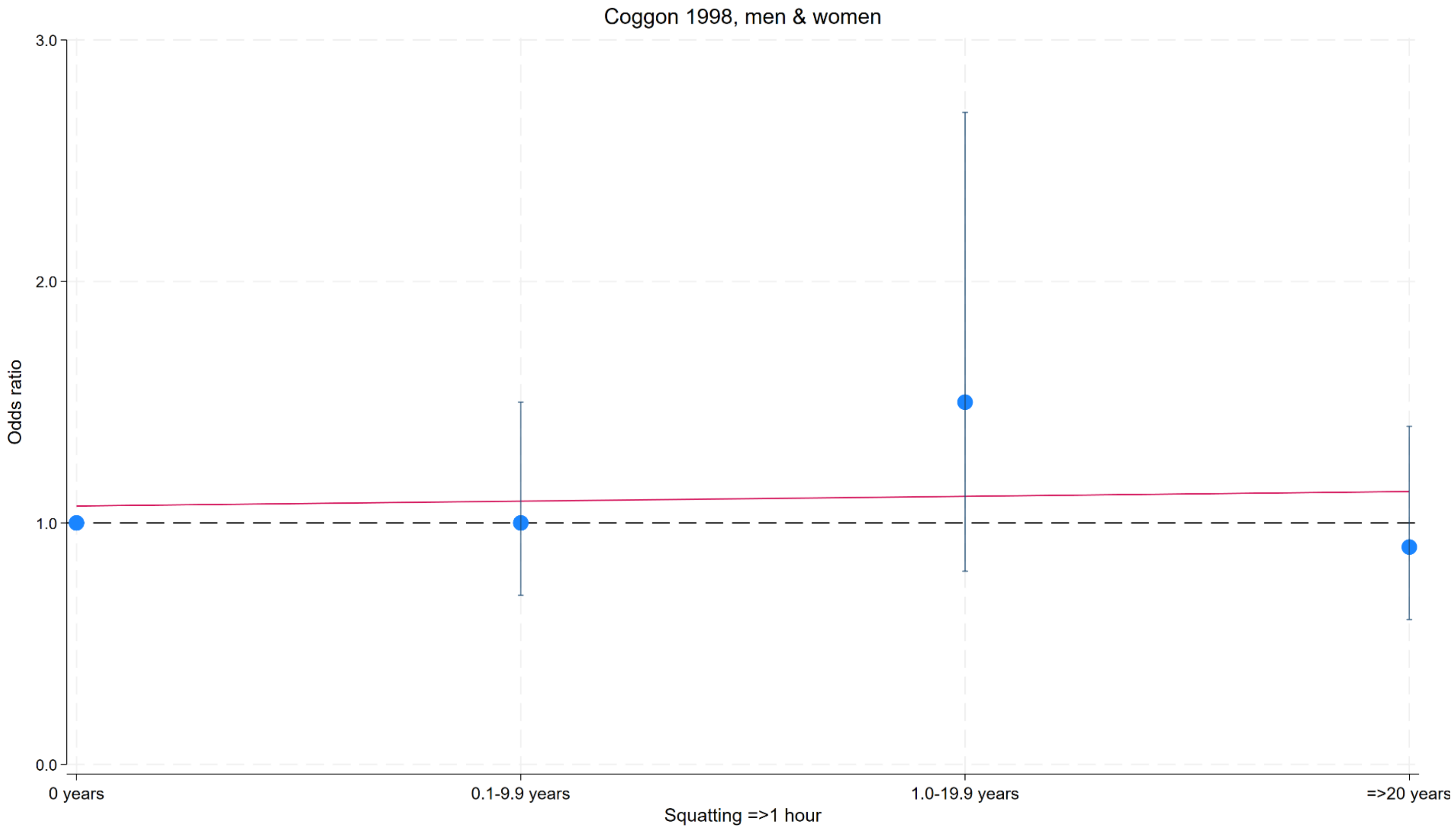

Figure 11. Standing/walking

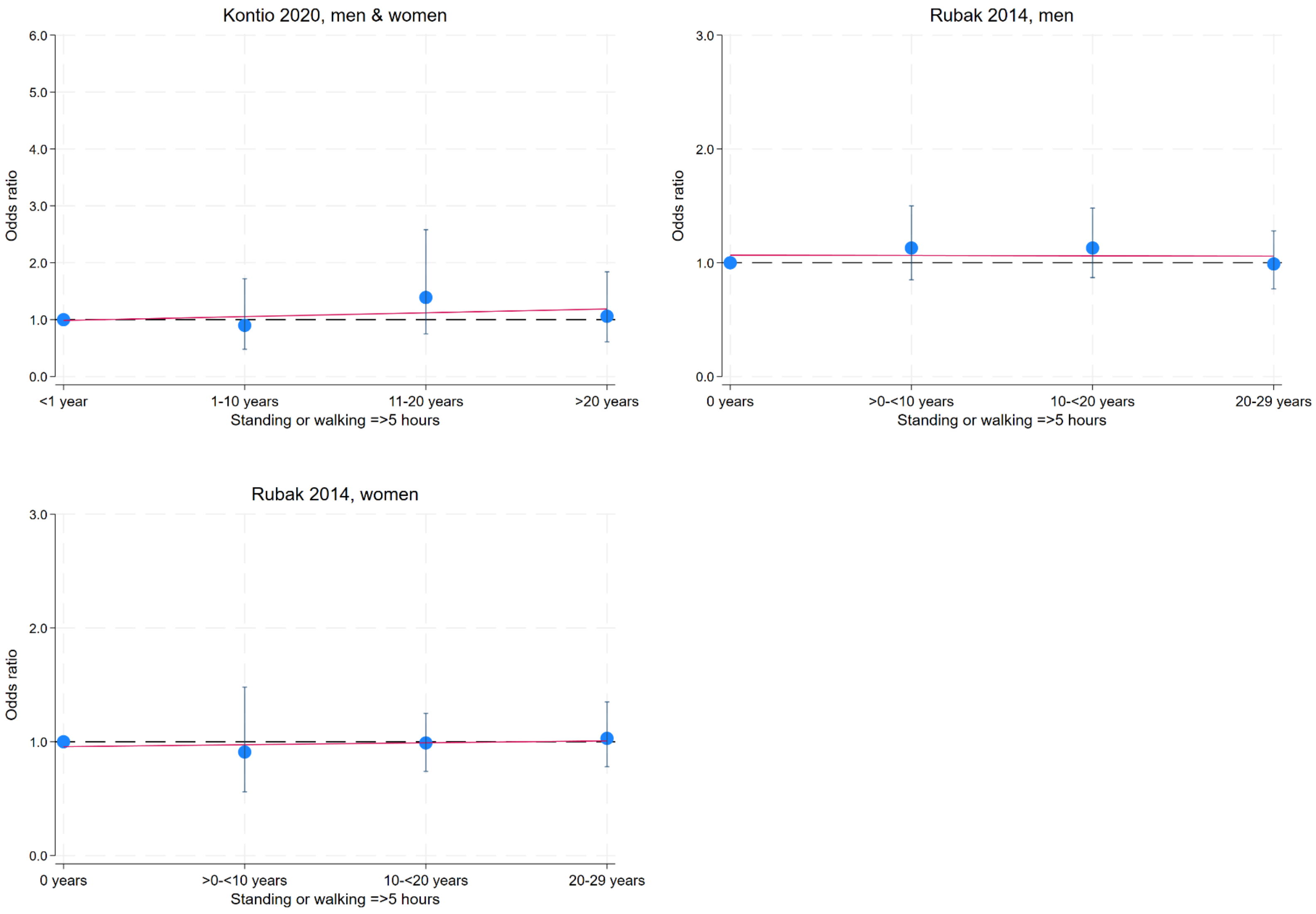

Figure 12. Kneeling/squatting

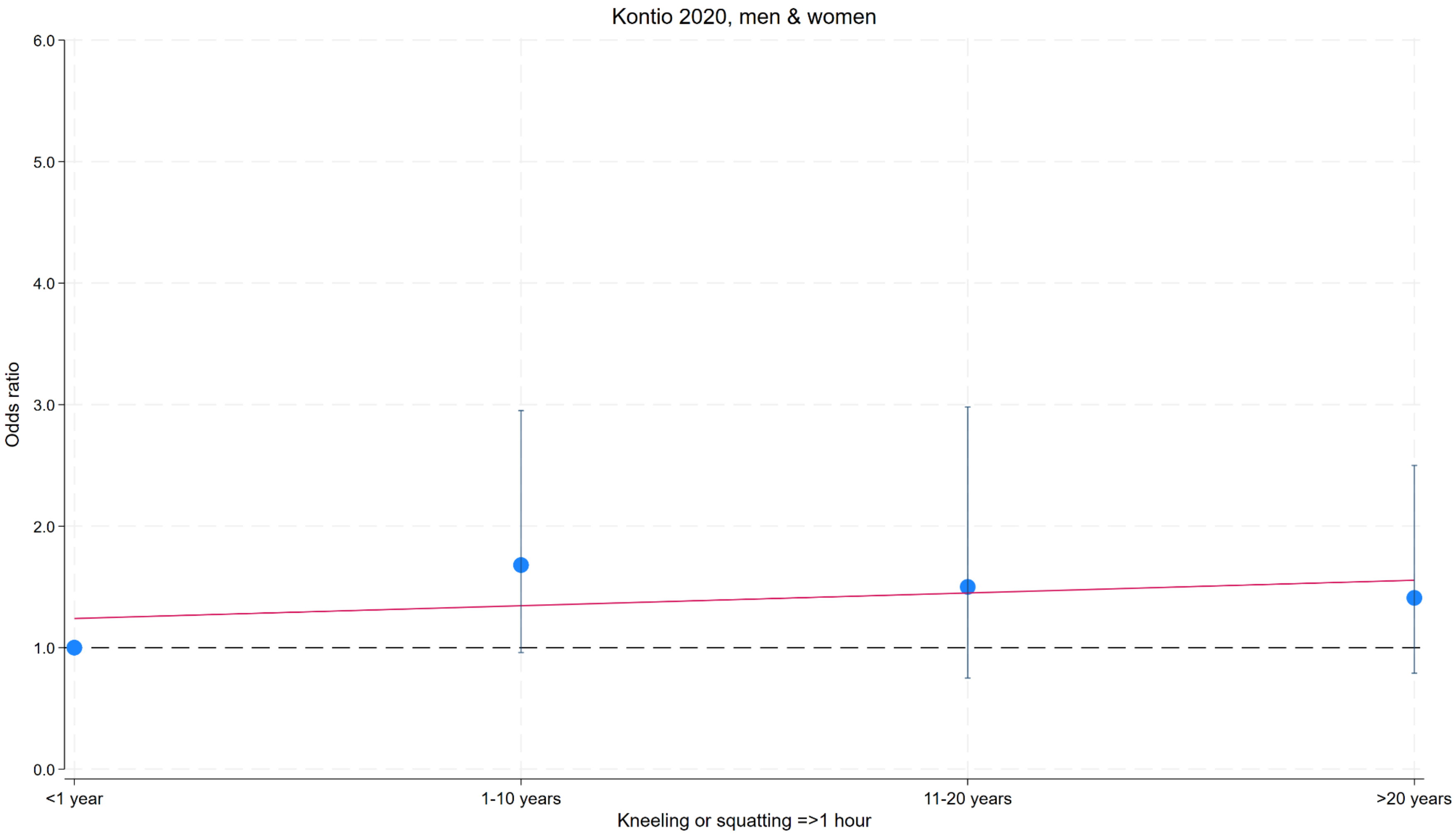

Figure 13. Combined exposures

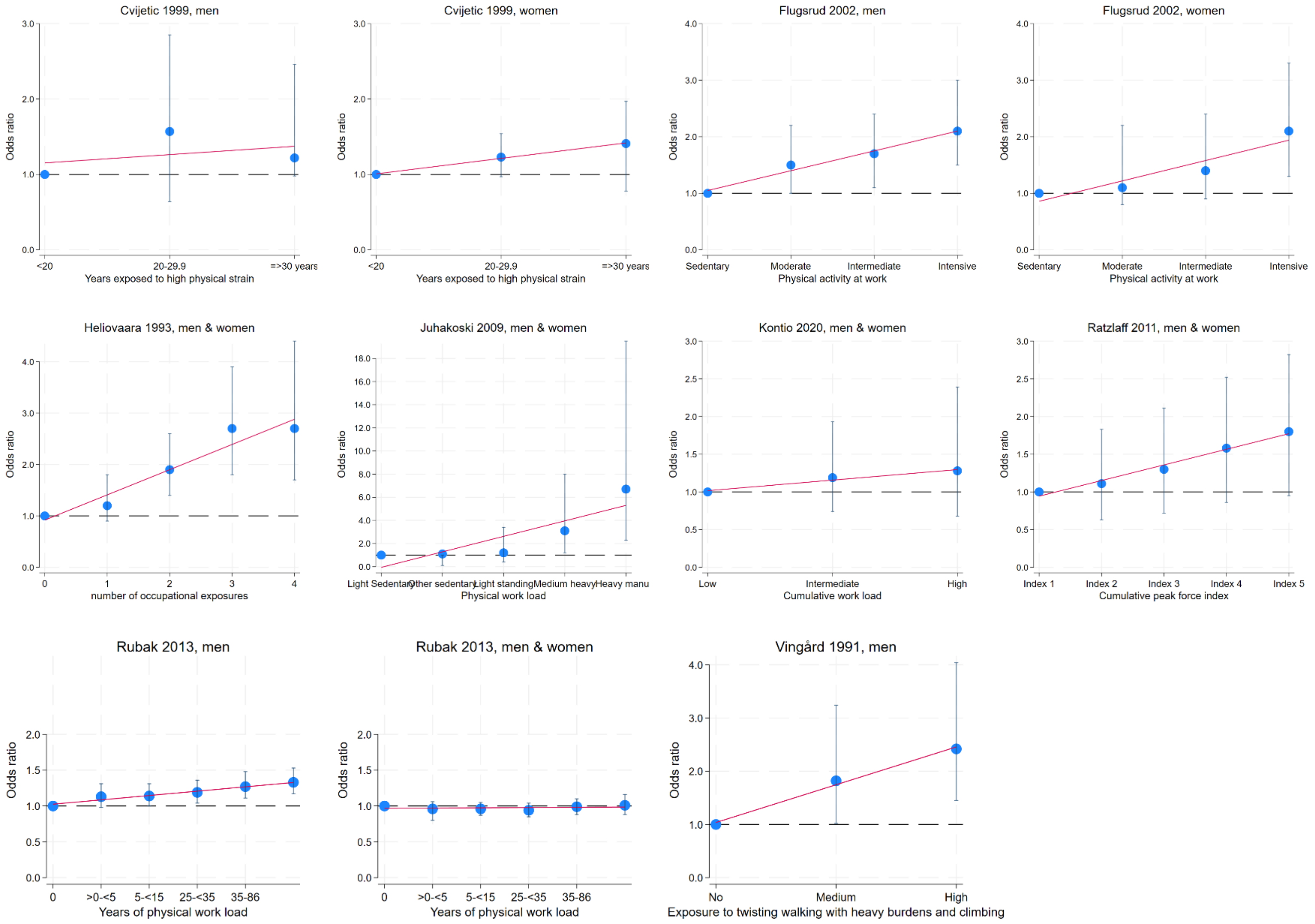

Appendix 9. Forest plots

Figure 14. Standing

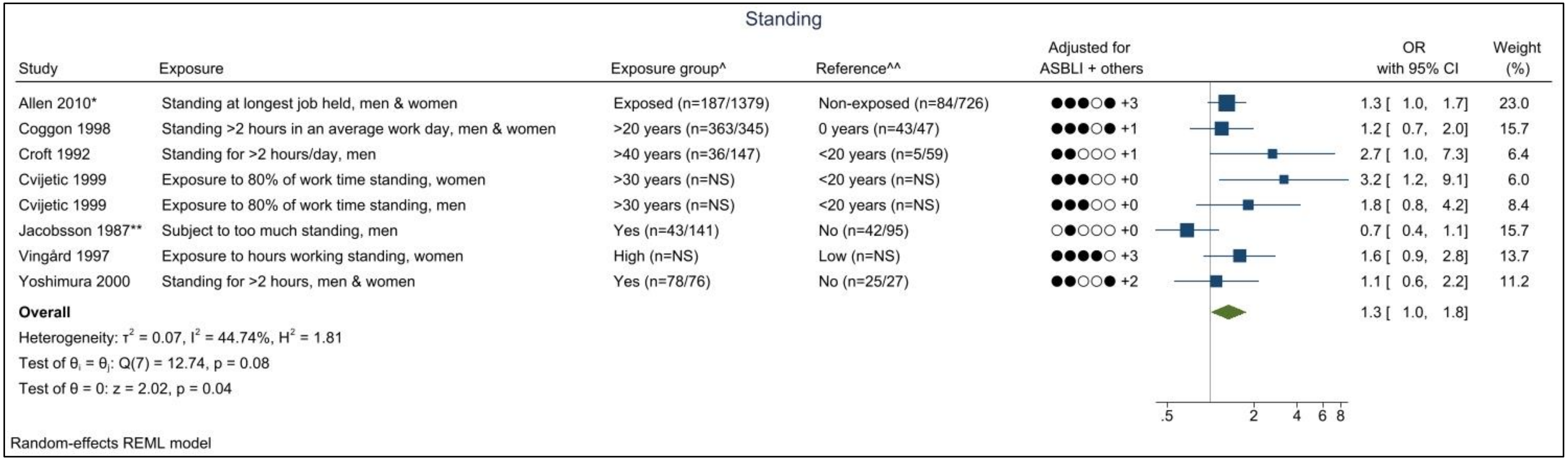

Notes: adjusted variables (ASBLI) = age, sex, body mass index, leisure time activities, and previous injuries in lower extremities. + others refer to adjusting for other confounding factors besides the ASBLI-factors. **Abbreviations**: NS = not specified, OR = odds ratio.
* Allen 2010 - odds ratio calculated based on prevalence of distribution between groups (table 4 in the study).
** Jacobsson 1987 - odds ratio calculated based on numbers of participants (table 1 in the study).
^ Numbers in brackets states numbers of exposed persons with hip OA and numbers of exposed references.
^^ Numbers in brackets states numbers of unexposed persons with hip OA and numbers of unexposed references.

Figure 15. Walking

Walking

Study	Exposure	Exposure group^	Reference^^	Adjusted for ASBLI + others	OR with 95% CI	Weight (%)
Allen 2010*	Walking >50% of the time at work, men & women	Exposed (n=131/914)	Non-exposed (n=160/1197)	●●●○● +3	1.2 [0.9, 1.6]	41.9
Coggon 1998	Walking >3.2 km in an average work day, men & women	>20 years (n=228/222)	0 years (n=140/167)	●●●○● +1	1.3 [0.9, 1.8]	26.4
Croft 1992	Walking for >3.2 km/day, men	>20 years (n=31/146)	<1 year (n=4/32)	●●○○○ +1	1.6 [0.5, 5.1]	2.3
Jacobsson 1987**	Subject to too much walking, men	Yes (n=68/179)	No (n=17/57)	○●○○○ +0	1.3 [0.7, 2.3]	8.5
Lau 2007	Walking for >2 hours per day, men & women	Yes (n=90/220)	No (n=48/194)	●●●○● +0	1.4 [0.9, 2.3]	14.4
Yoshimura 2000	Walking for >3 km, main job, men & women	Yes (n=28/24)	No (n=75/79)	●●○○● +2	1.2 [0.6, 2.4]	6.6
**Overall**					1.3 [1.1, 1.5]	

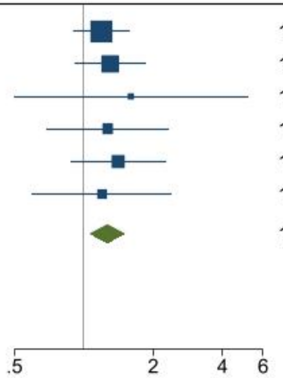

Heterogeneity: $\tau^2 = 0.00$, $I^2 = 0.00\%$, $H^2 = 1.00$

Test of $\theta_i = \theta_j$: Q(5) = 0.60, p = 0.99

Test of $\theta = 0$: z = 2.58, p = 0.01

Random-effects REML model

Notes: adjusted variables (ASBLI) = age, sex, body mass index, leisure time activities, and previous injuries in lower extremities. + others refer to adjusting for other confounding factors besides the ASBLI-factors. **Abbreviations**: km = kilometer, OR = odds ratio.

* Allen 2010 - odds ratio calculated based on prevalence of distribution between groups (table 4 in the study).

** Jacobsson 1987 - odds ratio calculated based on numbers of participants (table 1 in the study).

^ Numbers in brackets states numbers of exposed persons with hip OA and numbers of exposed references.

^^ Numbers in brackets states numbers of unexposed persons with hip OA and numbers of unexposed references.

Figure 16. Climbing stairs

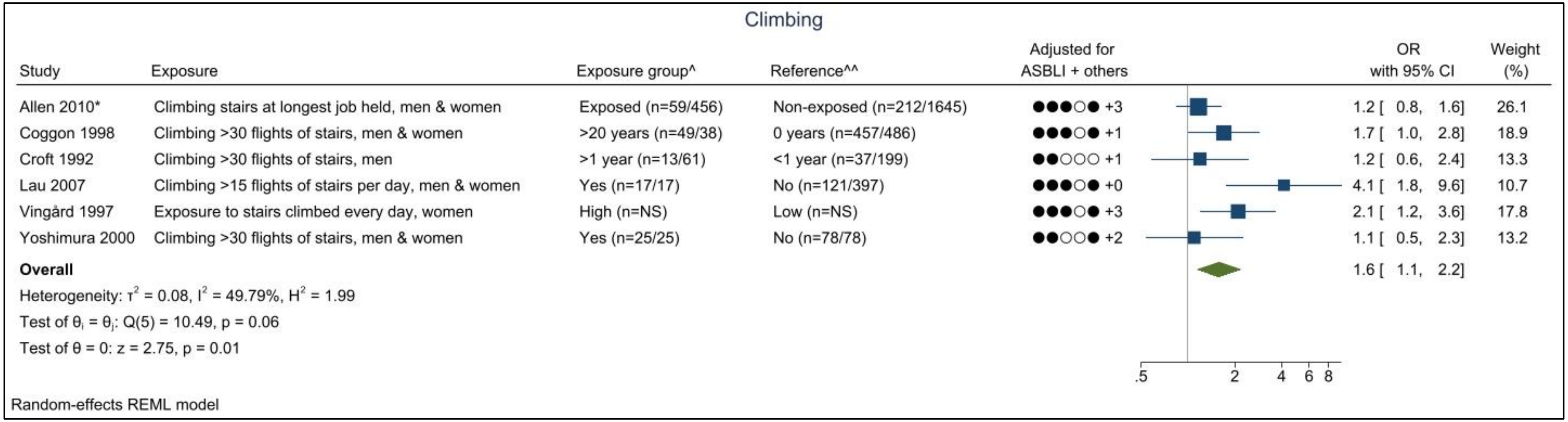

Notes: adjusted variables (ASBLI) = age, sex, body mass index, leisure time activities, and previous injuries in lower extremities. + others refer to adjusting for other confounding factors besides the ASBLI-factors. **Abbreviations**: NS = not specified, OR = odds ratio.

* Allen 2010 - odds ratio calculated based on prevalence of distribution between groups (table 4 in the study).

^ Numbers in brackets states numbers of exposed cases and numbers of exposed references.

^^ Numbers in brackets states numbers of unexposed cases and numbers of unexposed references.

Figure 17. Non-neutral postures

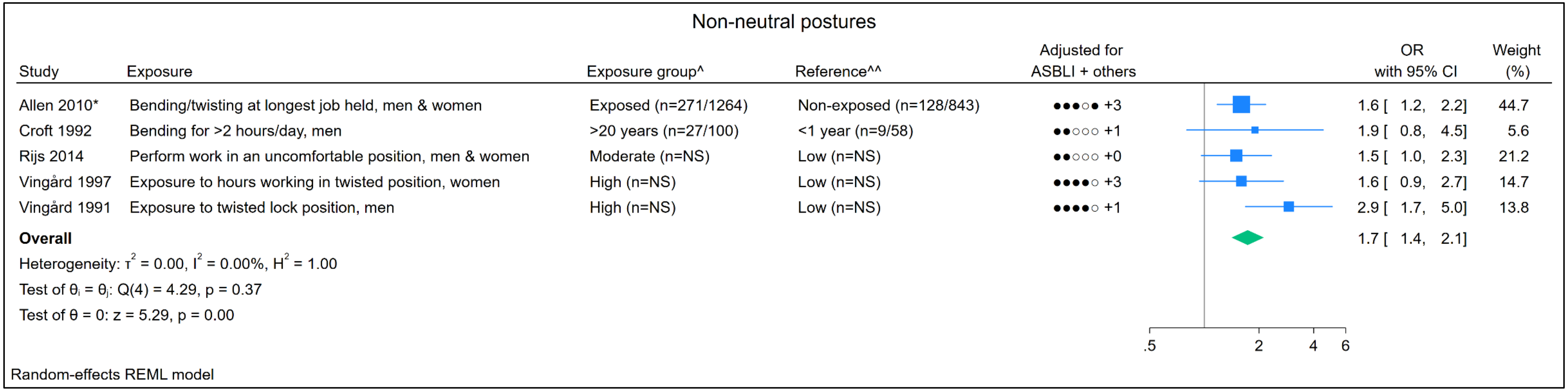

Notes: adjusted variables (ASBLI) = age, sex, body mass index, leisure time activities, and previous injuries in lower extremities. + others refer to adjusting for other confounding factors besides the ASBLI-factors. **Abbreviations**: NS = not specified, OR = odds ratio.

* Allen 2010 - odds ratio calculated based on prevalence of distribution between groups (table 4 in the study).

^ Numbers in brackets states numbers of exposed cases and numbers of exposed references.

^^ Numbers in brackets states numbers of unexposed cases and numbers of unexposed references.

Figure 18. Sitting

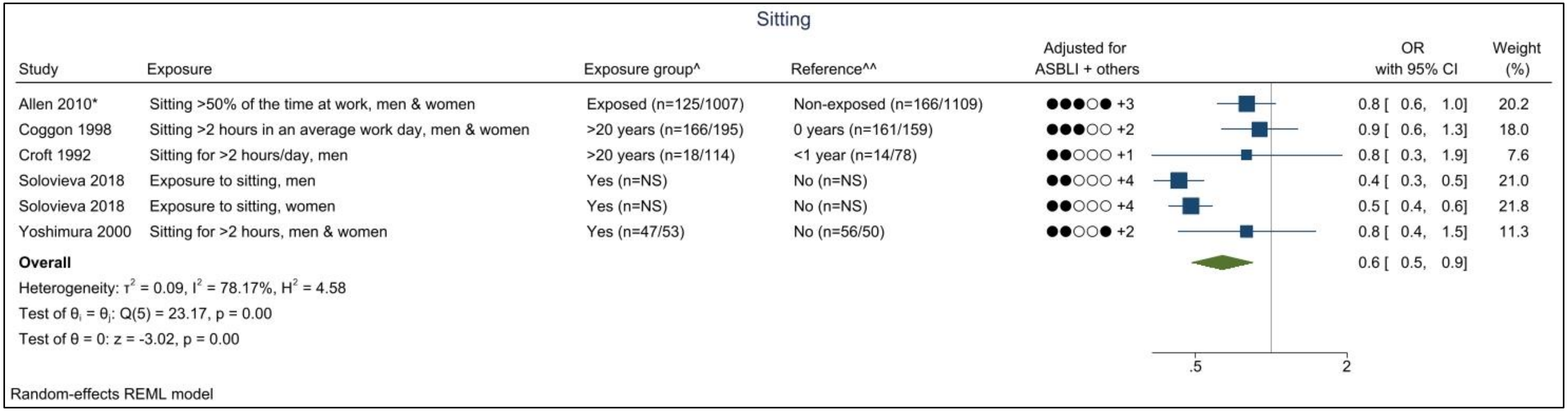

Notes: adjusted variables (ASBLI) = age, sex, body mass index, leisure time activities, and previous injuries in lower extremities. + others refer to adjusting for other confounding factors besides the ASBLI-factors. **Abbreviations**: NS = not specified, OR = odds ratio.

* Allen 2010 - odds ratio calculated based on prevalence of distribution between groups (table 4 in the study).

^ Numbers in brackets states numbers of exposed cases and numbers of exposed references.

^^ Numbers in brackets states numbers of unexposed cases and numbers of unexposed references.

Figure 19. Kneeling

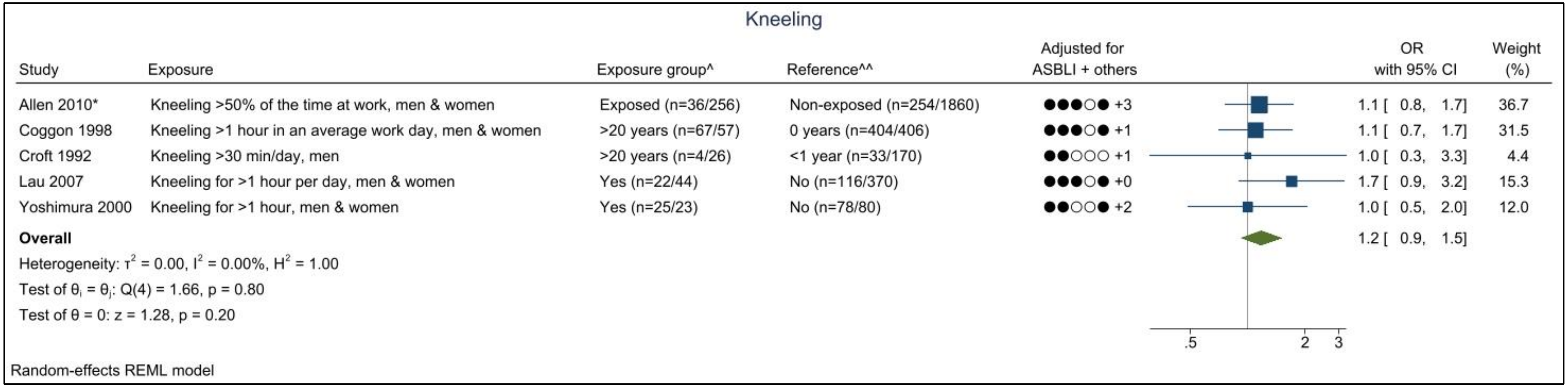

Notes: adjusted variables (ASBLI) = age, sex, body mass index, leisure time activities, and previous injuries in lower extremities. + others refer to adjusting for other confounding factors besides the ASBLI-factors. **Abbreviations**: OR = odds ratio

* Allen 2010 - odds ratio calculated based on prevalence of distribution between groups (table 4 in the study).

^ Numbers in brackets states numbers of exposed cases and numbers of exposed references.

^^ Numbers in brackets states numbers of unexposed cases and numbers of unexposed references.

Figure 20. Squatting

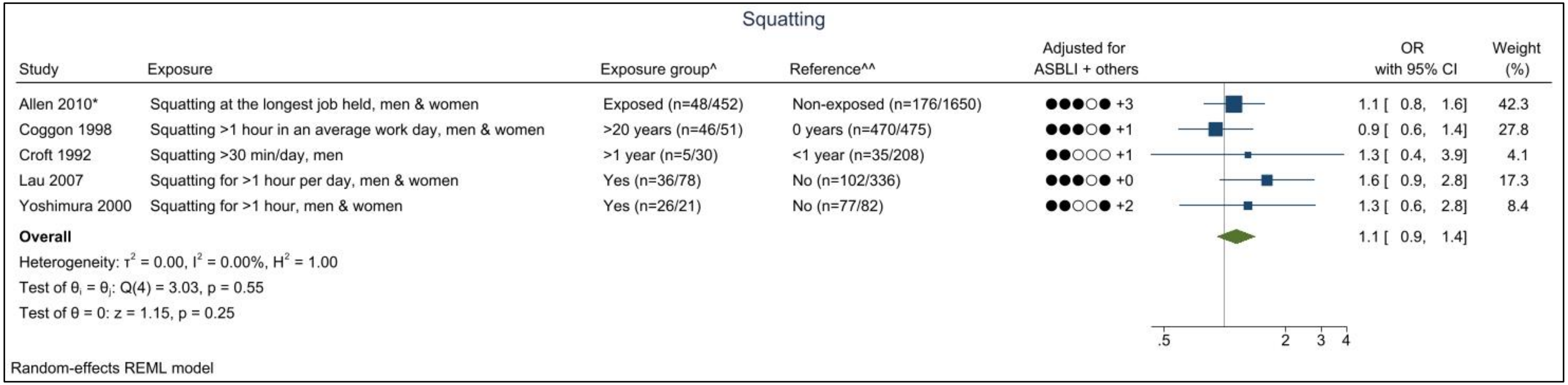

Notes: adjusted variables (ASBLI) = age, sex, body mass index, leisure time activities, and previous injuries in lower extremities. + others refer to adjusting for other confounding factors besides the ASBLI-factors. **Abbreviations**: OR = odds ratio.

* Allen 2010 - odds ratio calculated based on prevalence of distribution between groups (table 4 in the study).

^ Numbers in brackets states numbers of exposed cases and numbers of exposed references.

^^ Numbers in brackets states numbers of unexposed cases and numbers of unexposed references.

Figure 21. Standing/walking

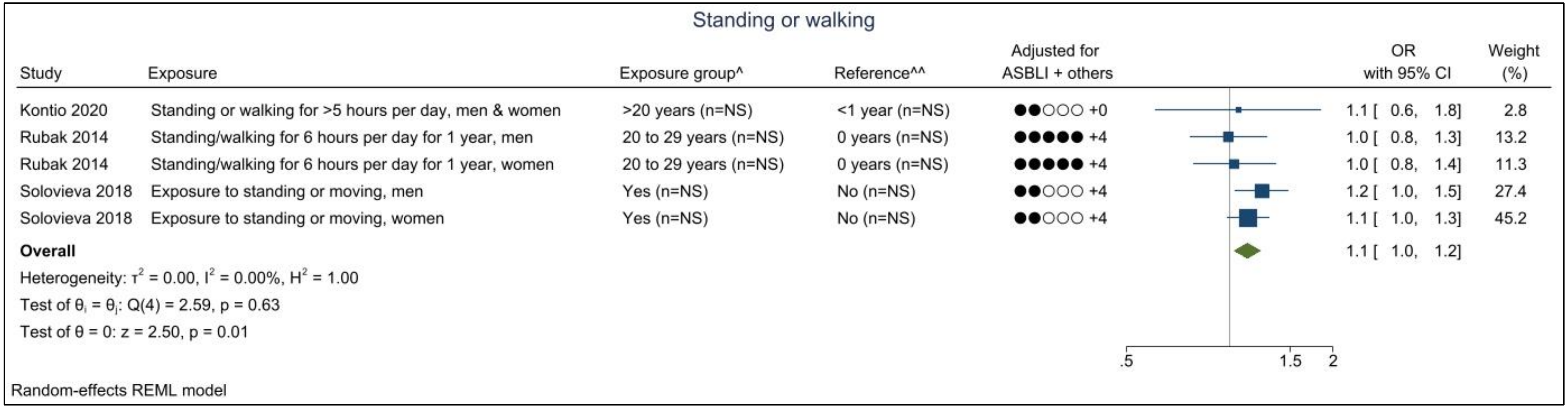

Notes: adjusted variables (ASBLI) = age, sex, body mass index, leisure time activities, and previous injuries in lower extremities. + others refer to adjusting for other confounding factors besides the ASBLI-factors. **Abbreviations**: NS = not specified, OR = odds ratio.
^ Numbers in brackets states numbers of exposed cases and numbers of exposed references.
^^ Numbers in brackets states numbers of unexposed cases and numbers of unexposed references.

Figure 22. Kneeling/squatting

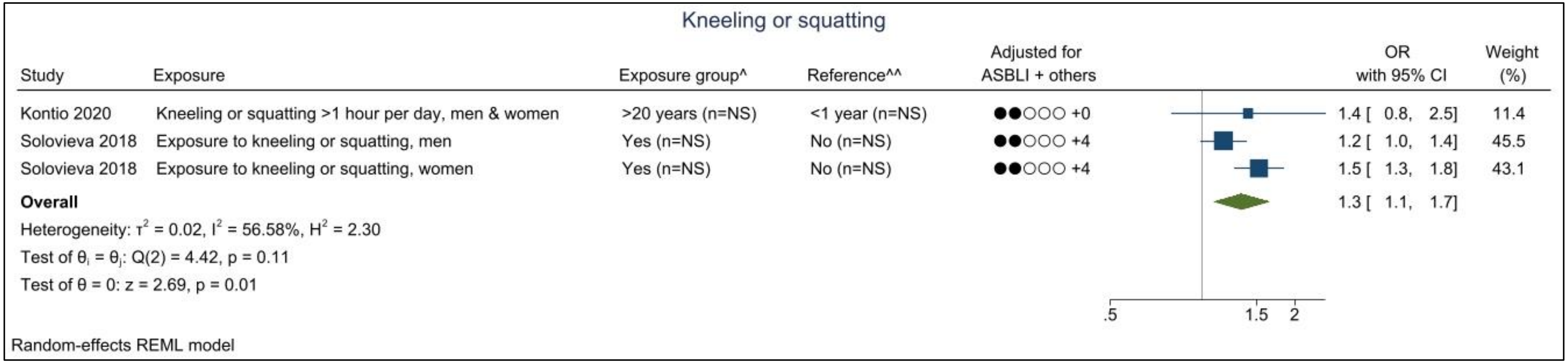

Notes: adjusted variables (ASBLI) = age, sex, body mass index, leisure time activities, and previous injuries in lower extremities. + others refer to adjusting for other confounding factors besides the ASBLI-factors. **Abbreviations**: NS = not specified, OR = odds ratio.
^ Numbers in brackets states numbers of exposed cases and numbers of exposed references.
^^ Numbers in brackets states numbers of unexposed cases and numbers of unexposed references.

(1) Woodruff TJ, Sutton P. The Navigation Guide systematic review methodology: a rigorous and transparent method for translating environmental health science into better health outcomes. Environ Health Perspect. 2014;122(10):1007-14.

(2) Hulshof CTJ, Colosio C, Daams JG, Ivanov ID, Prakash KC, Kuijer PPFM, Leppink N, Mandic-Rajcevic S, Masci F, van der Molen HF, Neupane S, Nygård CH, Oakman J, Pega F, Proper K, Prüss-Üstün AM, Ujita Y, Frings-Dresen MHW. WHO/ILO work-related burden of disease and injury: Protocol for systematic reviews of exposure to occupational ergonomic risk factors and of the effect of exposure to occupational ergonomic risk factors on osteoarthritis of hip or knee and selected other musculoskeletal diseases. Environ Int. 2019 Apr;125:554-566. doi: 10.1016/j.envint.2018.09.053. Epub 2018 Dec 22. PMID: 30583853; PMCID: PMC7794864.
